# Analysis of DNA methylation at birth and in childhood reveals changes associated with season of birth and latitude

**DOI:** 10.1186/s13148-023-01542-5

**Published:** 2023-09-11

**Authors:** Latha Kadalayil, Md. Zahangir Alam, Cory Haley White, Akram Ghantous, Esther Walton, Olena Gruzieva, Simon Kebede Merid, Ashish Kumar, Ritu P. Roy, Olivia Solomon, Karen Huen, Brenda Eskenazi, Peter Rzehak, Veit Grote, Jean-Paul Langhendries, Elvira Verduci, Natalia Ferre, Darek Gruszfeld, Lu Gao, Weihua Guan, Xuehuo Zeng, Enrique F. Schisterman, John F. Dou, Kelly M. Bakulski, Jason I. Feinberg, Munawar Hussain Soomro, Giancarlo Pesce, Nour Baiz, Elena Isaevska, Michelle Plusquin, Marina Vafeiadi, Theano Roumeliotaki, Sabine A. S. Langie, Arnout Standaert, Catherine Allard, Patrice Perron, Luigi Bouchard, Evelien R. van Meel, Janine F. Felix, Vincent W. V. Jaddoe, Paul D. Yousefi, Cecilia H. Ramlau-Hansen, Caroline L. Relton, Elmar W. Tobi, Anne P. Starling, Ivana V. Yang, Maria Llambrich, Gillian Santorelli, Johanna Lepeule, Lucas A. Salas, Mariona Bustamante, Susan L. Ewart, Hongmei Zhang, Wilfried Karmaus, Stefan Röder, Ana Claudia Zenclussen, Jianping Jin, Wenche Nystad, Christian M. Page, Maria Magnus, Dereje D. Jima, Cathrine Hoyo, Rachel L. Maguire, Tuomas Kvist, Darina Czamara, Katri Räikkönen, Tong Gong, Vilhelmina Ullemar, Sheryl L. Rifas-Shiman, Emily Oken, Catarina Almqvist, Robert Karlsson, Jari Lahti, Susan K. Murphy, Siri E. Håberg, Stephanie London, Gunda Herberth, Hasan Arshad, Jordi Sunyer, Regina Grazuleviciene, Dana Dabelea, Régine P. M. Steegers-Theunissen, Ellen A. Nohr, Thorkild I. A. Sørensen, Liesbeth Duijts, Marie-France Hivert, Vera Nelen, Maja Popovic, Manolis Kogevinas, Tim S. Nawrot, Zdenko Herceg, Isabella Annesi-Maesano, M. Daniele Fallin, Edwina Yeung, Carrie V. Breton, Berthold Koletzko, Nina Holland, Joseph L. Wiemels, Erik Melén, Gemma C. Sharp, Matt J. Silver, Faisal I. Rezwan, John W. Holloway

**Affiliations:** 1 Clinical and Experimental Sciences, Faculty of Medicine, University of Southampton, Southampton, UK; 2 Human Development and Health, Faculty of Medicine, University of Southampton, Southampton General Hospital, Southampton, UK; 3 Department of Computer Science and Engineering, Bangladesh University of Engineering and Technology, Dhaka, Bangladesh; 4 Merck Exploratory Science Center in Cambridge MA, Merck Research Laboratories, Cambridge, MA 02141 USA; 5 Epigenomics and Mechanisms Branch, International Agency for Research on Cancer, Lyon, France; 6 Department of Psychology, University of Bath, Bath, UK; 7 Institute of Environmental Medicine, Karolinska Institutet, Stockholm, Sweden; 8 Centre for Occupational and Environmental Medicine, Region Stockholm, Sweden; 9 Department of Clinical Science and Education, Södersjukhuset, Karolinska Institutet, Stockholm, Sweden; 10 Helen Diller Family Comprehensive Cancer Center University of California, San Francisco, CA 94143 USA; 11 Computational Biology and Informatics Core, University of California, San Francisco, CA 94143 USA; 12 Children’s Environmental Health Laboratory, University of California, Berkeley, CA USA; 13 Division of Metabolic and Nutritional Medicine, Dr. von Hauner Children’s Hospital, Ludwig-Maximilians Universität München (LMU), Munich, Germany; 14 CHC, St Vincent, Liège-Rocourt, Belgium; 15 Department of Pediatrics, Vittore Buzzi Children Hospital, University of Milan, Milan, Italy; 16 Pediatric Nutrition and Human Development Research Unit, Universitat Rovira i Virgili, IISPV, Reus, Spain; 17 Neonatal Department, Children’s Memorial Health Institute, Warsaw, Poland; 18 Department of Preventive Medicine, University of Southern California, Los Angeles, CA USA; 19 Division of Biostatistics, School of Public Health, University of Minnesota, A460 Mayo Building, MMC 303, 420 Delaware St. SE, Minneapolis, MN 55455 USA; 20 Glotech, Inc., Rockville, MD USA; 21 Department of Biostatistics, Epidemiology and Informatics, Perelman School of Medicine, University of Pennsylvania, 423 Guardian Drive, Philadelphia, PA 19104 USA; 22 Department of Epidemiology, School of Public Health, University of Michigan, Ann Arbor, USA; 23 Wendy Klag Center for Autism and Developmental Disabilities Johns Hopkins University, Baltimore, MD USA; 24 Department of Mental Health, Bloomberg School of Public Health, Johns Hopkins University, Baltimore, MD USA; 25 Sorbonne Université and INSERM, Epidemiology of Allergic and Respiratory Diseases Department, Pierre Louis Institute of Epidemiology and Public Health (IPLESP UMRS 1136), Saint-Antoine Medical School, Paris Cedex 12, France; 26 Department of Community Medicine and Public Health, SMBB Medical University, Larkana, Pakistan; 27 Institut Desbrest de Santé Publique (IDESP), INSERM and Montpellier University, Montpellier, France; 28 Cancer Epidemiology Unit, Department of Medical Sciences, University of Turin, CPO Piemonte, Italy; 29 Center for Environmental Sciences, University of Hasselt, 3590 Diepenbeek, Belgium; 30 Department of Social Medicine, School of Medicine, University of Crete, Heraklion, Greece; 31 Unit Health, Flemish Institute for Technological Research (VITO), Mol, Belgium; 32 Faculty of Sciences, Hasselt University, Diepenbeek, Belgium; 33 Department of Pharmacology and Toxicology, School for Nutrition and Translational Research in Metabolism (NUTRIM), Maastricht University, Limburg, The Netherlands; 34 Centre de Recherche du Centre Hospitalier de l’Universite de Sherbrooke, Sherbrooke, Canada; 35 Department of Medicine, Universite de Sherbrooke, Sherbrooke, Canada; 36 Department of Biochemistry and Functional Genomics, Universite de Sherbrooke, Sherbrooke, Canada; 37 Clinical Department of Laboratory Medicine, Centre intégré universitaire de santé et de services sociaux (CIUSSS) du Saguenay-Lac-Saint-Jean – Hôpital de Chicoutimi, Chicoutimi, Canada; 38 The Generation R Study Group, Erasmus MC, University Medical Center Rotterdam, Rotterdam, The Netherlands; 39 Division of Respiratory Medicine and Allergology, Department of Pediatrics, Erasmus MC, University Medical Center Rotterdam, Rotterdam, The Netherlands; 40 Department of Pediatrics, Erasmus MC, University Medical Center Rotterdam, Rotterdam, The Netherlands; 41 Medical Research Council Integrative Epidemiology Unit, University of Bristol, Bristol, UK; 42 Population Health Science, Bristol Medical School, University of Bristol, Bristol, UK; 43 Department of Public Health, Aarhus University, Aarhus, Denmark; 44 Periconceptional Epidemiology, Department of Obstetrics and Gynecology, Erasmus MC, University Medical Center, PO Box 2040, 3000 CA Rotterdam, The Netherlands; 45 Life Course Epidemiology of Adiposity and Diabetes (LEAD) Center, University of Colorado Anschutz Medical Campus, Aurora, CO USA; 46 Department of Epidemiology, Colorado School of Public Health, University of Colorado Anschutz Medical Campus, Aurora, CO USA; 47 Department of Epidemiology, University of North Carolina at Chapel Hill, Chapel Hill, NC USA; 48 Division of Biomedical Informatics and Personalized Medicine, Department of Medicine, University of Colorado Anschutz Medical Campus, Aurora, CO USA; 49 Center for Genes, Environment and Health, National Jewish Health, Denver, CO USA; 50 Institute for Global Health (ISGlobal), Barcelona, Spain; 51 Universitat Pompeu Fabra (UPF), Barcelona, Spain; 52 CIBER Epidemiología y Salud Pública (CIBERESP), Madrid, Spain; 53 Bradford Institute for Health Research, Bradford, UK; 54 Institute for Advanced Biosciences, University Grenoble-Alpes, INSERM, CNRS, Grenoble, France; 55 Department of Epidemiology, Geisel School of Medicine, Dartmouth College, Lebanon, NH USA; 56 Center for Molecular Epidemiology, Geisel School of Medicine, Dartmouth College, Lebanon, NH USA; 57 Children’s Environmental Health and Disease Prevention Research Center at Dartmouth, Lebanon, NH USA; 58 Department of Large Animal Clinical Sciences, College of Veterinary Medicine, Michigan State University, East Lansing, MI USA; 59 Division of Epidemiology, Biostatistics, and Environmental Health, School of Public Health, University of Memphis, Memphis, USA; 60 Department of Environmental Immunology, Helmholtz Centre for Environmental Research - UFZ, Leipzig, Germany; 61 2530 Meridian Pkwy, Suite 200, Durham, NC 27713 USA; 62 Department of Chronic Diseases and Ageing, Norwegian Institute of Public Health, Oslo, Norway; 63 Centre for Fertility and Health, Norwegian Institute of Public Health, Oslo, Norway; 64 Section for Statistics and Data Science, Department of Mathematics, Faculty of Mathematics and Natural Sciences, University of Oslo, Oslo, Norway; 65 Center for Human Health and the Environment, North Carolina State University, Raleigh, NC USA; 66 Bioinformatics Research Center, North Carolina State University, Raleigh, NC USA; 67 Department of Biological Sciences, North Carolina State University, Raleigh, NC USA; 68 Department of Obstetrics and Gynaecology, Duke University Medical Center, Durham, NC USA; 69 Department of Psychology and Logopedics, University of Helsinki, Helsinki, Finland; 70 Department of Translational Research in Psychiatry, Max-Planck-Institute of Psychiatry, 80804 Munich, Germany; 71 Department of Medical Epidemiology and Biostatistics, Karolinska Institutet, Stockholm, Sweden; 72 Department of Population Medicine, Harvard Medical School, Harvard Pilgrim Health Care Institute, Boston, USA; 73 Pediatric Allergy and Pulmonology Unit at Astrid Lindgren Children’s Hospital, Karolinska University Hospital, Stockholm, Sweden; 74 Department of Health and Human Services, National Institute of Environmental Health Sciences, National Institutes of Health, RTP, NC 27709 USA; 75 David Hide Asthma and Allergy Research Centre, Isle of Wight, UK; 76 NIHR Southampton Biomedical Research Centre, Southampton General Hospital, Southampton, UK; 77 Department of Environmental Science, Vytautas Magnus University, 44248 Kaunas, Lithuania; 78 Department of Pediatrics, School of Medicine, University of Colorado Anschutz Medical Campus, Aurora, CO USA; 79 Department of Clinical Research, Odense Universitetshospital, Odense, Denmark; 80 Novo Nordisk Foundation Center for Basic Metabolic Research, Faculty of Health and Medical Sciences, University of Copenhagen, Copenhagen, Denmark; 81 Department Public Health, Faculty of Health and Medical Sciences, University of Copenhagen, Copenhagen, Denmark; 82 Division of Neonatology, Department of Pediatrics, Erasmus MC, University Medical Center Rotterdam, Rotterdam, The Netherlands; 83 Diabetes Unit, Massachusetts General Hospital, Boston, MA USA; 84 Provincial Institute for Hygiene, Antwerp, Belgium; 85 Department of Public Health and Primary Care, Leuven University, Louvain, Belgium; 86 Epidemiology Branch, Division of Population Health Research, Division of Intramural Research, Eunice Kennedy Shriver National Institute of Child Health and Human Development, 6710B Rockledge Dr, MSC 7004, Bethesda, MD USA; 87 Center for Genetic Epidemiology, University of Southern California, Los Angeles, CA 90033 USA; 88 Norris Comprehensive Cancer Center, University of Southern California, Los Angeles, CA 90033 USA; 89 Sachs’ Children and Youth Hospital, Södersjukhuset, Stockholm, Sweden; 90 School of Psychology, University of Exeter, Exeter, UK; 91 Medical Research Council Unit, The Gambia at the London School of Hygiene and Tropical Medicine, Fajara, The Gambia; 92 Medical Research Council Unit, The Gambia at the London School of Hygiene and Tropical Medicine, London, UK; 93 Department of Computer Science, Aberystwyth University, Aberystwyth, Ceredigion UK; 94 Children’s Environmental Health Laboratory, CERCH, Berkeley Public Health, University of California, 2121 Berkeley Way #5216, Berkeley, CA 94720 USA

**Keywords:** PACE, Meta-analysis, Birth season, DNA methylation, Differentially methylated regions (DMR), Latitude

## Abstract

**Background:**

Seasonal variations in environmental exposures at birth or during gestation are associated with numerous adult traits and health outcomes later in life. Whether DNA methylation (DNAm) plays a role in the molecular mechanisms underlying the associations between birth season and lifelong phenotypes remains unclear.

**Methods:**

We carried out epigenome-wide meta-analyses within the Pregnancy And Childhood Epigenetic Consortium to identify associations of DNAm with birth season, both at differentially methylated probes (DMPs) and regions (DMRs). Associations were examined at two time points: at birth (21 cohorts, *N* = 9358) and in children aged 1–11 years (12 cohorts, *N* = 3610). We conducted meta-analyses to assess the impact of latitude on birth season-specific associations at both time points.

**Results:**

We identified associations between birth season and DNAm (False Discovery Rate-adjusted *p* values < 0.05) at two CpGs at birth (winter-born) and four in the childhood (summer-born) analyses when compared to children born in autumn. Furthermore, we identified twenty-six differentially methylated regions (DMR) at birth (winter-born: 8, spring-born: 15, summer-born: 3) and thirty-two in childhood (winter-born: 12, spring and summer: 10 each) meta-analyses with few overlapping DMRs between the birth seasons or the two time points. The DMRs were associated with genes of known functions in tumorigenesis, psychiatric/neurological disorders, inflammation, or immunity, amongst others. Latitude-stratified meta-analyses [higher (≥ 50°N), lower (< 50°N, northern hemisphere only)] revealed differences in associations between birth season and DNAm by birth latitude. DMR analysis implicated genes with previously reported links to schizophrenia (*LAX1*), skin disorders (*PSORS1C*, *LTB4R*), and airway inflammation including asthma (*LTB4R*), present only at birth in the higher latitudes (≥ 50°N).

**Conclusions:**

In this large epigenome-wide meta-analysis study, we provide evidence for (i) associations between DNAm and season of birth that are unique for the seasons of the year (temporal effect) and (ii) latitude-dependent variations in the seasonal associations (spatial effect). DNAm could play a role in the molecular mechanisms underlying the effect of birth season on adult health outcomes.

**Supplementary Information:**

The online version contains supplementary material available at 10.1186/s13148-023-01542-5.

## Background

Plants and animals adapt to altered seasonal cues such as temperature and light by regulating their transcriptional programmes. It has been well established in humans that many traits, including physiological (e.g. blood pressure and cholesterol), behavioural traits (e.g. conception, suicidal tendencies), and life span, display distinct seasonal patterns [1–5]. The incidence of complex diseases such as cardiovascular and autoimmune disease, and psychiatric disorders shows seasonal fluctuations [6–9]. Several studies have demonstrated an association between season of birth and development of disease both in childhood and later in life. These adverse events include asthma and allergy-related diseases [10–13], neonatal immune development [14], multiple sclerosis [15] and schizophrenia [9, 16], suggesting long-lasting effects on a broad range of characteristics. Exposures that vary seasonally such as outdoor temperature, humidity, ultraviolet (UV) light, pollen levels, Vitamin D, melatonin levels, air pollution and availability of nutrients have been hypothesised to drive the season-of-birth-linked disease phenotypes (reviewed in [17]). Uncovering the molecular mechanisms underlying the effect of season of birth on lifelong phenotypes can help elucidate the biological basis for the observed associations.

Epigenetic modifications, including alterations in DNA methylation (DNAm), play a key role in the gene regulation of a variety of cellular processes. Such modifications may also act as molecular mediators between season-linked environmental factors and health outcomes. In plants and animals, the role of DNAm in the regulation of several seasonally associated genes is well documented [18–20]. In humans, the seasonal periodicity of gene expression profiles has been demonstrated for genes involved in immunity and physiology [21]. In addition, there is evidence to suggest that environmental factors alter DNAm levels in a season-specific manner in healthy individuals [22]. It is proposed that these changes in DNAm profiles may influence health outcomes later in life. Taken together, a growing body of evidence suggests a role for differential DNAm in the exposure-induced changes in gene expression and disease [23]. However, studies on the seasonality of DNA methylation at birth in humans have not assessed variations across a wide range of geographies on a large scale.

Early life environmental exposures result in specific changes in DNAm that may persist throughout the life course. For example, maternal smoking in pregnancy results in a specific DNA methylation signature in offspring [24] that persists well into adulthood [25]. Similarly, it is plausible that DNAm changes associated with birth season, a proxy for in utero exposures, could be a mechanistic pathway underlying the effect of season of birth on later life health and disease. Lockett et al. tested the effect of season of birth on the risk of allergic outcomes in adulthood as well as the association between season of birth and differential DNAm at birth, and at age 18 [26]. They demonstrated a season-associated pattern of DNAm at some CpG sites associated with apoptotic genes as well as season-dependent risk patterns of allergic diseases such as eczema and rhinitis at 18 years of age. The study was limited in sample size (*N* = 175) but was validated in a small independent cohort.

Here, we report the results of a meta-analysis of epigenome-wide association studies (EWAS) from 27 independent cohorts (*N* = 12927 participants) from the Pregnancy And Childhood Epigenetics (PACE [27]) consortium. Blood DNAm data at birth and from children aged between 1 and 11 years were used to investigate the association between season of birth and differential blood DNA methylation at these two time points. In addition, we examined if the latitude where the children were born influenced the season-of-birth-associated DNAm profiles as exposures such as sunlight, relative humidity, pollen count, infectious agents and air pollution can vary with latitude in each season.

## Methods

This meta-analysis study intended to investigate the epigenome-wide associations of season of birth and DNAm at two time points, at-birth, and childhood, on a large-scale using data from multiple cohorts across differing geographies.

### Cohort participants

**Fig. 1:**
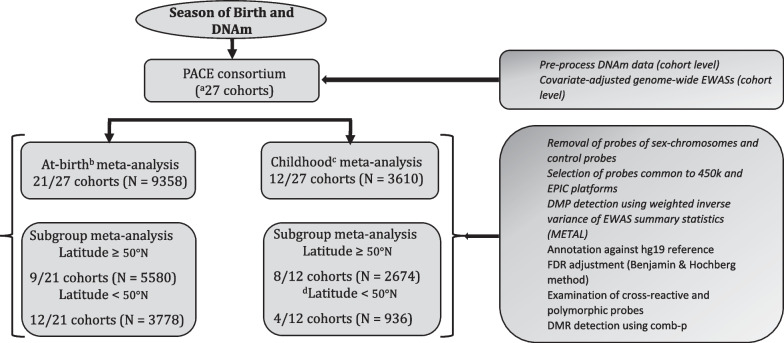
Schematic diagram for the season of birth-DNA methylation (DNAm) analyses. Numbers included in the analyses are indicated. *PACE* Pregnancy And Childhood Epigenetics, *EWAS* Epigenome Wide Association Study, *DMP* Differentially Methylated Probe, *FDR* False Positive Rate, *DMR* Differentially Methylated Region. EWASs and meta-analysis were carried out on at-birth and childhood samples separately. All participating cohorts were from the northern hemisphere (range 37.2–71.2°N). Latitude ≥ 50°N represents a subset of cohorts from 50 to 71.2°N (referred to as “higher latitude subgroup” in the text). Latitude < 50°N is a subset of cohorts from 32.7 to 50°N (referred to as “lower latitude subgroup” in the text. ^a^27 independent cohorts, but a total of 33 datasets as 6 cohorts contributed to both at-birth and childhood analyses. ^b^At-birth blood samples (cord and heel prick). ^c^Whole blood samples (age 1–11 years). ^d^This analysis was not done due to its small sample size

Cohorts participating in the PACE consortium with data on season of birth and DNAm at birth and/or later in childhood were invited to participate in a meta-analysis of associations between season of birth and DNA methylation. Twenty-one independent cohorts (*N* = 9418) contributed to the at-birth and 12 cohorts (*N* = 3610) to the childhood meta-analyses. Most cohorts contributed data either for at-birth or childhood analysis (21/27), while six cohorts (~ 18% of the at-birth data and ~ 45% of the childhood data) contributed to both time points. Less than 20% of the study population had data at two time points from the same child. Data for 60 individuals (EARLI, non-European) were excluded from the at-birth analysis due to insufficient numbers (as per the PACE recommendations of ≥ 15/group) of births in the spring season (*n* (spring) = 4, Additional file 1: Table S1B) resulting in a total of 9358 for the at-birth analysis (Fig. 1). All study participants originated from countries in the northern hemisphere (32.7–71.2°N). Additional file 1 (Table S1A-C) lists all cohorts along with the distributions of covariates used in the cohort-specific EWASs. Additional file 2 provides cohort-specific definition of covariates, DNAm data collection and pre-processing methods.

### Exposures and outcomes

#### Primary analysis

The outcome was DNAm at birth and childhood. For new-born samples, all but two cohorts used cord blood, while CBC and CHS used dried blood spots collected post-delivery (Additional file 1: Table S1). The primary exposure was season of birth as a categorical variable with autumn as the reference season. Seasons were defined as winter (December–February), spring (March–May), summer (June–August) and autumn (September–November) since all participants were from the northern hemisphere.

#### Secondary analysis

Associations of season of birth and DNAm at-birth were also investigated in samples stratified by the cohort latitude to explore its influence on DNAm signals. Cohorts were dichotomized into a “lower latitude subgroup” (32.7–50°N with *N* = 3838) and “higher latitude subgroup” (≥ 50°N with *N* = 5580). The choice of 50° as the cut off was arbitrary, although it provided ~ 8 h of sunlight/day at the winter equinox. We focussed on comparing (i) the impact of latitudes on the pattern of association between season of birth and methylation signals at-birth and (ii) the pattern of season of birth specific associations across the two time points (at birth and in childhood) in each latitude. However, due to the small sample size contributing to childhood DNAm analysis in the lower latitude (*N* = 936), the DNAm results of the child cohorts in the higher latitude subgroup alone were used for comparison with the at-birth DNAm associations.

### Measurement and pre-processing of DNA methylation

Details of cohort-specific methylation assays, data handling, quality control and normalisation are given in Additional file 2. Briefly, DNAm assays were carried out on bisulfite-converted genomic DNA extracted from either cord blood/heel prick samples collected at delivery or shortly after delivery and from whole blood samples from children aged 1–11 years. Methylation data for these samples were generated using illumina Infinium® HumanMethylation450 (most cohorts) or EPIC BeadChip assay (EAGeR, IoWF2, BiB of HELIX, (Additional file 1: Table S1A). All cohorts estimated methylation levels as beta values (*β*) using the formula $\beta = \frac{M}{M + U + 100}$ where *M* and *U* are methylated and unmethylated signal intensities, respectively. Normalised beta values were used in preference to *M* values $\left[ {M = \log_{2} \left( {\frac{\beta }{1 - \beta }} \right) } \right]$ to afford direct comparison of our findings with similar findings in the literature where most epigenetic association studies employ beta values. While all cohorts followed a pre-specified analysis plan including recommendations for bioinformatics pipelines (Additional file 3), the individual cohorts conducted probe filtering, normalisation of methylation data and correction for batch effects using their own preferred methods. Probes on sex chromosomes and control probes were removed prior to meta-analysis. Cross-reactive probes and polymorphic probes were dealt with post-meta-analysis. Each cohort also provided information on genomic inflation in their cohort-specific EWAS models. Leave-one-out analyses were carried out to check if inflation in any one cohort unduly influenced the overall meta-analysis results.

**Table 1:** Differentially methylated CpGs (FDR < 0.05) in the birth seasons in at-birth and childhood analyses

Season of birth^a^	CpG	Coeff	SE	*p* value	FDR *p* value	Lambda^b^	CHR	Position	Mapped gene	Direction^c^	*I*^2^
***At-birth***^***d***^											
Winter	cg26416241	0.0034	0.0006	**6.02** × **10**^−**8**^	0.014	1.02	11	131557414	*NTM*	+ + + − + + + + − + − + + + + − + + − + + + + −	42.5
Spring	cg18978324	0.0006	0.0001	**2.61** × **10**^−**8**^	0.012	1.05	14	24740937	*RABGGTA*	− + + + + + + ? + − + + + + + + ? + − + − + + −	27
***Childhood***											
Summer	cg19416462	− 0.0037	0.0007	**1.99** × **10**^−**8**^	0.009	1.16	6	14533530	*CD83*	− − − − − − − + − − − −	45.9
	cg01656588	− 0.007	0.0013	1.47 × 10^−7^	0.026		5	3187100	*LINC01377*	− − − − − − − − − − − −	0
	cg03263237^e^	− 0.0157	0.003	1.67 × 10^−7^	0.026		16	15227611	*MIR6511B2*	− − ? − − − ? − − ? + ?	24.6
	cg15437053	0.0037	0.0007	2.69 × 10^−7^	0.032		19	34311123	*KCTD15*	+ ? + + + + + + + + + +	32.9

CpGs shown passed the FDR (False discovery rate) *p* value threshold of 5% in meta-analyses of EWAS summary results carried out with 21 and 12 cohorts for at-birth and childhood blood samples, respectively. The *p* values for the CpG sites that also passed the Bonferroni-corrected significance threshold of 1.06 × 10^−7^ are shown in bold. All cohort-specific EWAS analyses were adjusted for sex of the child, gestational age at delivery, maternal age at delivery, maternal smoking during pregnancy, maternal socio-economic status, batch, child’s age at the time of sample collection (in the childhood analyses) and estimated cell proportions

Coeff: regression coefficient (change in mean methylation compared to autumn reference); SE: standard error of the coefficient; Lambda: genomic inflation factor; CHR: chromosome; *I*^2^: a measure of heterogeneity between studies

^a^Reference season for the EWAS analyses of individual cohorts: autumn

^b^Estimated using the BACON method (van Iterson et al. [37])

^c^Hyper-methylation is indicated by “+” and hypo-methylation by “−” (minus sign)

^d^One CpG (in winter-born; cg01801443; *p* value: 7.77 × 10^−10^) with a SNP within 10 base pairs of the probe is not shown in the table

^e^Cross-reactive probe (Chen et al. [46])

### Covariates

All cohort-specific models, at birth or childhood, included the following covariates: maternal smoking status during pregnancy, maternal socio-economic status (SES), maternal age and gestational age at delivery, new-born’s sex, and estimated cell type proportions. These covariates have been reported to be associated with either season of birth or DNA methylation making them potential confounders of the association between season of birth and DNA methylation or both [26, 28–30]. Cell type proportions were estimated by applying the Houseman method [31] to methylation data using cord blood [32] or peripheral blood reference panels [33] for at-birth and childhood analyses, respectively, as these reference-based methods were reported to be the best available methods for studies with large sample sizes [34]. In addition, the childhood models included children’s age and season of sample collection if data were available. Most cohorts used three categories for maternal smoking status during pregnancy (no smoking, quit early during pregnancy and smoked throughout pregnancy), but cohorts lacking this level of detail or with too few who smoked during pregnancy used any versus no smoking. The definition of maternal SES was cohort specific and was mainly based on maternal education, occupation and/or income. Ninety-six percent of the study population were of European ancestry despite being drawn from multiple countries. Ethnicity was, therefore, not controlled for in the models. Cohort-specific covariate data collection methods are described in Additional file 2. In general, most cohorts collected data through questionnaires/interviews and/or from medical records. Individual cohorts used their preferred batch correction methods, and they typically comprised adjustments for methylation profiling-specific variables such as plates, bead chip or other relevant technical covariates. Studies which used a sampling scheme included the sampling variable as a covariate.

### Statistical methods

#### Cohort-specific epigenome-wide association studies (EWAS)

Each cohort performed independent EWAS analysis following a pre-specified analysis plan and an R script (Additional file 3). The primary exposure variable, season-of-birth, was consistently coded by all cohorts with autumn as the reference season. Data on one child per family were included (randomly selected) in the cohort-specific EWAS if multiple new-borns/siblings from the same family were enrolled in the cohort. Only samples with complete data for all covariates were included in EWAS analyses.

Cohort-specific EWASs modelled the association between season of birth (exposure) and DNAm (outcome) at a CpG site of the at-birth or childhood blood DNAm data using analysis of covariance via robust regression applying Huber’s weighting, implemented in the R function rlm(). Birth seasons were defined as winter (December–February), spring (March–May), summer (June–August) and autumn (September–November). Briefly, cohorts generated a regression model comparing children born in spring, summer, and winter against autumn as the reference for each of the two time points (at-birth or childhood for which they had methylation data). Models were adjusted for cohort-specific covariates as described above and in Additional file 2.

### Meta-analyses

Meta-analyses were carried out using fixed effect inverse variance weighted option of METAL [35]. Meta-analyses for at-birth and childhood data were performed separately. All analyses were repeated by one other member of the team independently. Probes that were common to both EPIC and 450 k platforms alone were included in the meta-analysis [36]. A total of 470870 probes were retained after the meta-analyses of EWAS summary results. Each meta-analysis result was checked visually using quantile–quantile (QQ) plots and quantitatively by estimating lambda values, an indicator of inflation. Bias and inflation were estimated following the BACON method of van Iterson et al. [37]. Meta-analysed probes were annotated against hg19 reference genome and mapped to the nearest genes and genomic locations (CpG islands, shores, shelves) using the Illumina 450k manifest or ACME R package [38] when not available in the manifest. The annotated output from meta-analysis holds information on probes which overlap SNPs or are within 10 bases from the CpG sites (polymorphic probes). These were disregarded while interpreting the significantly differentially methylated CpGs. Associations were considered significant for CpGs that reached a statistical significance based on a false discovery rate (FDR) below 5% [39]. The FDR-corrected probes were also checked for a more stringent Bonferroni criterion of satisfying a *p* value threshold of 1.06 × 10^−7^.

### Differentially methylated regions

Differentially methylated region (DMR) analyses were carried out using comb-p in R [40] on the meta-analysed single-CpG EWAS results separately for at-birth and childhood samples. CpGs within 1000 base pairs were combined to define a region [41]. The significance threshold was set to the Šidák-corrected *p* value of 0.05 in all DMR analyses.

### Trait enrichment analysis

Trait enrichment analysis of top CpGs (raw *p* values for association in meta-analyses < 1.0 × 10^−5^) from the primary and latitude analyses was carried out using EWAS Atlas [42]. Traits were considered significant if the *p* values for their odds ratios were less than 0.05.

### Metastable epialleles

Fisher’s exact test was carried out in R (3.5.1) to compare the proportion of metastable epialleles amongst significant loci (*p* value < 0.05) in each of the birth seasons of new-born infants against the proportion in array background. The comparator was 2408 metastable epialleles listed in three separate studies [43–45].

## Results

### Study population

Twenty-seven studies from the PACE consortium participated in the meta-analysis study to investigate possible associations between season of birth and DNAm at birth and in childhood [at-birth: 21 studies, children: 12 studies of which six cohorts contributed to both (Fig. 1, Additional file 1: Table S1A)]. There was a total of 9358 and 3610 participants for the at-birth and childhood analyses, respectively (Fig. 1). A subgroup analysis, carried out on participants living in regions of ≥ 50°N, included 5580 new-borns (9 studies) and 2674 children aged 1–11 years (8 studies) [see Additional file 1: Table S1 (A-C)].

The births were evenly distributed across the seasons (22–27.5%, Additional file 1: Table S1B-C). Approximately 50% of the babies and children included in the analyses were girls. The median maternal age at delivery was 30 years [range (min–max): 24–34.2] for the participants of the at-birth analyses and 30 years (range 26.7–32.1) for the childhood analyses. The median gestational age was 39.5 weeks (range 38.9–40.2) and 39.6 (range 36.7–40.2) for the at-birth and childhood participants, respectively. The proportion of mothers who smoked during pregnancy ranged from 0 to 15% in most cohorts in both the at-birth and childhood samples except for the mothers of IoW F2 (36.7%) and NEST (23.2% and 20% for the two ethnic groups) for the at-birth analyses.

### Association between season of birth and DNA methylation at birth and in childhood

For each time point, at-birth and childhood, the individual cohorts generated an EWAS model (adjusted for covariates) comparing the three seasons (winter, spring, and summer) with autumn as a reference. The lambdas for the six models varied between 1.02 and 1.16 (Additional file 4: Table S2). Meta-analyses were then carried out on the summary results from the cohort-specific EWAS models for each of the time points (i) to identify at-birth DNAm signals associated with season of birth (winter, spring, or summer with autumn as the reference season) and (ii) to investigate whether such signals persist into childhood. Leave-one-out analyses showed that none of the individual cohorts unduly influenced the meta-analysis results.

**Fig. 2:**
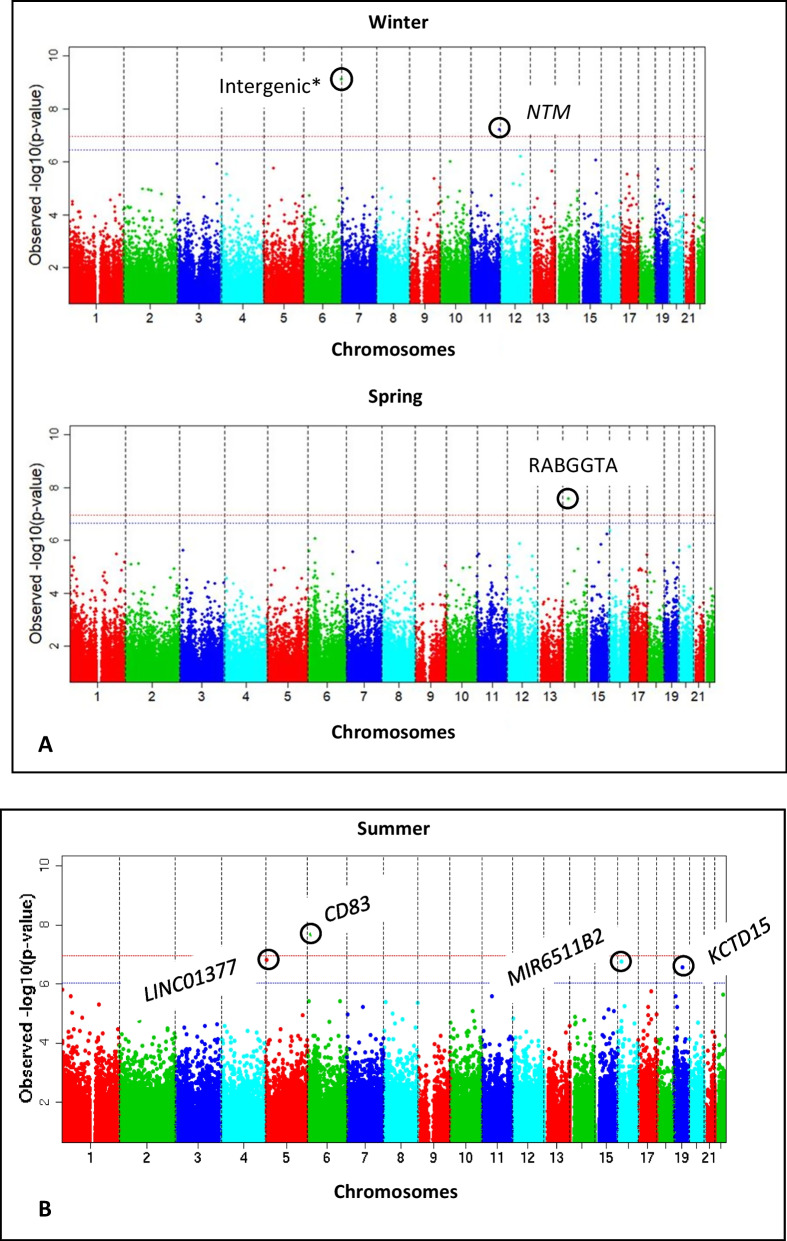
Manhattan plots of the meta-analysed data showing the association of season of birth and DNA methylation at birth using neonatal blood data (**A**) and during childhood using whole blood data (**B**). Birth seasons with FDR-significant CpGs when compared to autumn as the reference season alone are shown in the figure. The blue and red lines indicate the threshold *p* values for false discovery rate (FDR) and the Bonferroni adjustments, respectively. The observed *p* values on the *Y*-axis are from models adjusted for covariates and cell types for the seasons indicated in the figures when compared to autumn as the reference season. Genes associated with the CpGs (circled) are indicated. All cohort-specific EWAS analyses were adjusted for gender of the child, gestational age at delivery, maternal age at delivery, maternal smoking during pregnancy, maternal socio-economic status, batch, child’s age at the time of sample collection (in the case of childhood samples and if data were available) and estimated cell proportions. *This CpG (cg01801443, location: intergenic) has a SNP within 10 base pairs and is not included in Table 1

**Fig. 3:**
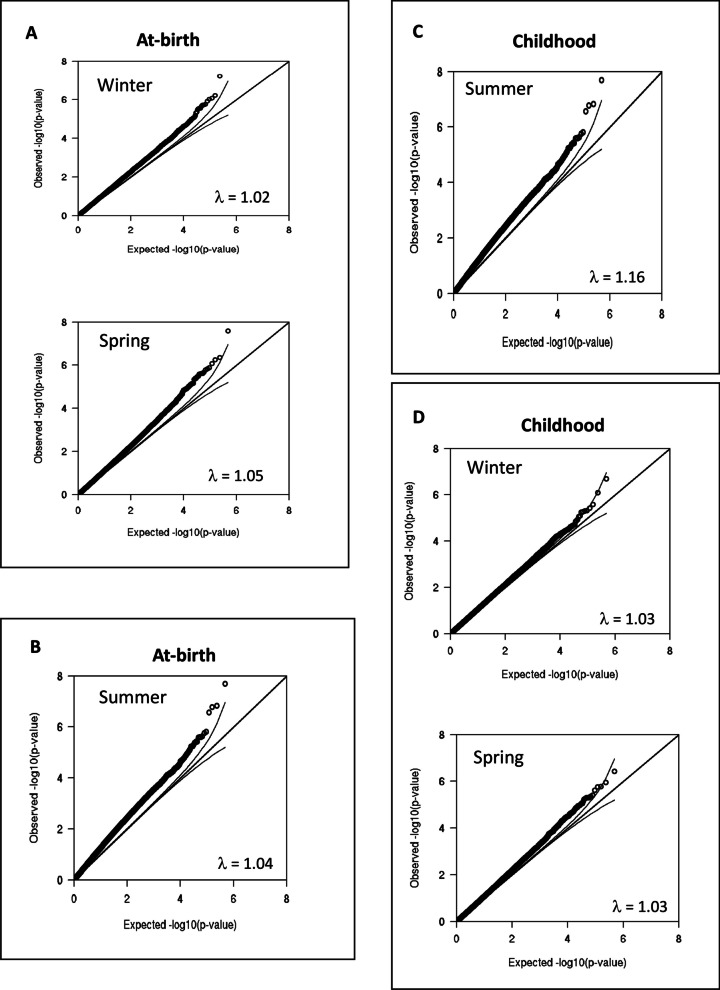
Quantile–quantile (Q-Q) plots for post-meta-analysis models for association between seasons of birth and DNA methylation (primary analyses) for at-birth (**A** and **B**) and childhood (**C** and **D**) samples. The Q–Q plots were generated by plotting observed *p* values (*y*-axis) against the expected uniform distribution of *p* values under the null hypothesis of no association (*x*-axis). Lambda and bias were estimated using BACON method [37]. All cohort-specific EWAS analyses were adjusted for gender of the child, gestational age at delivery, maternal age at delivery, maternal smoking during pregnancy, maternal socio-economic status, batch, child’s age at the time of sample collection and estimated cell proportions

The six CpGs that passed the pre-specified 5% FDR threshold for winter-, spring- and summer-born children for the at-birth and childhood analyses are presented in Table 1 and Figs. 2 and 3. The two FDR significant CpGs identified in the new-borns, cg26416241 (winter-born, mapped to *NTM*) and cg18978324 (spring-born, mapped to *RABGGTA*), had statistical significance that passed the more stringent Bonferroni-corrected threshold of 1.06 × 10^−7^ (CpGs shown in bold, Table 1). The methylation levels of the at-birth CpGs were higher in the winter- and spring-born offspring) when compared to those born in the autumn. This trend was consistent across most of the cohorts (winter: 18; spring: 17 out of a total of 24 cohorts). There was no statistical evidence to suggest that the two CpGs (cg26416241, cg18978324) identified in the at-birth analysis (preliminary analysis) persisted into childhood (Additional file 4: Table S3). In the individual cohort samples of the relevant seasons, these two CpGs mostly had higher methylation levels when compared to the autumn levels in both at-birth samples (75–77% of the cohorts) and childhood samples (58% for both CpGs).

Mean differences in methylation between autumn and each of the other two seasons were small in absolute terms [mean change in DNAm winter vs. autumn (cg26416241): 0.0034 (0.3%) and spring vs. autumn (cg18978324): 0.006 (0.6%)].

In the analysis of childhood samples (aged 1–11 years), differential DNAm was observed at four CpG sites (cg19416462, cg01656588, cg03263237 and cg15437053) in the summer-born children (FDR < 0.05, Table 1) when compared to the autumn-born children. One of the four CpGs, cg03263237, was a cross-reactive probe [46] and was excluded from further biological interpretations. The observed between-study heterogeneity was low to moderate (*I*^2^ < 50) for the FDR-significant CpGs. One of the four CpGs, cg19416462 (mapped to *CD83*), also passed the Bonferroni-corrected threshold *p* value. The remaining CpGs, after excluding the cross-reactive probe of the four childhood CpGs, two showed decreased methylation levels in children born in the summer compared to those born in the autumn in 92–100% of the cohorts, while cg15437053 was more methylated than the same CpG in autumn born children in 57% of the cohorts. The corresponding three CpGs in the at-birth samples of summer-born infants lacked even nominal significance but showed an overall tendency towards hypomethylation compared to the autumn-born infants (57–61% of the contributing cohorts). The mean differences in methylation (childhood analysis: summer vs. autumn) were small in absolute terms [mean change in DNAm (cg19416462): − 0.4%, (cg1656588): − 0.7%, (cg15437053): 0.4%].

### Differentially methylated regions (DMRs) at birth and in childhood associated with season of birth

Several DMRs, containing at least two CpG sites, were associated with season of birth both in new-borns and in children aged 1–11 years (Table 2). There were 24 DMRs mapping to 27 known genes in the at-birth analysis. CpGs of the DMRs were mostly hyper-methylated, and their direction in the DMRs was the same in most cohorts (Additional file 5: Table S4). The DMRs identified and the genes mapped to these differential methylation signals were unique to the seasons apart from two DMRs mapped to *HLA-F* (in both winter and spring) and to *AZU1* (in spring and summer, Table 2). The childhood analysis identified 32 DMRs mapped to 35 genes. CpGs of the DMRs were mostly hypo-methylated, and their direction in the DMRs was the same in most cohorts (Additional file 5: Table S4). Like in the at-birth analyses, the season of birth associated DMRs of childhood samples was unique to the season of birth except for two mapped genes, *LOC441666* in the winter- and summer-born and *HTR2A* in winter- and spring-born children. There was no overlap between the at-birth and childhood DMRs.

**Table 2:** Differentially methylated regions (DMRs) at birth and in childhood in different seasons

Season of birth^a^	CHR	Region	CpGs in the region	Šidák *p* value^b^	Minimum *p* value^c^	Gene (hg19)	Gene group
*At-birth DMRs*							
Winter	7	1753371–1753934	7	1.7 × 10^−6^	9.4 × 10^−7^	*ELFN1*	intron, 5′UTR
	16	8806334–8807068	13	2.5 × 10^−4^	3.9 × 10^−4^	*ABAT*	TSS, exon, 5′UTR, intron
	1	59042906–59043601	10	6.9 × 10^−4^	3.6 × 10^−4^	*TACSTD2*	TSS, exon
	6	29690797–29692424	17	1.5 × 10^−3^	3.6 × 10^−4^	*HLA-F*	TSS, intron, 5′UTR, cds
	11	2907309–2907955	9	3.0 × 10^−3^	1.0 × 10^−3^	*CDKN1C*	Intergenic
	7	1095391–1095745	6	4.1 × 10^−3^	1.0 × 10^−3^	*GPR146*, *C7orf50*	intron, 5′UTR, intron, intron, exon, 5′UTR
	4	206087–206587	8	4.3 × 10^−3^	3.6 × 10^−4^	*ZNF876P*	nc_intron, nc_exon
	21	15134466–15135068	3	7.5 × 10^−3^	1.8 × 10^−3^	*LOC110091777*	nc_exon
Spring	11	368326–369217	24	2.9 × 10^−8^	2.6 × 10^−9^	*B4GALNT4*	intergenic
	6	31148307–31148773	16	3.2 × 10^−7^	1.4 × 10^−7^	*PSORS1C3*	nc_intron
	6	29690741–29692607	27	5.8 × 10^−6^	6.4 × 10^−7^	*HLA-F*	TSS, intron, 5′UTR, cds
	6	41068528–41069073	8	5.9 × 10^−6^	5.8 × 10^−7^	*ADCY10P1*, *NFYA*	nc_intron, nc_exon, exon, 3′UTR
	12	132976697–132977165	4	1.2 × 10^−4^	2.4 × 10^−5^	*LOC101928416*	intergenic
	17	17603506–17604209	6	1.6 × 10^−4^	3.9 × 10^−5^	*RAI1*	intron, 5′UTR
	19	827690–828195	6	2.3 × 10^−4^	4.4 × 10^−5^	*AZU1*	TSS, intron, 5′UTR, cds
	11	60534886–60535091	4	4.3 × 10^−4^	4.0 × 10^−5^	*MS4A15*	nc_intron, nc_exon, intron, cds, 5′UTR
	19	11784489–11785150	10	4.7 × 10^−4^	8.4 × 10^−7^	*ZNF833P*	nc_intron, nc_exon
	12	6876163–6876346	2	1.3 × 10^−3^	9.3 × 10^−5^	*PTMS*	Intron
	11	1319623–1320659	6	1.2 × 10^−3^	1.3 × 10^−2^	*TOLLIP*	intron, 5′UTR
	20	35402098–35402548	13	4.2 × 10^−3^	2.5 × 10^−4^	*DSN1*	TSS, exon, 5′UTR
	2	239139886–239140215	6	4.8 × 10^−3^	4.8 × 10^−4^	*LINC02610*	nc_intron, nc_exon
	11	2721182–2721657	15	6.8 × 10^−3^	1. 8 × 10^−4^	*KCNQ1*, *KCNQ1OT1*	intron, nc_exon
	11	47399788–47400355	9	7.6 × 10^−3^	8.1 × 10^−4^	*SPI1*	TSS, intron, 5′UTR, cds
Summer	19	827690–828195	6	3.8 × 10^−4^	1.1 × 10^−3^	*AZU1*	TSS, intron, 5′UTR, cds
	7	43288610–43288914	4	9.3 × 10^−4^	1.1 × 10^−3^	*HECW1*	Intron
	13	20392381–20392731	5	8.5 × 10^−3^	4.7 × 10^−3^	*ZMYM5*	intergenic
*Childhood DMRs*							
Winter	1	153599454–153600181	8	9.6 × 10^−13^	9.5 × 10^−11^	*S100A13*	TSS, exon, 5′UTR, introns
	19	55476640–55477835	7	3.8 × 10^−8^	1.2 × 10^−3^	*NLRP2*	TSS, intron, exon, 5′UTR, nc_intron, nc_exon
	13	36871621–36872371	14	4.0 × 10^−8^	4.2 × 10^−7^	*CCDC169*, *CCDC169-SOHLH2*	TSS, intron, 5′UTR, cds, exon
	5	191461–192128	9	2.6 × 10^−5^	1.5 × 10^−5^	*LRRC14B*	TSS, 5′UTR, cds
	11	67383352–67384065	8	9.0 × 10^−5^	4.9 × 10^−5^	*DOC2GP*	Intergenic
	16	31159533–31159945	5	3.5 × 10^−4^	1.1 × 10^−4^	*PRSS36*	intron, cds
	10	42862851–42863533	6	4.6 × 10^−4^	1.8 × 10^−3^	*LOC441666*	nc_intron, nc_exon
	13	47472025–47472454	12	1.7 × 10^−3^	3.7 × 10^−4^	*HTR2A*	Intergenic
	13	113540164–113540656	6	2.9 × 10^−3^	6.0 × 10^−4^	*ATP11A*	exon, 3′UTR
	10	43447046–43447643	3	6.6 × 10^−3^	1.2 × 10^−3^	*LINC01264*	Intergenic
	1	1003101–1003554	4	6.7 × 10^−3^	1.0 × 10^−3^	*LOC105378948*	nc_intron, nc_exons
	1	2121014–2121546	4	7.7 × 10^−3^	1.1 × 10^−3^	*FAAP20*, *LOC112268219*	nc_intron, nc_exon, intron, exon, 5′UTR, 3′UTR, cds
Spring	1	230415160–230415693	6	5.3 × 10^−9^	2.5 × 10^−8^	*GALNT2*	3′UTR, cds
	17	33759459–33760318	11	1.6 × 10^−7^	1.0 × 10^−5^	*SLFN12*	TSS, exon, 5′UTR, intron
	13	47472025–47472454	12	2.1 × 10^−5^	1.0 × 10^−5^	*HTR2A*	Intergenic
	3	193922012–193922718	7	6.9 × 10^−5^	3.7 × 10^−5^	*LINC02036*	nc_intron, nc_exon
	6	163148828–163149478	6	2.5 × 10^−4^	1.0 × 10^−5^	*PACRG*	intron, 5′UTR, cds, TSS
	6	31543194–31543711	11	2.1 × 10^−3^	1.3 × 10^−3^	*TNF*	TSS, intron, 5′UTR, cds
	8	1094459–1094929	6	4.7 × 10^−3^	9.8 × 10^−4^	*DLGAP2*	Intron
	11	10715150–10715385	6	4.9 × 10^−3^	2.2 × 10^−3^	*IRAG1*	TSS, exon, 5′UTR
	19	39737743–39737886	3	8.6 × 10^−3^	6.7 × 10^−4^	*IFNL4*	intron, 3′UTR, cds, nc_intron, nc_exon
	19	10735981–10736380	7	9.5 × 10^−3^	1.4 × 10^−3^	*SLC44A2*	TSS, intron, 5′UTR, cds
Summer	10	123355243–123356361	9	3.0 × 10^−8^	3.9 × 10^−7^	*FGFR2*	nc_intron, TSS, intron, exon, 5′UTR
	7	27183108–27184878	47	5.0 × 10^−7^	2.1 × 10^−6^	*HOXA-AS3*, *HOXA3*, *HOXA5*	nc_intron, TSS, 5′UTR, cds
	6	41394131–41395119	5	1.1 × 10^−5^	1.2 × 10^−5^	*LINC01276*	intergenic
	17	21280992–21281532	5	2.0 × 10^−5^	1.3 × 10^−5^	*KCNJ12*	intron, 5′UTR
	10	131697055–131698005	7	8.1 × 10^−4^	2.4 × 10^−4^	*EBF3*	Intron
	1	247171209–247171863	10	1.0 × 10^−3^	2.4 × 10^−4^	*ZNF695*, *ZNF670-ZNF695*	TSS, 5′UTR, cds, nc_intron, nc_exon
	5	2334377–2334996	5	1.7 × 10^−3^	1.4 × 10^−4^	*LOC100506858*	intergenic
	10	42862851–42863619	9	1.7 × 10^−3^	3.7 × 10^−4^	*LOC441666*	nc_intron, nc_exon
	7	148768516–148769245	3	4.4 × 10^−3^	8.0 × 10^−4^	*ZNF786*	cds
	17	48278753–48279290	9	9.3 × 10^−3^	1.1 × 10^−3^	*COL1A1*	TSS, intron, 5′UTR, cds

Differentially methylated regions (DMRs) were identified using comb-p. The inputs for comb-p were the annotated meta-analysed outputs of at-birth and childhood blood EWASs

*CHR* chromosomes, *UTR* untranslated region, *TSS* transcription start site, *cds* coding sequence

^a^Reference season in EWAS of individual cohorts: autumn

^b^Regions with spatially adjusted and Šidák multiple testing corrected *p* value < 0.05

^c^*P* value for the most significant CpG in a DMR identified by comb-p

### DNA methylation at birth and in childhood by latitude

We performed additional at-birth and childhood meta-analyses in subgroups stratified by latitude to check if the observed temporal associations varied with latitude. Cohorts, all from the northern hemisphere, were divided into two latitude groups—higher latitude (≥ 50°N, 9 cohorts, *N* = 5580, 59.3%) and the lower latitude [37.2–50°N (12 cohorts, *N* = 3823, 40.7%)].

In the at-birth analyses, we identified a single CpG, cg06251958, from the spring-born babies of the higher latitude subgroup (FDR-adjusted *p* value = 0.001, Table 3) and none from the winter- or summer-borns (all against autumn). This CpG (mapped to *PSMC2*) also passed the Bonferroni *p* value threshold (1.06 × 10^−7^). There were seven significant (FDR *p* value < 0.05) at-birth CpGs, six in the spring-born and one in the winter-born infants in the lower latitude subgroup analyses. One of these seven CpGs, cg23369114, was a cross-reactive probe [46]. There was no overlap in the FDR-adjusted differentially methylated CpGs between the seasons in each latitude subgroup nor between the two latitude subgroups (higher vs. lower) in any given birth season. The characteristics of cohorts from the higher and lower latitudes were similar apart from the latitude of the place of birth (Additional file 1: Table S1B). These results, therefore, suggest that the temporal associations between season of birth and DNAm were also latitude specific.

**Table 3:** Association between season of birth and DNA methylation in at-birth and childhood stratified by latitude

Season of birth^a^	CpG	Coeff	SE	*p* value	FDR *p* value	Lambda^b^	CHR	Position	Mapped gene	Direction^c^	*I*^2^
*At-birth*											
Higher latitude subgroup (≥ *50°N)*											
Spring	cg06251958	0.0095	0.0016	**2.0** × **10**^**−9**^	0.001	1.01	7	102989081	PSMC2	+ + ? + + + − − +	78
Lower latitude subgroup (< *50°N)*											
Spring	cg01113988	− 0.0082	0.0015	**7.13** × **10**^**−8**^	0.028	1.06	6	106035528	PREP	− − − + − − − − − − − +	29.4
	cg22168386	− 0.0103	0.002	1.34 × 10^−7^	0.028		13	24205538	TNFRSF19	− − − − + + ? − + − − −	40.1
	cg17462107	0.003	0.0006	2.21 × 10^−7^	0.028		5	66199867	MAST4	+ − + + ? + + + + + + −	14.9
	cg23369114^d^	− 0.0086	0.0017	3.83 × 10^−7^	0.031		19	14198698	C19orf67	− ? − − + − − ? − − − −	21.2
	cg11520554	0.0022	0.0004	4.14 × 10^−7^	0.031		15	80216034	C15orf37; ST20	+ + + + + + + − + + − −	70.3
	cg20954180	0.0052	0.001	4.64 × 10^−7^	0.031		3	54606265	CACNA2D3	+ + + + + + + + + + + −	23.6
Winter	cg01801443	− 0.0045	0.0007	**1.65** × **10**^**−10**^	7.77 × 10^−5^	1.02	6	168629778	LOC105378137	+ − − − + − ? − − − − ? − −	40.5
*Childhood*											
Higher latitude subgroup (≥ *50°N)*											
Winter	cg22709217	0.0129	0.0024	**6.97** × **10**^**−8**^	0.021	1.12	22	50311962	ALG12; CRELD2	+ + + + + + + +	36.2
	cg18124955	0.0013	0.0002	**1.09** × **10**^**−7**^	0.021		2	173292329	ITGA6	+ + + + + + + +	0

Significant CpGs identified in meta-analyses of EWAS summary results from 9 (≥ 50°N, *N* = 5580) and 12 (< 50°N, *N* = 3823) cohorts for the at-birth latitude analyses. The *p* values for the CpGs that passed the Bonferroni-corrected significance threshold (1.06 × 10^−7^) are shown in bold. Cohort-specific EWAS models were adjusted for sex of the child, gestational age and maternal age at delivery, maternal smoking during pregnancy, maternal socio-economic status, batch, and cell type proportions

Coeff: regression coefficient (change in mean methylation compared to autumn reference); SE: standard error of the coefficient; Lambda: genomic inflation factor; CHR: chromosome; *I*^2^: a measure of between-study variability

^a^Reference season: autumn

^b^Estimated using the BACON method of van Iterson et al. Ref [37]:

^c^ + : hyper-methylation; –: hypo-methylation

^d^Cross-reactive probe (Chen et al., Ref: [46])

The childhood analyses of the higher latitude cohorts revealed two FDR-significant (also Bonferroni-significant) CpGs in the winter-born children and none in the spring- and summer-born. Furthermore, there was no overlap in CpG methylation signals between the at-birth and childhood samples. We did not perform childhood DNAm analysis for the lower latitude subgroup due to its small sample size (*N* = 936, Fig. 1).

### Differentially methylated regions (DMRs) at birth and in childhood by latitude

Interrogation of at-birth DMRs of the higher latitude subgroup (≥ 50°N) identified 18 DMRs mapped to 20 unique genes in the winter-born and 25 DMRs (23 mapped genes) in the summer-born infants (Table 4). In contrast, there were relatively fewer DMRs (*n* = 7) in the spring-born infants of the higher latitude cohorts. Most of the at-birth DMRs identified in the new-borns were unique to the season of birth (winter, spring, or summer) except for DMRs which mapped to *ELFN1*, *SEMA5B* and *LOC154449* (identified in winter- and summer-born children) and *AZU1* (in spring- and summer-born).

**Table 4:** Season of birth associated differentially methylated regions (DMRs) at birth in cohorts from latitudes ≥ 50°N

Season of birth^a^	CHR	Region (hg19)	CpGs in the region	Šidák *p* value^b^	Minimum *p* value^c^	Gene	Gene group
Winter	1	203733946–203734584	6	7.2 × 10^−8^	1.7 × 10^−7^	*LAX1*	TSS, intron, 5′UTR, cds; exon
	6	170571118–170572041	10	1.1 × 10^−5^	1.1 × 10^−6^	*LOC154449*	nc_intron, nc_exon
	7	1753371–1753934	7	1.4 × 10^−5^	9.0 × 10^−6^	*ELFN1*	intron, 5′UTR
	3	122712113–122712742	4	3.7 × 10^−5^	2.1 × 10^−5^	*SEMA5B*	TSS, exon, 5′UTR; intron, nc_intron
	19	35629676–35630499	10	4.2 × 10^−5^	2.1 × 10^−5^	*FXYD1*	TSS, intron, exon, 5′UTR
	17	72442903–72443426	6	1.1 × 10^−4^	3.5 × 10^−5^	*GPRC5C*	intron, 3′UTR, cds
	11	299365–300797	12	1.8 × 10^−4^	5.3 × 10^−3^	*IFITM5*	TSS, 5′UTR, cds
	1	223316194–223317573	13	2.4 × 10^−4^	5.3 × 10^−3^	*TLR5*	TSS, intron, exon, 5′UTR
	20	61583512–61584184	11	1.1 × 10^−3^	2.5 × 10^−4^	*SLC17A9*	TSS, 5′UTR, cds; exon
	8	39171595–39172145	9	2.6 × 10^−3^	4.2 × 10^−4^	*ADAM5*	intergenic
	14	24780142–24780951	11	3.3 × 10^−3^	2.1 × 10^−4^	*LTB4R2; CIDEB; LTB4R*	3′UTR, cds; TSS, exon, 5′UTR; exon
	15	45021092–45021786	3	3.4 × 10^−3^	6.7 × 10^−4^	*LOC100419583*	nc_intron, nc_exon
	14	96180294–96181069	11	4.5 × 10^−3^	6.7 × 10^−4^	*TCL1A*	nc_exon; TSS, 5′UTR, cds
	4	122853439–122854430	9	4.7 × 10^−3^	7.7 × 10^−4^	*TRPC3*	intron, cds; TSS, 5′UTR
	21	15077071–15077799	3	5.5 × 10^−3^	8.1 × 10^−4^	*LOC110091777*	intergenic
	3	122640753–122641341	6	8.3 × 10^−3^	1.1 × 10^−3^	*SEMA5B*	nc_intron, nc_exon; intron, cds
	20	42543009–42543803	9	9.4 × 10^−3^	7.3 × 10^−4^	*TOX2*	TSS, intron, 5′UTR, cds
	3	46759071–46759723	9	0.00998	1.3 × 10^−3^	*PRSS50*	TSS, intron, 5′UTR, cds
Spring	6	28058690–28059233	10	1.2 × 10^−5^	3.0 × 10^−5^	*ZSCAN12P1*	nc_exon
	19	827690–828195	6	2.9 × 10^−4^	1.8 × 10^−4^	*AZU1*	TSS, intron, 5′UTR, cds
	11	368326–368939	16	6.5 × 10^−4^	2.5 × 10^−4^	*B4GALNT4*	intergenic
	6	24646448–24646807	7	1.3 × 10^−3^	3.9 × 10^−4^	*KIAA0319*	intergenic
	6	29648136–29648926	22	1.5 × 10^−3^	2.5 × 10^−4^	*ZFP57*	intron, exon, 5′UTR
	6	31148307–31148773	16	4.1 × 10^−3^	9.0 × 10^−4^	*PSORS1C3*	nc_intron
	6	29893901–29894366	16	6.0 × 10^−3^	5.9 × 10^−5^	*HCG4B*	nc_exon
Summer	7	1733107–1734350	9	4.0 × 10^−6^	8.9 × 10^−5^	*ELFN1*	intron, 5′UTR
	20	57425954–57427846	62	4.8 × 10^−5^	1.2 × 10^−4^	*GNAS*, *GNAS-AS1*	intron, 5′UTR, nc_exon, 3′UTR
	7	50628943–50630108	7	1.3 × 10^−4^	2.0 × 10^−4^	*DDC*	intron, 5′UTR
	19	827404–828195	8	1.7 × 10^−4^	8.9 × 10^−5^	*AZU1*	TSS, intron, 5′UTR, cds
	10	134664446–134665203	6	2.5 × 10^−4^	2.0 × 10^−4^	*CFAP46*	intron, cds
	14	101290531–101293115	26	2.8 × 10^−4^	2.0 × 10^−4^	*MEG3*	nc_intron, nc_exon
	12	132973282–132974687	8	3.5 × 10^−4^	2.0 × 10^−4^	*LOC101928416*	intergenic
	6	32046638–32049288	15	3.7 × 10^−4^	3.0 × 10^−3^	*TNXB*	intron, cds
	12	133414230–133415291	6	5.1 × 10^−4^	3.7 × 10^−3^	*CHFR*	exon, 3′UTR
	6	28226860–28227507	11	1.3 × 10^−3^	3.8 × 10^−4^	*NKAPL; ZKSCAN4*	TSS, cds, intron, exon, 5′UTR
	6	32051832–32054119	23	2.1 × 10^−3^	7.9 × 10^−3^	*TNXB*	intron, cds
	6	32039771–32041859	20	2.9 × 10^−3^	5.5 × 10^−3^	*TNXB*	intron, cds
	19	5048401–5049160	4	3.6 × 10^−3^	6.8 × 10^−4^	*KDM4B*	intron
	1	2387645–2388098	5	4.2 × 10^−3^	6.0 × 10^−4^	*PLCH2*	intergenic
	7	24323236–24324460	10	4.3 × 10^−3^	7.3 × 10^−4^	*NPY*	TSS, intron, 5′UTR
	7	130131233–130132478	37	4.6 × 10^−3^	6.8 × 10^−4^	*MEST*	TSS, intron, 5′UTR, cds, exon
	7	1753371–1753934	7	5.0 × 10^−3^	6.8 × 10^−4^	*ELFN1*	intron, 5′UTR
	2	27664992–27665736	11	5.6 × 10^−3^	7.1 × 10^−4^	*NRBP1*, *KRTCAP3*	exon, 3′UTR, TSS, intron, 5′UTR, cds
	3	122712113–122712922	5	5.7 × 10^−3^	7.1 × 10^−4^	*SEMA5B*	TSS, exon, 5′UTR; intron, nc_intron
	12	53183483–53184111	6	5.9 × 10^−3^	5.1 × 10^−4^	*KRT3*	3′UTR, cds
	6	170571118–170572041	10	6.0 × 10^−3^	4.3 × 10^−4^	*LOC154449*	nc_intron, nc_exon
	20	57414014–57415202	15	6.5 × 10^−3^	7.6 × 10^−4^	*GNAS-AS1*, *GNAS*	nc_intron, TSS, 5′UTR, cds
	10	35893599–35894455	9	6.6 × 10^−3^	3.6 × 10^−4^	*GJD4*	TSS, exon, 5′UTR
	7	57484128–57484846	6	8.7 × 10^−3^	7.9 × 10^−4^	*MIR3147*	intergenic
	6	32043004–32043764	5	8.8 × 10^−3^	4.1 × 10^−4^	*TNXB*	intron

Differentially methylated regions (DMRs) were identified using comb-p. The inputs for comb-p were the annotated meta-analysed outputs of at-birth EWASs

*CHR* chromosomes, *UTR* untranslated region, *TSS* transcription start site, *cds* coding sequence

^a^Reference season in EWAS of individual cohorts: autumn

^b^Regions with Šidák *p* value < 0.05

^c^*P* value for the most significant CpG in a DMR identified by comb-p

Examination of the biological functions of the genes mapped to the at-birth higher latitude DMRs revealed that most of the genes have roles, amongst others, in the functions of the central nervous system (CNS) including psychological disorders, cancer or inflammation and immunity (see Additional file 6: Table S5, for a more detailed, but not exhaustive, list of genes associated with at-birth DMRs and their functions). The DMRs identified in the new-borns from the lower latitude (< 50°N) cohorts were also unique to seasons of birth (Additional file 7: Table S6). None of the DMRs identified in the higher latitude subgroup were present in the lower latitude samples (compare Table 4 and Additional 7: Table S6).

Interrogation of childhood DMRs of the higher latitude subgroup (≥ 50°N) identified 47 DMRs which were mapped to 54 genes (winter: 21 DMRs and 24 genes, spring: 16 DMRs and 18 genes, summer: 10 DMRs and 12 genes, Additional file 7: Table S7). Most of these DMRs were unique to the season of birth (winter, spring, or summer) except for a DMR that mapped to *S100A13* identified in both winter- and spring-born children and a second DMR that mapped to *MIR7159* identified in both winter- and summer-born children. Furthermore, there was little overlap between the DMRs identified in the at-birth (Table 4) and childhood analyses of the higher latitude subgroup (Additional file 7: Table S7).

Four at-birth DMRs of the summer-born babies from the higher latitude subset mapped to imprinted genes, *GNAS*/*GNAS-AS1*, *MEG3* and *MEST*, with two of them mapping to the *GNAS* locus (*GNAS* and *GNAS-AS1*). These imprinted genes were specific for the summer-born babies and were not identified in the new-borns of spring or winter (Table 4). Whilst DMRs associated with *GNAS*/*GNAS-AS1* and *MEG3* were present only in the at-birth samples from the higher latitude subgroup, a DMR associated with the *MEST* gene (mesoderm-specific transcript) was present in both at-birth and in early childhood from the higher latitude subgroup (compare Table 4 with Additional file 7: Table S7.

### Trait enrichment analysis

Trait Enrichment analysis was carried out on CpGs with unadjusted meta-analysed association *p* value < 1.0 × 10^5^ using EWAS Atlas [42]. This analysis revealed that, apart from enrichment for traits related to cancers or CNS-associated disorders, the enriched traits are mutually exclusive between at-birth and childhood analyses (Additional file 9). The traits with higher odds ratios for enrichment in the at-birth analysis were vitamin B12 supplements, polycystic ovary syndrome, maternal stress, systemic lupus erythematosus, and Behcet’s disease. These traits were enriched in the at-birth but not in the childhood samples and were mostly in new-borns of mothers with the early months of their pregnancy in the months of autumn to winter, which may indicate influence of exposures in the earlier parts of foetal development.

A similar trend of mutual exclusivity of enriched traits was seen when the new-borns of higher (≥ 50°N) and lower (< 50°N) latitudes were compared. While the higher latitude new-borns alone had traits enriched in exposure to pollutants (particulate matter, nitric oxide, and maternal arsenic exposure) and folic acid supplement, the lower latitude offspring had unique enriched traits associated with response to different treatments, mixed connective tissue disease, asthma, puberty, and household socio-economic status. Parental arsenic exposure and autistic spectrum disorder were common in the new-borns and young children of higher latitude, although weakly enriched in the childhood analysis.

### Metastable epialleles

We tested CpGs with unadjusted *p* value < 0.05 in meta-analyses for enrichment of metastable epialleles (loci whose epigenetic modifications are established during early embryonic development [47]). Our analyses found no evidence of enrichment of metastable epialleles.

## Discussion

This study investigated the association of season of birth and DNAm at two time points (at-birth and in childhood), and in latitude subgroups, in multiple cohorts recruited from regions across the northern hemisphere. Season of birth can be a proxy for seasonal variations in the length of the day, temperature, availability of sunlight, exposure to UV light, pollen, nutrition, seasonal infectious agents, air pollution, C-section and many more. Here, we show evidence for the existence of season of birth specific associations with DNAm at-birth as well as in childhood. The differential methylation patterns in at-birth samples followed a season-dependent annual periodicity (temporal effect). In addition, we found suggestive evidence for latitude-specific fluctuations in DMRs (a spatial effect). To the best of our knowledge, our study is a first to demonstrate such season of birth effects on DNAm as well as the latitude specificity in these effects in a large study population with participants from diverse geographical locations.

### Season of birth specific differential DNA methylation

Our primary analyses found epigenome-wide significant season of birth associations at two and four CpGs in the at-birth and childhood blood samples, respectively. One of the four CpGs in the childhood samples was found to be a cross-reactive probe [46] and should be interpreted with caution. The remaining differentially methylated CpGs were unique for the birth seasons in the at-birth and childhood samples. Furthermore, there was no overlap in the CpG methylation signals observed across the two time points investigated (at-birth and childhood). Several DMRs associated with the birth seasons were identified in the at-birth and childhood analyses. CpG sites that are close to each other are known to be co-methylated, and together these sites can function as a regional unit [48]. At the individual CpG level, the exposure-induced changes in methylation of these CpGs are often small and with weak statistical evidence. However, analysis of pre-defined regions as functional units, each containing several co-methylated CpGs, may provide more statistical power to detect the associations with methylation signals. Half of the DMRs identified in this study were in gene locations such as coding sequences, transcriptional start sites and/or untranslated 5′ or 3′ ends. The DMRs identified were in the vicinity of genes with known functions, amongst others, in tumorigenesis, psychiatric/neurological disorders or inflammation and immunity. Most DMRs and their mapped genes in the at-birth (24 out of 26) and childhood (30 out of 32) DMRs were specific to the birth seasons. The seasonal specificity of DMRs was consistent with the observed temporal effect of DNAm signals at the CpG level both at birth and in childhood. Like in the case of differentially methylated season-of-birth-associated CpGs, there was little overlap between most of the at-birth and childhood DMRs identified which may indicate that the at-birth methylation signals associated with season of birth did not persist over time.

### Association between season of birth and DNA methylation is latitude dependent

Latitude-stratified analyses (higher latitude: ≥ 50°N and lower latitude: < 50°N) were carried out to explore the impact of latitude on seasonal differences in at-birth and childhood DNAm. One of the significant CpGs of the at-birth lower latitude analysis, cg23369114 (Table 3) was a cross-reactive probe identified by Chen et al. [46] which warrants caution while interpreting the results. Like in the primary analyses, temporal associations (season-specific) were observed in the two latitude subgroups for the at-birth and the higher latitude childhood analyses both at the CpG (6/7 CpGs) and DMR levels. Furthermore, the DMRs of at-birth and childhood analyses of the higher latitude subgroup were mutually exclusive except for two DMRs which were present at both time points. This is consistent with the findings in the primary analysis that majority of the at-birth associations between season of birth and methylation signals did not persist during childhood. Like in the primary analyses, the observed associations between season of birth and DNAm signals (CpG/DMR) were unique for the latitudes in each of the seasons. These findings point to a latitude-dependent spatial effect of the association between DNAm and season of birth.

The absence of sustained signals from birth to childhood in this study does not necessarily imply absence of persistence in general. There could be several reasons for this. The smaller sample size of the childhood analysis compared to that of the at-birth analysis might be one of the reasons. Choice of cord blood for the at-birth studies could have masked the birth to childhood persistence of DNAm signals in this study. For example, Paquette and Marsit highlighted the importance of placenta samples for studies on the influence of intrauterine *HTR2A* gene expression on early childhood and/or lifelong health outcomes [49]. The current study identified a DMR on chromosome 13 (47472025–47472454) in the winter-born children of the primary and the higher latitude subgroup childhood analyses. The same DMR, mapped to *HTR2A* (codes for serotonin receptor), was present in the spring-born children of the primary childhood analysis, but not in any of the at-birth analyses that were based mostly on cord blood data. Yet another factor could be the absence of paired comparisons in our study. The at-birth and childhood comparisons were done with > 80% of unpaired data as most cohorts contributed to either at-birth or childhood data. This meant that DNAm measurements at the two time points did not come from the same child. This might have made the true persistent signals, if there were any, undetectable. An analysis with paired data from the six cohorts (data available from the same child at both the time points) would have had limited power to detect methylation signals of small DMR sizes as such data were available for < 20% of the study population. The heterogeneity of the age group in the childhood analysis may also have contributed to the non-persistence of the at-birth DNAm signals. Age effects may be strong, and the age range was wide (1–11 years). It is likely that there may be some remaining effect despite controlling for child’s age in the models.

It may be noted that persistence of DNAm signals from birth to childhood is not necessarily needed for differential methylation to have an effect. Differential methylation at birth, which affects developmental programmes at a critical period, may have long-lasting effects, even if the differential methylation does not persist into early childhood.

The season-related DNAm signals (CpGs and DMRs) identified in the primary and latitude analyses were non-overlapping. The primary analysis investigated the seasonal variations disregarding the information on the latitude of birth of the children. The results make use of the distribution of methylation signals across the seasons only. In contrast, the latitude analysis is a stratified analysis which examines the above seasonal variations in two separate latitude regions (lower and higher latitudes in the northern hemisphere). The results of such an analysis look for differences in the distribution of methylation signals across the seasons in two separate latitude subgroups which are likely to be different from that of the primary analysis. However, the DMR mapped to HTR2A was present both in the winter- and spring-born children of the preliminary childhood analysis as well as the winter-born children of the higher latitude childhood analysis. Alternatively, the non-overlapping methylation signals observed in the latitude subgroups, despite smaller sample sizes and stringent FDR cut offs, are likely to be the enriched latitude-specific signals which were not visible in the primary analysis.

### Genes implicated in the season of birth association of DNA methylation

Several of the differentially methylated probes (DMPs) and DMRs identified in our analyses mapped to genes with well characterised functions. We highlight below some of the genes mapped to DMPs or DMRs identified in the at-birth and childhood analyses of the higher latitude subgroup which are likely to be associated with outcomes relevant to season of birth.

The most studied link between season of birth and a disease outcome is that of schizophrenia (SZ). In the general population in the northern hemisphere, individuals born in late winter and early spring have an increased risk of developing SZ later in life [9, 50, 51]. In addition, the prevalence of SZ has also been shown to increase with increasing latitude with the highest prevalence rates at the poles [52, 53]. Correlation patterns of risk factors and SZ identified pre-natal vitamin D deficiency and infections like influenza and toxoplasmosis which are more prevalent in higher latitude and cold climates, as major risk factors of SZ. Interestingly, DNAm of cg12022621, mapped to the *LAX1* gene (Lymphocyte Transmembrane Adaptor 1), has been shown to be associated with severity of certain symptoms of schizophrenia (SZ) in a case–control study [54]. *LAX1* is also the mapped gene for one of the top-ranked at-birth DMRs (Šidák-corrected *p* value: 7.2 × 10^−8^) spanning six CpGs including cg12022621 (crude *p* value: 3 × 10^−4^, Table 4) and was identified only in the winter-born group of the higher latitude subset in the at-birth samples. While *LAX1*-associated SZ was absent from the childhood DMR analyses (Table 4), published literature reveals SZ to be one of the most highlighted outcomes of the major depressive disorder (MDD) family of mental health outcomes in winter- and spring-born children (Additional file 6: Table S5 and references therein). The corresponding associated genes in the childhood analyses were *LY6G5C*, *HTR2A*, *SHANK 1* and *NKAPL*, in the winter-born children and, *SNTG2* in the spring-borns [55–59].

Other genes associated with neurocognitive disorders, neural development, or the central nervous system, not exclusively, in the at-birth analyses of the higher latitude include *ELF1N*, *SEMA5B*, *GPRC5C*, *SLC17A9*, *TOX2*, *PRSS50*, *AZU1*, *KIAA0319*, *PSORS1C3*, *GNAS*, *GNAS*-AS1, *DDC*, *CFAP46*, *MEG3*, *TNXB*, *ZKSCAN4*, *PLCH2*, *NPY*, *NRBP1*, *LTB4R* and *LTB4R2* in the winter-born babies (Additional file 6: Table S5 and references therein). The same in the childhood analyses (higher latitude) was S100A13, *MIR-647*, *ZKSCAN4*, *NAV2*, *NCK2* and *CAT* in the winter-born children, *S100A13*, *SZT2*, *C11orf21*, *PACRG*, *DLEU7* and *TAPBP* in the spring-borns, and *FARS2*, *CHKB* and *RPH3AL* in the summer-born children (see references in Additional file 8: Table S8).

Of note, *SLC17A9*, mapped by a DMR unique to the at-birth analyses of infants of the higher latitude subgroup born in winter (Additional file 6: Table S5), is associated with a skin-specific autoinflammatory disease, disseminated superficial actinic porokeratosis (DSAP) [60]. Induction and exacerbation of DSAP are known to be linked to exposure to sunlight or artificial ultraviolet radiation [61]. Similarly, other genes linked to skin disorders include *PSORS1C3* (spring-born), and *LTB4R* (winter-born), both of which are known to be linked to the autoimmune disease psoriasis [62–64]. Interestingly, prevalence of psoriasis is higher in populations of higher latitude regions of northern Europe [65]. The DMR-associated genes responsible for skin disorders in the winter-born children of higher latitude subgroup were different from those in the at-birth samples. The childhood genes were *MIR-1914*, *MIR-647 and MAP3K8* (common wart) and *TNF* (psoriasis) (Additional file 8: Table S8) [66–68].

A DMR on chromosome 10, identified in the summer-born children of the higher latitude subgroup childhood analysis, was mapped to *FGFR2* gene. Interestingly, alterations in the *FGFR2* gene seen in several cancer types made it a target for the development of person-specific treatments [69–72]. DNAm status in the proximity of the FGFR2 gene, such as the one observed in this study, may further influence the *FGFR2* alterations in the cancer patients. Therefore, knowledge of methylation status around *FGFR2* of the cancer patients will help fine tune the design of the person-specific therapies further.

Several genes mapped to DMRs of at-birth blood samples of the winter-born of the higher latitude, not found in the lower latitude, had links to immunity and inflammation (*LAX1*: T-/B-/NK-cell activation; *TLR5*: pathogen recognition; *LTB4R*: pathogenesis of inflammatory diseases; *PSORS1C3:* psoriasis; *TCL1A*: modulation of immune responses; *ADAM5*: autoimmune diseases) [54, 62–64, 73–76]. *LTB4R*, leukotriene B4 receptor, appears to be the most epigenetically divergent gene in the peripheral blood of humans. *LTB4R* has been linked to a variety of inflammatory diseases such as asthma [77], allergic airway inflammation [78], inflammatory arthritis [79], atherosclerosis [80], inflammatory bowel disease [81] and psoriasis [82]. This is significant as the pregnant mothers of the winter-born babies would have been exposed to various seasonal factors that are responsible for allergy/asthma and other causal exposures of inflammation during the first and the second trimesters of their pregnancy. Lockett et al. hypothesised that the season-associated DNAm of allergic diseases such as eczema most probably arose postnatally since no such association was observed at birth (in cord blood) in their study [26]. However, the lack of evidence for a prenatal association at an epigenome-wide level could also have been due to the modest sample size of their study (*N* = 175).

A CpG, cg003488551, mapped to *C7orf50* and DMR containing this CpG were reported to be associated with prenatal exposure to particulate air pollution in a meta-analysis by Gruzieva et al. [83]. Major air pollutants show seasonal patterns with highest concentrations in the indoor heating seasons of November to February (winter months) in the northern hemisphere [84]. It is possible the summer-born babies in the current study were exposed to indoor pollutants during most part of their in utero life. However, our study did not identify the same methylation signals in babies born in the higher latitude subgroup with extended and extreme winter conditions. This study also did not identify any known vitamin D-associated CpGs or genes mapped to DMRs in cohorts from the higher latitude (≥ 50°N).

### Imprinted genes and metastable epialleles

Our study also identified DMRs associated with three imprinted genes in the at-birth analyses of the summer-born infants of the higher latitude, *GNAS*/*GNAS-AS1*, *MEG3* and *MEST* (*PEG1*). Almost all the CpGs found in the DMRs of these imprinted genes were hyper-methylated. In addition to being season of birth and latitude specific (only in the summer-born babies of the higher latitude), they were not found in the older children from the higher latitude subgroup. However, a DMR associated with the *MEST* gene (mesoderm-specific transcript) was present both at birth (summer-born) and in early childhood (spring-born) from the higher latitude subgroup. Many imprinted genes function as regulators of embryonic or neonatal growth and may therefore influence a spectrum of heritable outcomes later in life. The majority of imprinted genes are expressed in the brain, and methylation of these genes in their imprinting control regions (ICRs) has been implicated in neuropsychiatric disorders (reviewed in [85]). For example, GNAS is a complex imprinted locus with five gene products and multiple DMRs in four of these genes. These DMRs are shown to be associated with a genetic disorder known as pseudohypoparathyroidism type-Ib (PHP-Ib) (reviewed in [86]). Furthermore, a UK Biobank GWAS study of 113,000 individuals with insomnia identified GNAS as a potential gene candidate in females [87]. A previous study reported that the retention of a sex-specific association between a hyper-methylated DMR associated with *MEST* and weight status from birth to early childhood [88].

Metastable epialleles are variably methylated loci with cross-tissue methylation signatures indicative of establishment in the early embryo [44]. They, therefore, provide a useful tool for examining the timing of exposure driven DNAm changes in easily accessible tissues such as blood that may serve as a proxy for patterns of systemic methylation. Silver et al. demonstrated elevated DNAm levels at putative metastable epialleles in rural Gambian children who were conceived during the rainy season compared to those conceived in the dry season [47]. We checked for the enrichment of metastable epialleles in each of the seasons of birth, but none were found, providing no evidence for a season of conception effect at these loci.

### Implications of temporal and latitude-dependent associations between season of birth and differential DNA methylation

In the at-birth meta-analyses, there were only a few robust but weak genome-wide associations between CpG methylation and season of birth. DMR analysis is believed to be statistically more powerful than the analysis of individual probes as it combines methylation signals from nearby CpGs to give more reliable signals for associations between DNAm and exposures. It is possible that the seasonal variations in exposures influence DNAm via groups of CpG sites over an extended region as in the DMRs where contiguous differential methylation may be maintained. Our findings on the birth season-dependent variations of regional epigenetic signals concur with the findings of a study by Dopico et al. which demonstrated the existence of seasonal variations in gene expression profiles over a year in ethnically and geographically diverse populations [21]. They attributed these seasonal variations in gene expression profiles to the seasonal changes in the cellular composition of blood. However, unlike in the study by Dopico et al., our models for association between birth season and DNAm were adjusted for cellular heterogeneity and therefore, the findings are unlikely to be the result of seasonality of blood composition. Epigenetic marks including DNAm signals, whether acting at the individual CpG site or DMR level, are dynamic and may not originate stochastically. Oh & Petronis and Oh et al. proposed that DNAm variability occurring in a periodic fashion over twelve months (circannual oscillations) could contribute to variations in the severity of seasonal diseases [89, 90]. We conclude that our findings on seasonal variations in DNAm signals including DMRs are in line with the circannual oscillations proposed by Oh & Petronis and are likely to reflect the influence of seasonal exposures on later life health events.

### Strengths and Limitations

Our study investigated associations between season of birth and DNAm on a large scale with 9358 at-birth and 3610 childhood samples. Analyses with such a large sample size, especially for the at-birth samples, make it possible to interpret results with more confidence. The cohort-specific EWAS analyses were robust and adjusted for several pre-specified potential confounders, including cell type proportions. The cohorts originated from geographically diverse locations in terms of latitude in the northern hemisphere: 32.7–71.2°N and 36.7–58.7°N for the at-birth and childhood samples. This enabled us to carry out latitude stratifications to investigate season of birth associations of DNAm. Of the 27 cohorts analysed in this study, more than 95% of the participants were of European ancestry. It remains to be seen whether our results are generalizable to other populations.

This study has limitations. This study was designed only to examine the associations between DNAm and season of birth as an exposure and not the factors for which season of birth is a proxy (see above). Heterogeneity of the age range in the childhood population in this study and its smaller sample size (childhood: *N* = 3569 vs. at-birth: *N* = 9358) made it harder to interpret with certainty the absence of at-birth DNAm signals in childhood data. Another limitation of our study is that the at-birth and childhood data were not from the same children for most of the cohorts (21/27 studies). A longitudinal study which follows up the same children at different latitudes in sufficient numbers is necessary to detect the persistence of at-birth DNAm signals from birth to childhood. The latitude analyses of this study have not been adjusted for longitude. The same latitude can have geographic locations that have very different climates, e.g. New York and Madrid (40°N). Furthermore, the choice of autumn as a reference season may have masked some of the other significant associations between season of birth and DNAm. This study was initiated as a follow-on from an earlier study on seasonality effects on asthma and allergy outcomes which showed the strongest effects on allergy phenotypes with autumn as the reference season [26]. However, we expect to observe the circannual oscillations in DNAm even if a season other than autumn was used as the reference season. The cohorts which contributed the EWAS summary results pre-processed and analysed their data using their preferred pipelines and this may have influenced our results. However, Joubert et al. found that their results were robust to different normalization methods used across studies and cell type adjustment [24]. Furthermore, Lussier et al. demonstrated that while different pipelines give different EWAS associations at a set significance threshold their magnitude and directions were consistent [91].

## Conclusions

In this large epigenome-wide meta-analysis study, we provide evidence for an association between season of birth and differential DNAm that is unique for the seasons of the year (temporal effect) and suggestive evidence of a latitude (spatial) effect. Findings in this study add to the understanding of a potential epigenetic role in the seasonality of human disease. Our study suggests the existence of a circannual periodicity in DNAm patterns, much like the seasonal periodicity observed in gene expression profiles.

### Supplementary Information

**Additional file 1. Table S1**: “Summary of the studies in the meta-analysis for the association between Season of Birth and DNA methylation at birth and in children (age: 1 to 11 years)”. Baseline summary of participants from each of the individual cohorts.

**Additional file 2.** “Cohort-specific methods and declarations (cohorts listed in alphabetical order)”. Method description, Funding and Acknowledgements from the participating cohorts.

**Additional file 3.** “PACE analysis plan for Season of Birth and methylation profiles in children (6 June 2018)”. Analysis plan that was circulated amongst the participant cohorts for the Season of Birth study.

**Additional file 4. Table S2**: “Models used in this meta-analysis study”. Inflation (lambda) and bias information of the models. **Table S3**: “Comparison of magnitude and direction of the FDR-significant DNA methylation signals identified in the at-birth and childhood meta-analyses”.

**Additional file 5. Table S4**: “Direction of differential methylation of CpGs in DMRs of the at-birth and childhood analyses (preliminary analysis”).

**Additional file 6. Table S5**: “Genes mapped to DMP/DMR identified in at-birth samples of babies born in the latitude ≥ 50°N and some examples of their associations with biological functions”. Provides examples of known functional associations of genes mapped to significant CpG sites or differentially methylated regions identified in this study.

**Additional file 7. Table S6**: “Season of birth-associated differentially methylated regions (DMRs) at birth in babies born in latitudes < 50°N”. Differentially methylated regions and mapped genes identified in the at-birth samples of babies born in latitudes < 50°N (lower latitude subgroup analysis). **Table S7**: “Season of birth associated with differentially methylated regions (DMRs) in children born in latitudes ≥ 50°N”.

**Additional file 8. Table S8**: “Genes mapped to DMR identified in the childhood samples of children born in the latitude ≥ 50°N and some examples of their associations with biological functions”. Provides examples of known functional associations of genes mapped to significant differentially methylated regions identified in this study.

**Additional file 9.** “Trait Enrichment Analysis (EWAS Atlas)”. Trait names and the odds ratios for their association with CpG sites for all the models meta-analysed in this study.

## Data Availability

Individual cohort-level data can be obtained from the respective cohort (see Additional file 1: Table S1 and Additional file 2 for cohort details).

## Abbreviations

DMR: Differentially methylated region
DNAm: DNA methylation
EWAS: Epigenome-wide association study
FDR: False discovery rate
PACE: Pregnancy And Childhood Epigenetics
SNP: Single nucleotide polymorphism
SES: Socio-economic status
SZ: Schizophrenia
