# Supplementary material for: Analysis of DNA methylation at birth and in childhood reveals changes associated with season of birth and latitude
Source: Clin Epigenetics. 2023 Sep 11;15:148. doi: 10.1186/s13148-023-01542-5 (PMC10496224; doi:10.1186/s13148-023-01542-5)
Supplement: Supplementary file 2 — Additional file 2. “Cohort-specific methods and declarations (cohorts listed in alphabetical order)”. Method description, Funding and Acknowledgements from the participating cohorts. [file 13148_2023_1542_MOESM2_ESM.docx]

**Cohort specific methods and declarations (cohorts listed in alphabetical order)**

Given below are

1. Description of cohorts
2. Definition of covariates
3. Cohort-specific processing methods for DNA methylation data
4. Cohort-specific funding
5. Disclaimer
6. Cohort-specific acknowledgements

Details of the cohort-specific methods for most of the cohorts can also be found in Felix et al. [1]

1. **ALSPAC**

**Description of cohort**

The Avon Longitudinal Study of Parents and Children (ALSPAC; <http://www.bristol.ac.uk/alspac/> ) is a prospective pregnancy cohort study, which enrolled 14 541 pregnant women from Avon, United Kingdom with expected delivery dates between 1 April 1991 and 31 December 1992. Data collected on these women, their partners and their children at regular intervals has been described previously [2,3] and can also be found in the study website which is a fully searchable data dictionary <http://www.bristol.ac.uk/alspac/researchers/our-data/>.

ARIES (Accessible Resources for Integrated Epigenomic Studies, <http://www.ariesepigenomics.org.uk/)> comprises of 1018 mother-offspring pairs selected from the ALSPAC cohort based the availability of their blood samples at multiple time points. DNA methylation data were generated for these individuals also at multiple time points using the Infinium HumanMethylation450 BeadChip array (Illumina Inc., San Diego, United States). This study used child cord blood at birth and whole blood at age 7. Participants with non-white European ancestry were excluded from all analyses. Written consent was collected from the ARIES participants. ALSPAC Ethics and Law Committee and the Local Research Ethics Committees approved the study.

Consent for biological samples has been collected in accordance with the Human Tissue Act (2004). Informed consent for the use of data collected via questionnaires and clinics was obtained from participants following the recommendations of the ALSPAC Ethics and Law Committee at the time.

**Definition of covariates**

Maternal age at delivery was derived from the mother’s date of birth. Maternal SES and education were determined by a questionnaire to the mother, completed during pregnancy, and was collapsed into the following two categories: less than first degree (university) or first degree. Maternal smoking during pregnancy was defined as smoking at least one cigarette per day beyond the first trimester vs no smoking or smoking in the first trimester only and was determined by questionnaire at the time of recruitment. New-born’s sex was obtained from obstetric records so was child’s age. Gestational age was extracted from birth records. Obstetric practice and antenatal care at the time means that for most participants gestational age will have been estimated based on the last menstrual period, supplemented by ultrasound scans and paediatric/obstetric assessment of the new-born at birth. Proportion of six white blood cell types (CD4+ T-lymphocytes, CD8+ T-lymphocytes, NK (natural killer) cells, B-lymphocytes, monocytes and granulocytes) were computed by applying the Houseman method [4] using Bakulski’s [5] and Reinius’ [6] reference panels for cord and child blood samples respectively.

**Generation and pre-processing methods of DNA methylation data**

Generation of DNA methylation data and the methods for data pre-processing in have been described previously [7]. Briefly, blood samples (cord/child) were collected according to standard procedures. DNA samples extracted using standard protocols were subjected to bisulfite-conversion using the Zymo EZ DNA MethylationTM kit (Zymo, Irvine, CA). DNA methylation was then measured using the Infinium HM450 BeadChip assay (Illumina Inc, San Diego, CA) according to standard protocols. Arrays were scanned using an Illumina iScan. An initial review of data quality was assessed using GenomeStudio (version 2011.1). A semi-random approach which ensured that samples from all time points were represented on each array was used to distribute ARIES samples across slides to minimize the possibility of potential confounding by batch. Data were normalised using the meffil R package [8].

**Funding**

The UK Medical Research Council and Wellcome (Grant ref: 217065/Z/19/Z) and the University of Bristol provide core support for ALSPAC. This publication is the work of the authors LK will serve as guarantor for the contents of this paper.

A comprehensive list of grants funding is available on the ALSPAC website (<http://www.bristol.ac.uk/alspac/external/documents/grant-acknowledgements.pdf>). This research was specifically funded by BBI025751/1 and BB/I025263/1, MC_UU_12013/1 & MC_UU_12013/2 & MC_UU_12013/8. EW is funded by the European Union’s Horizon 2020 research and innovation programme (EarlyCause, grant nº 848158) and by CLOSER (grant reference: ES/K000357/1). GCS is financially supported by the Medical Research Council [New Investigator Research Grant, MR/S009310/1] and the European Joint Programming Initiative “A Healthy Diet for a Healthy Life” (JPI HDHL, NutriPROGRAM project, UK MRC MR/S036520/1].

**Disclaimer**

The funders took no role in the design, execution, analysis, or interpretation of the data or in the writing up of the findings.

**Acknowledgements**

We are extremely grateful to all the families who took part in this study, the midwives for their help in recruiting them, and the whole ALSPAC team, which includes interviewers, computer and laboratory technicians, clerical workers, research scientists, volunteers, managers, receptionists, and nurses.

1. **BAMSE**

**Description of cohort**

BAMSE (Children, Allergy, Milieu, Stockholm, Epidemiology in Swedish) is a prospective population-based birth cohort study of children followed throughout childhood and adolescence. Study design, inclusion criteria, enrolment and data collection are described in detail elsewhere [9]. Briefly, 4,089 children born between 1994 and 1996 in four predefined municipalities of Stockholm County were enrolled. At baseline, when the child was approximately 2 months of age, parents completed a questionnaire that assessed residential characteristics, as well as socioeconomic and lifestyle factors. When children were 1, 2, 4, 8, 12 and 16 years, the parents completed questionnaires focusing on children’s symptoms related to wheezing and allergic diseases, as well as various exposures. The survey response rates were 96%, 94%, 91%, 84%, 82% and 78%, respectively [10]. Blood samples were collected from children at ages 4 (n=2,605, 63.7%), 8 (n=2,470, 60.4%) and 16 (2,547, 62.2%) years. Written informed consent was obtained from the parents of all participants. The baseline and follow-up studies were approved by the Regional Ethical Review Board, Karolinska Institutet, Stockholm, Sweden.

**Definition of covariates**

Information on child´s sex, maternal age at delivery, maternal smoking during pregnancy, socioeconomic status for the household (categorized into blue collar worker/white collar worker/other) was assessed by questionnaire at the time of recruitment (at a median age of the children of 2 months). Gestational age was from both the Birth Registry and a questionnaire administered at enrolment based on ultrasound estimations. Doctor´s diagnosis of asthma ever up to 8 years of age was included as selection variable. Maternal smoking was categorised into three groups: never smoked, stopped during early pregnancy, and smoked throughout pregnancy. Proportion of six cord white blood cell types (CD4+ T-lymphocytes, CD8+ T-lymphocytes, NK (natural killer) cells, B-lymphocytes, monocytes and granulocytes) were estimated from the methylation data the *estimateCellCounts* function in the minfi R package based on the algorithm developed by Houseman [4,11] and were included as linear predictors in the regression models. A covariate ‘batch’, based on the bisulfite treatment date at 8 years, was also included in the models to adjust for batch effects.

**Generation and pre-processing methods of DNA methylation data**

This study used data from the 8-year follow-up. DNA samples (500 ng) from peripheral blood collected from 8-year-olds (n=472) were bisulfite converted using the EZ-96 DNA Methylation kit (Zymo Research Corporation, Irvine, USA). Samples were randomized before plating onto the 96-well plates. DNA methylation was measured using the Infinium HumanMethylation450 BeadChip assay (Illumina, San Diego, CA) [1,12]. Quality control of analyzed samples was performed using standardized criteria. Samples with low call rate, low staining efficiency, poor extension efficiency, poor hybridization performance, low stripping efficiency after extension and poor bisulfite conversion were excluded from further analyses. Gender outliers were identified through male-female clusters generated by multidimensional scaling (MDS) plots based on chromosome X data. Samples that did not belong to the distinct clusters were removed. In addition, samples below the 10.5 cutoff in a median intensity plot, generated by the minfi R package for methylated and unmethylated intensities, were also excluded (n=8). Probes were excluded (46799 probes) if i) with a single nucleotide polymorphism in the single base extension site with a frequency of >5% [13], ii) with non-optimal binding (non-mapping or mapping multiple times to either the normal or the bisulphite-converted genome) or iii) from chr X or chr Y. A total of 438713 were included in the analysis. Signal correction and normalization were performed using “DASEN” of the wateRmelon package [14].

**Funding**

BAMSE was supported by The Swedish Research Council, The Swedish Heart-Lung Foundation, MeDALL (Mechanisms of the Development of ALLergy) a collaborative project conducted within the European Union (grant agreement No. 261357), Centre for Allergy Research, Stockholm County Council (ALF), Swedish foundation for strategic research (SSF) (RBc08-0027), the Strategic Research Programme (SFO) in Epidemiology at Karolinska Institutet and the Swedish Research Council Formas. EM is supported by grants from the Swedish Research Council, the Strategic Research Area Epidemiology at Karolinska Institutet. OG is supported by grants from the Swedish Research Council and the Swedish Research Council for Health, Working Life and Welfare (FORTE).

**Acknowledgements**

We would like to thank all the families for their participation in the BAMSE study. In addition,

we would like to thank Alexandra Lövquist, Sandra Ekström, Niklas Andersson and Andre Lauber at the BAMSE secretary for invaluable administrative support, as well as Mutation Analysis Facility (MAF) at Karolinska Institutet for genome-wide methylation analysis, and Ingrid Delin for excellent technical assistance. The computations were performed on resources provided by SNIC through Uppsala Multidisciplinary Center for Advanced Computational Science (UPPMAX) under Project sens2017589.

1. **CBC**

**Description of cohort**

The California Health Cohort (CBC) utilises a repository of neonatal birth bloods as blood dried on a filter paper (Guthrie card, [http://circle.berkeley.edu](http://circle.berkeley.edu/)). CBC study contains two sub-cohorts: a Hispanic sub-cohort and a non-Hispanic one. The repository is maintained by the California Department of Public Health. Data and the blood spot samples are made available to qualified researchers interested in carrying out health-related research as monitored by the local and State level institutional review boards [15].

**Definition of covariates**

Maternal age at delivery was derived from personal interview and confirmed by birth certificates. Maternal socio-economic status (SES) was derived from self-report (personal interview)*.* SES was categorised into 3 groups based on annual household income: US$45,000, 45,000-74999 and >75,000. Maternal smoking during pregnancy was determined through interviews. Smoking was classified into no smoking or any smoking in pregnancy. New-born’s sex was obtained from cancer registry data and confirmed by personal interview of the mother. Gestational age was derived from the birth certificates. Cell type proportions of blood samples from Guthrie cards were estimated from the methylation data by applying the Houseman method using cord blood as reference [4,5].

**Generation of methylation data and pre-processing methods**

Guthrie card DNA was extracted using Qiagen Investigator Kit (blood card protocol). DNA (500 ng) was bisulfite treated using the EZ DNA Methylation-Direct™ Kit (Zymo). Genome-wide DNA methylation was then measured in the bisulfite converted DNA samples using Illumina Infinium HumanMethylation450 BeadChip arrays (Illumina Inc., San Diego, USA, in the UCSF Genomics Core) following to standard protocols. Probes with detection p-values > 0.01 and those missing in >15% of samples were removed as well as samples with >15% failed CpGs. DNA methylation data were pre-processed using the functional normlisation method of Fortin et al [16]. BMIQ normalization was applied to correct for the different probe types [17]. The DNA methylation values were corrected for batch effecting by including the Beadchip IDs in the analyses. Samples were also removed if i) discordance between reported and predicted gender, ii) gender outliers and iii) outliers identified in principal component analysis.

**Funding**

The primary data generation for the CBC work was supported by research grants from the National Institutes of Health (NIEHS R01ES009137, P42ES004705, P01ES018172, P42ES0470518 and R24ES028524), the Environmental Protection Agency (EPA RD83451101). Analytical work at UCSF was supported by National Cancer Institute Cancer Center Support Grant (5P30CA082103) (RR).

**Acknowledgements**

We thank the California Biobank Program at the California Department of Public Health for the specimens and data used in this study, in accordance with Section 6555(b), 17 CCR. The authors acknowledge Robin Cooley and Steve Graham of the California Department of Public Health for their assistance providing banked specimens and record linkage services for this portion of the study. The collection of cancer incidence data used in this study was supported by the California Department of Public Health pursuant to California Health and Safety Code Section 103885; Centers for Disease Control and Prevention’s National Program of Cancer Registries, under cooperative agreement 5NU58DP003862–04/DP003862; the National Cancer Institute’s Surveillance, Epidemiology, and End Results Program under contract HHSN261201000140C awarded to the Cancer Prevention Institute of California, , and contract HHSN261201000034C awarded to the Public Health Institute.

**Disclaimer**

The California Department of Public Health is not responsible for the analyses, results, interpretations, or conclusions drawn by the authors regarding the birth data or samples used in this publication. The ideas and opinions expressed herein are those of the author(s) and do not reflect the opinions of the State of California, Department of Public Health, the National Cancer Institute, and the Environmental Protection Agency or their contractors and subcontractors.

1. **CHAMACOS**

**Description of cohort**

The Center for the Health Assessment of Mothers and Children of Salinas (CHAMACOS; <https://cerch.berkeley.edu/research-programs/chamacos-study> ) is a longitudinal birth cohort established to investigate the association between health and exposure to pesticides and other environmental chemicals, in Mexican-American children from Salinas Valley, CA (see details in [18,19]. Briefly, 601 pregnant women were enrolled at community clinics in 1999-2000 and data on pre-pregnancy and delivery assessments were collected. In addition, the 527 live-born singletons were followed up at regular intervals throughout childhood. The University of California, Berkeley Committee for Protection of Human Subjects approved the study protocols. Oral assent was obtained from children beginning at age 7. Written informed consent was obtained from all mothers as well as written assent from children at age 12.

**Definition of covariates**

Maternal age at delivery and smoking data were derived from the questionnaires at the time of enrolment (on average 13 weeks of pregnancy) and follow up interviews at the time of postnatal visits. None of the mothers included in the analysis reported smoking during pregnancy. SES was defined by educational attainment (less than 7^th^ grade, 7-12 grade, and high school graduate or higher). Gestational age, new-born’s sex and child’s age were collected from medical records. Cell type proportions of blood samples (six blood cell types) from cord and children were estimated from the methylation data using the reference-based Houseman method [4] with Bakulski’s (cord) [5] and Reinius’ (adult) [6] reference panels respectively.

**Generation and pre-processing methods of DNA methylation data**

DNA extracted from blood samples (cord/child) were subjected to bisulfite-conversion using Zymo Bisulfite Conversion Kits (Zymo Research, Irvine, CA). DNA methylation was measured using Illumina Infinium HumanMethylation450 (450K) BeadChips according to manufacturer’s protocol. 450K BeadChips were handled by robotics and analysed using the Illumina Hi-Scan system. DNA methylation was measured at 485,512 CpG sites.

Probe signal intensities were extracted by Illumina GenomeStudio software (version XXV2011.1, Methylation Module 1.9) methylation module and background subtracted. QA/QC procedure was carried out as described by Yousefi et al which included assessment of assay repeatability batch effects using 38 technical replicates [20]. Minimization of colour channel bias, batch effects and difference in Infinium chemistry was achieved by application of ASMN algorithm6, followed by BMIQ normalization [17]. The final dataset contained information on 435,369 CpGs for 158 cord blood and 108 nine-year old blood samples.

**Funding**

The CHAMACOS study was supported by the NIEHS grants (P01ES009605, R01ES021369, R01ES023067, R24 ES028529) and US EPA (R82670901 and RD 83451301)

**Acknowledgements**

We are extremely grateful to all the families who took part in the CHAMACOS study, the field personnel, research scientists and students as well as community partners

1. **CHOP**

**Description of cohort**

Childhood obesity Project (CHOP) is a European multicenter prospective nutritional intervention study of 1678 healthy term new-borns recruited between October 1, 2002, and July 31, 2004. A description of the study design and the prospective data collection can be found in recent publications [21–24]. The local ethics committees of each study center approved all study procedures: Belgium (Comitè d’Ethique de L’Hopital Universitaire des Enfants Reine Fabiola; no. CEH 14/02), Germany (Bayerische Landesärztekammer Ethik-Kommission; no. 02070), Italy (Azienda Ospedaliera San Paolo Comitato Etico; no. 14/2002), Poland (Instytut Pomnik–Centrum Zdrowia Dziecka Komitet Etyczny; no 243/KE/2001), and Spain (Comité ético de investigación clinica del Hospital Universitario de Tarragona Joan XXIII). Written informed consent was obtained and from the children themselves if age 8 or more and from the parents if an infant.

**Definition of covariates**

Maternal age at delivery was derived from medical records and questionnaires during and shortly after delivery. Maternal Socio-economic status (SES) was determined from questionnaire reported highest achieved education. Data on SES was based on maternal education and were grouped into three categories: Low (basic schooling), Medium (a maximum of 10 years of schooling) or High (A levels or University). Maternal smoking during pregnancy was defined as smoking beyond the 12^th^ week of gestation vs no smoking or smoking up to the 12^th^ week only and was derived from questionnaires completed by mothers during the first 8 weeks after delivery. New-born’s sex was obtained from medical records and child’s age from medical records and questionnaires. Gestational age was extracted from medical records during pregnancy. Proportions of six white blood cell types (CD4+ T-lymphocytes, CD8+ T-lymphocytes, NK (natural killer) cells, B-lymphocytes, monocytes and granulocytes) were estimated by applying the Houseman method using Reinius’ reference panels [4,6].

**Generation and pre-processing methods of DNA methylation data**

Generation of DNA methylation data and the methods for data pre-processing in have been described previously [23]. Briefly, blood samples from children aged 5.5 years were collected according to standard procedures. DNA samples, extracted using standard precipitation protocols, were subjected to bisulfite-conversion using the Zymo EZ-96 DNA MethylationTM kit (Zymo, Irvine, CA). DNA methylation was then measured using the Infinium HM450 BeadChip assay (Illumina Inc, San Diego, CA) according to the manufacture’s protocols. Data pre-processing and normalisation were performed according to the method developed by Touleimat and Tost [25] with an adaptation which incorporated the BMIQ method for quantile normalization. In quality control steps only probes with signals from ≥3 beads, detection P-values≤0.01 and samples with ≥80% significant probe methylation signals per sample were retained. In addition, colour bias correction and background adjustment were conducted with R-package lumi. No probe filtering according to proximity of CpG site with SNPs of minor allele frequency ≥5% within 50bp or probes on the X and Y chromosomes were conducted. However, cross-reactive probes were excluded for this analysis [13]. In total, 431313 CpG methylation values for 376 children were available for this EWAS analysis.

**Funding**

The research of the CHOP study reported herein was partially supported by the Commission of the European Community, specific RTD Programme “Quality of Life and Management of Living Resources,” within the 5th Framework Programme (research grant nos. QLRT-2001-00389 and QLK1-CT-2002-30582); the 6th Framework Programme contract no. 007036 (FP6-007036); the European Union’s Seventh Framework Programme Project Early Nutrition under grant agreement no. 289346 (FP7-289346), the Horizon 2020 research and innovation programme DYNAHEALTH (no. 633595) and the European Research Council Advanced Grant META-GROWTH (ERC-2012-AdG – no. 322605). Additional support from the German Ministry of Education and Research, Berlin (Grant Nr. 01 GI 0825) and the University of Munich Innovative Research Priority Project MC-Health is gratefully acknowledged.

**Disclaimer**

This manuscript does not necessarily reflect the views of the Commission and in no way anticipates the future policy in this area. The funders of this study had no role in study design, data collection, data analysis, data interpretation, decision to publish, or preparation of the manuscript.

**Acknowledgements**

We like to thank the participating families and all project partners for their enthusiastic support of the project work. We also like to thank Dr Eva Reischl and team at the Genome Analysis Center of Helmholtz Zentrum Muenchen, Germany for DNA extraction, bisulfite conversion and methylation analysis. We also like to acknowledge The European Childhood Obesity Trial Study Group for their continuous and salient support of the CHOP project: Philippe Goyens, Clotilde Carlier, Joana Hoyos, Pascale Poncelet, and Elena Dain (Universite Libre de Bruxelles – (ULB) –Brussels , Belgium); Jean-Noel Van Hees (CHC St Vincent– Françoise Martin, Annick Xhonneux, Jean-Paul Langhendries, and Jean-Noel Van Hees - Liège-Rocourt, Belgium); Ricardo Closa-Monasterolo, Joaquin Escribano, Veronica Luque, Georgina Mendez, Natalia Ferre, and Marta Zaragoza-Jordana (Universitat Rovira i Virgili, Institut d’Investigacio´ Sanitaria Pere Virgili, Taragona, Spain); Marcello Giovannini, Enrica Riva, Carlo Agostoni, Silvia Scaglioni, Elvira Verduci, Fiammetta Vecchi, and Alice Re Dionigi (University of Milano, Milano, Italy); Jerzy Socha, Piotr Socha and Anna Stolarczyk (Children’s Memorial Health Institute, Department of Gastroenterology, Hepatology and Immunology, Warsaw, Poland); Anna Dobrzanska and Dariusz Gruszfeld (Children’s Memorial Health Institute, Neonatal Intensive Care Unit, Warsaw, Poland); Roman Janas (Children’s Memorial Health Institute, Diagnostic Laboratory, Warsaw, Poland); Emmanuel Perrin (Danone Research Centre for Specialized Nutrition, Schiphol, the Netherlands); Rudiger von Kries (Division of Pediatric Epidemiology, Institute of Social Pediatrics and Adolescent Medicine, Ludwig Maximilians University of Munich, Munich, Germany); Helfried Groebe, Anna Reith, and Renate Hofmann (Klinikum Nurnberg Sued, Nurnberg, Germany); and Berthold Koletzko, Veit Grote, Martina Weber, Peter Rzehak, Sonia Schiess, Jeannette Beyer, Michaela Fritsch, Uschi Handel, Ingrid Pawellek, Sabine Verwied-Jorky, Iris Hannibal, Hans Demmelmair, Gudrun Haile, and Melissa Theurich (Division of Nutritional Medicine and Metabolism, Dr von Hauner Childrens Hospital, Ludwig-Maximilians Universität München (LMU), Munich, Germany).

1. **CHS**

**Description of cohort**

The Children’s Health Study (CHS) is a population-based prospective cohort study from age 5 onwards which recruited 5341 of children born between 1995 and 1997 and aged 5 or older in Southern California [26]. CHS is comprised primarily of Hispanic and non-Hispanic white children who were followed through age 18. Informed written consent and assent were provided by the parents and children respectively. University of Southern California Institutional Review Board approved the study protocol.

**Definition of covariates**

Gestational age, maternal age at delivery, maternal socio-economic status (SES) and child’s sex were obtained from California birth certificates. Data on maternal smoking status during pregnancy and the mother’s social status were collected through questionnaires completed by the parents when the children were approximately 6 years old. Maternal smoking during pregnancy was defined as smoking throughout pregnancy vs no smoking. Maternal SES was defined as less than grade 12/completed grade 12, some college and Completed college/some graduate. Proportions of seven cord blood cell sub-populations (CD8+ T-lymphocytes, CD4+ T-lymphocytes, natural killer cells, B-lymphocytes, monocytes, granulocytes and nucleated red blood cells) were computed by applying the Houseman method [4] using Bakulski’s reference panels [5] for cord blood samples and were included as linear predictors in regression models.

**Generation and pre-processing methods of DNA methylation data**

DNA samples from newborn bloodspots collected at delivery and archived by the state of California, were extracted using the QiaAmp DNA blood kit (Qiagen Inc, Valencia, CA). DNA samples from whole blood cells were treated with bisulfite using the Zymo EZ DNA MethylationTM kit (Zymo, Irvine, CA) according to manufacturer’s protocol. DNA methylation was then measured using the Infinium HM450 BeadChip assay (Illumina Inc, San Diego, CA) according to standard protocols. The results of the Infinium HumanMethylation450 BeadChip (HM450) were compiled for each locus as previously described and were reported as beta (β) values [27]. Quality control of analyzed samples was performed using standardized criteria. Illumina Infinium 450K data were processed using the minfi package (version 1.16.0) in R [11]. Raw intensities were subjected to normal-exponential background correction with dye bias correction to reduce background noise [28]. Beta values were normalized to have the same quantiles to address sample to sample variability [25]. Probes were removed from analyses if they were on the X and Y chromosomes, or if they contained SNPs, deletions, repeats, or if they have more than 10% missing values, leaving a total of 384310 probes in the analysis. No outliers were removed. Seven cord blood cell sub-populations were estimated using regression calibration approach algorithm described by Bakulski *et al.* [5] that was implemented in minfi [29].

**Funding**

NIEHS grants P30ES007048, R01ES022216, K01ES017801

**Acknowledgements**

We would like to express our sincere gratitude to Martin Kharrazi, Steve Graham, and Robin Cooley at the California Biobank Program and Genetic Disease Screening Program within the California Department of Public Health for their assistance and advice regarding new-born bloodspots. The biospecimens and/or data used in this study were obtained from the California Biobank Program, (SIS request number(s) 479)” Section 6555(b), 17 CCR.

**Disclaimer**

The California Department of Public Health is not responsible for the results or conclusions drawn by the authors of this publication.

1. **EAGeR**

**Description of cohort**

The EAGeR Trial is a randomized trial conducted from 2007 to 2011 in the US (ClinicalTrials.gov no. [NCT00467363](https://clinicaltrials.gov/ct2/show/NCT00467363)) [30]. Women were randomized to low dose aspirin or placebo for the outcomes of pregnancy loss and live birth. The trial collected cord blood from deliveries in the Utah site. Previously, the study identified no differences in methylation of cord blood derived DNA by randomization to low dose aspirin [31]. Participants provided written informed consent. Institutional Review Board at each clinical centre and the data coordinating centre approved the study.

**Definition of covariates**

Dates of delivery were collected records from which season of birth was derived. Data on income, education, marital status (for classifying the socio-economic status) and smoking during pregnancy were collected through self-reported questionnaires. Data on maternal age at delivery, gestational age and child’s gender were extracted from hospital records. Maternal smoking during pregnancy was defined as no smoking, smoking but stopped in early pregnancy, smoked throughout pregnancy and started smoking after early pregnancy. Proportions of seven cord white blood cell types (B-cell, CD-4+ T cells, CD-8+ T cells, granulocytes, monocytes, NK-cells, and nucleated RBCs) were estimated using a cord blood specific cell type reference [5].

**Generation and pre-processing methods of DNA methylation data**

Cord blood was collected in a plasma collection tube with ethylenediaminetetraacetic acid (EDTA) [32]. EDTA tubes were processed, and the buffy coat layer stored. DNA from cord blood buffy coat was extracted using GenSolveTM DNA recovery kit. DNA was then subjected to bisulfite conversion using standardized kits (Zymo EZ DNA MethylationTM kit, Zymo, Irvine, CA). This was followed by DNA methylation measurements using the Infinium Methylation EPIC BeadChip microarray according to standard protocols. Methylation data were processed using the minfi package in R including the identification of failed probes and scaling with Illumina control probes to determine methylation values [11].  Quantile normalisation was applied to normalise beta values between two types of probes [25] in order to eliminate potential probe type bias (type I vs II probes).) Samples that are outliers or those with mismatches between predicted sex and sex reported in electronic medical records were identified in principal component analysis (PCA) and were excluded as well as samples and CpG sites with low passing rate (< 97%) based on detection p-value (>0.01) and bead counts (<3). Batch correction was achieved by including plate specifications in the regression models.

**Funding**

The EAGeR trial was supported by the Intramural Research Program of the Eunice Kennedy Shriver National Institute of Child Health and Human Development (National Institutes of Health, Bethesda, MD, USA) under contract numbers HHSN267200603423, HHSN267200603424, HHSN267200603426, and HHSN275201300023I-HHSN2750008.

**Acknowledgements**

The analyses from the EAGeR trial utilized the computational resources of the NIH HPC Biowulf cluster (http://hpc.nih.gov).

1. **EARLI**

Early Autism Risk Longitudinal Investigation cohort (EARLI, <http://www.earlistudy.org/>) is a multicentre prospective study which recruited pregnant mothers and their children amongst whom there is at least one child with a confirmed ASD diagnosis (autistic disorder, Asperger syndrome or pervasive developmental disorder not otherwise specified (PDD NOS) [33]. EARLI study was separated into families of European and non-European ancestry. Non-European ancestry groups were combined because of their small sample sizes. All children were born between November 2009 and March 2012. The mother and children were followed up until the new-borns were three years old. Written informed consent was obtained from all participants. The Human Subjects Institutional Review Boards (IRBs) from each of the four study sites (Johns Hopkins University, Drexel University, University of California Davis, and Kaiser Permanente Northern California) reviewed and approved the study.

**Definition of covariates**

Data on maternal age at delivery, educational level (SES) and smoking during pregnancy were self-reported. No one reported smoking during pregnancy. Maternal SES was inferred based on education and was categorised into four groups – high school or less, some college but no degree, college with a degree and postgraduate degree. Gestational age and new-born’s sex were obtained from medical records. Proportions of seven cord white blood cell types (CD4+ T-lymphocytes, CD8+ T-lymphocytes, NK (natural killer) cells, B-lymphocytes, monocytes, granulocytes, and nucleated red blood cells) were computed by applying the Houseman method [4] using Bakulski’s reference panels [5] for cord blood samples. These were included as covariates in the EWAS models as well as a variable ‘Round’, to adjust for the effect of the two rounds of data collection. In addition, two genomic principal components were also included as covariates in the analysis of non-European ancestry.

**Generation and pre-processing methods of DNA methylation data**

Cord blood DNA was extracted using the DNA Midi kit (Qiagen, Valencia, CA) and samples were subjected to bisulfite conversion and cleaned using the EZ DNA methylation gold kit (Zymo Research, Irvine, CA). DNA was plated randomly and assayed on the Illumina Infinium HumanMethylation450 BeadChip (Illumina Inc., San Diego, USA) at the Johns Hopkins Center for Inherited Disease Research. Pre-processing of raw Illumina image files into noob-background corrected methylation values were carried out using minfi (version 1.18.2) in R [11,34]. Probes with failed detection p-value (>0.01) in >10% of samples and those annotated as cross reactive were removed. Samples with mis-matched reported and predicted (as predicted by sex-chromosomal methylation) gender were removed along with samples that appeared as outliers on the first principal component of methylation data across the genome prior to normalization. Normalized data were corrected for batch effects using ComBat of the sva package (version 3.9.1) [35].

**Funding**

Funding for the EARLI study was provided by the National Institutes of Health (R01 ES016443, PI: Newschaffer; R24ES030893, PI: Fallin; R01ES025531, PI: Fallin) and Autism Speaks (003953 PI: Newschaffer). Mr. Dou and Dr. Bakulski were supported by grants from the National Institutes of Health (R01 ES025531, PI: Fallin; R01 MD013299).

**Disclaimer**

The content is solely the responsibility of the authors and does not necessarily represent the official views of the National Institutes of Health.

**Acknowledgements**

We thank the participants of the EARLI study.

1. **EDEN**

Etudes des Déterminants pré et postnatals précoces du développement et de la santé de l’Enfant (EDEN, https://eden.vjf.inserm.fr/) is a a prospective mother-child cohort which started recruiting pregnant women before their twenty-fourth week of amenorrhea in February 2003 in Poitiers and September 2003 in Nancy. Recruitment lasted 27 months in each centre [36]. The study was reviewed and approved by the ethical committees Comité Consultatif pour la Protection des Personnes dans la Recherche Biomédicale, Le Kremlin-Bicêtre University hospital, and Commission Nationale de l’Informatique et des Libertés.

***Exclusion criteria***

In EDEN cohort infants born more than 42 weeks (more than 294 days) or multiple birth cases were excluded from the study. In the no complication models whose mothers experienced pre-eclampsia hypertension or diabetes (n=8) or delivery start with induction (n=16) or caesarean section (n=18) were excluded. This information was obtained from questionnaires completed by the mothers shortly after delivery and combined with medical birth records.

**Definition of covariates**

Data on maternal age was obtained from both the medical birth registry and a questionnaire administered at the time of enrolment. Data on maternal socio-economic status (SES) and smoking during pregnancy were collected from self-administered questionnaires. Maternal SES was categorised into 3 groups based on the participants’ monthly income – low (<1500 euros), medium (1500-3000 euros) and high (>3000 euros). Maternal smoking was categorised as never smoked, stopped smoking during pregnancy and smoked throughout pregnancy. Gestational age and new-born’s age and sex were obtained from medical records. Six different cell type proportions of cord and child blood samples (CD8+ T, CD4+ T lymphocytes, natural killer cells, B cells, monocytes, and granulocytes) were estimated using the ’estimateCellCounts’ function of Minfi package [29] in R [37] by applying Houseman method [4] and Bakulski’s [5] and Reinius’ [6] reference panels respectively. The estimated cell types of proportions were included as covariates in the EWAs analyses. In addition, five principal components, derived from factor analyses restricted to negative control genes, and a covariate, ‘batch’, based on bisufite treatment were also included as predictors in the regression models.

**Generation and pre-processing methods of DNA methylation data**

[Cord blood](https://www.sciencedirect.com/topics/medicine-and-dentistry/cord-blood) serum samples were collected by research midwives immediately after delivery. Whole blood samples were collected from children aged 5 years. Genomic DNA from cord and children of child blood samples were extracted using the QIAamp blood kit (Qiagen or equivalent protocols), followed by precipitation-based concentration using GlycoBlue (Ambion). The DNA samples were subjected to bisulfite conversion using the EZ-96 DNA methylation kit (Zymo Research, Irvine, CA). Samples were randomised before plating on to 96-well plates to avoid batch effects and assayed on the Illumina Infinium HumanMethylation450 BeadChip (Illumina Inc., San Diego, USA) [38].

***Variables for batch correction***

Batch correction was attained including the significant (permutation p-value< 10^-4^) principal components (PCs) derived from the 613 negative control probes [39] presented in 450K arrays because these negative control probes did not relate to biological variation. After 10000 permutations 5 PCs were retained. The beta-values were batch corrected incorporating these 5 PCs and calculating the residuals of the linear model at 5 years [40]. The covariate batch was also accounted for in the models, based on the bisulfite treatment.

**Funding**

We acknowledge all funding sources for the EDEN study: Foundation for Medical Research (FRM), National Agency for Research (ANR), National Institute for Research in Public Health (IRESP: TGIR cohorte santé 2008 program), French Ministry of Health (DGS), French Ministry of Research, Inserm Bone and Joint Diseases National Research (PRO-A) and Human Nutrition National Research Programs, Paris–Sud University, Nestlé, French National Institute for Population Health Surveillance (InVS), French National Institute for Health Education (INPES), the European Union FP7 programmes (FP7/2007-2013, HELIX, ESCAPE, ENRIECO, MEDall projects), Diabetes National Research Program (through a collaboration with the French Association of Diabetic Patients (AFD)), French Agency for Environmental Health Safety (now ANSES), Mutuelle Générale de l’Education Nationale (MGEN), French National Agency for Food Security, the EU FP7-ENV funded Health and Environment-wide Associations based on Large population Surveys (HEALS) project (N: 603946) and the French-speaking association for the study of diabetes and metabolism (ALFEDIAM).

**Disclaimer**

The funders had no role in study design, data collection and analysis, decision to publish, or preparation of the manuscript.

**Acknowledgements**

We thank the midwife research assistants (L. Douhaud. S. Bedel. B. Lortholary. S. Gabriel. M. Rogeon and M. Malinbaum) for data collection and P. Lavoine for checking, coding and entering data.

1. **EXPOsOMICS**

EXPOsOMICS is a population based European collaborative project which combined data from three birth cohorts: ENVIRONAGE (Belgium), Rhea (Greece) and Piccolipiu (Italy). Heterogeneity between the three cohorts was minimised by harmonising the phenotypic variables across the three cohorts and randomising the DNA samples on the DNA methylation arrays. Ethical approval for the Exposomics studies was obtained from IARC Ethics Committee (IEC 13-16) as well as by the local ethics committees of each cohort as described below. We thank Dr. Paolo Vineis for coordinating the EXPOsOMICS project and Mr. Cyrille Cuenin and Mr. Vincent Cahais for their help in this project. The DNA methylation assays were funded by the European Community's Seventh Framework Programme FP7/2007–2013 project EXPOsOMICS (grant no. 308610).

***Piccolipiu***

**Description of cohort**

Piccolipiu (([www.piccolipiu.it](http://www.piccolipiu.it)) is a multicenter Italian birth cohort that recruited 3338 new-borns and their mothers in 5 centres: Turin, Trieste, Florence, Viareggio and Rome between 2011 and 2015 [41]. Pregnant women with singleton pregnancy were eligible for inclusion if they were at least 18 years old, were scheduled to give birth in one of the selected hospitals participating in the study, had residence in the catchment area of the maternity centre, ability to fill out the informed consent and the questionnaire in Italian and had a telephone number to be reached at. Women were recruited at the end of pregnancy/at delivery. For each new-born, whole blood was withdrawn from cord vessels and collected in a tube with EDTA (Ethylene Diamine Tetra Acetic acid), fractionated in buffy coat, plasma and erythrocytes and stored in a biobank at -80◦C. Written informed consent was collected from parents of the study participants. The Ethics committees of the Local Health Unit Roma E (management centre), of the Instituto Superiore di Sanità (National Institute of Public Health) and of each local centre gave ethical approval to the study.

**Generation and pre-processing methods of DNA methylation data**

Cord blood DNA was extracted (QIAamp 96 DNA Blood Kit, Qiagen 51161), quantified (Quant-iT PicoGreen dsDNA Assay Kit, Molecular Probes P7589) and bisulfite converted (600 ng of DNA using EZ-96 DNA Methylation kit, Zymo Research D5004). DNA methylation was measured using Illumina Infinium HumanMethylation450 BeadChip (Illumina Inc., San Diego, USA). The arrays were designed such that batch effects (e. g. sample position and intra- and inter-variability in arrays and chips) do not completely confound the biological covariates of interest. This design allows the retention of biological variation even after correction of technical variation. Specifically, each chip incorporated proportional amounts of samples representing the three different cohorts (RHEA, Environage and Piccolipiu).

Raw intensity (.idat) files were handled in R using the minfi-package to calculate the methylation levels at each CpG as beta-values

Intensity of the methylated allele (M)

beta= ------------------------------------------------------------------------------------------------------------

(Intensity of the unmethylated allele (U) + intensity of the methylated allele (M) + 100).

Methylation features were filtered from cross-reactive probes and low-quality probes (probes having bead counts<3 in at least 5% of samples). Data quality was further assessed using box plots for the distribution of methylated and unmethylated signals. Sample outliers and potential gender mismatches, identified by multidimensional scaling plots and unsupervised clustering, were removed from the analysis. Also, samples having >1% of CpG sites with a detection P-value >0.05 were removed. The remaining dataset was normalized using the funnorm normalization of the minfi package. Batch effects were then corrected with surrogate variable analysis (SVA). QQ-plots of p- values were used to rule out potential data inflation after statistical analyses.

**Funding**

Piccolipiù cohort was approved and initially funded by the Italian National Centre for Disease Prevention and Control (CCM grant 2010) and by the Italian Ministry of Health (art 12 and 12bis Dl.gs.vo 502/92).

**Acknowledgements**

We are extremely grateful to all the families who took part in this study, to the midwives for their help in recruiting them, and to the whole 'PICCOLIPIU’ team, which includes doctors, nurses, researchers and computer/laboratory technicians.

***RHEA***

**Description of cohort**

Rhea ([www.rhea.gr](http://www.rhea.gr)), established in 2007 in Crete, is a mother-child pair cohort recruited at the early stages of pregnancy and followed up to young adulthood [42]. The study aims are to evaluate (a) nutritional, environmental, biological and psychosocial exposures in the prenatal period and in early childhood, (b) the association of these exposures with the development of the foetus and the child, (c) mother’s health during and after pregnancy, and (d) genetic susceptibility and the interactions between genetic and environmental factors affecting child health. At each visit written informed consent was obtained from the mothers, and the Ethics Committee of the University Hospital at Heraklion approved the study protocols.

**Definition of covariates**

We collected information about maternal and child covariates via personal interviews, together with self-administered questionnaires and a review of medical records: maternal age at delivery; maternal education [low level: ≤9 years of mandatory schooling, medium level: >9 years of schooling up to attending post‐secondary school education (but not attending university or having a technical college degree) and high level: attending university or having a university/technical college degree]; smoking during pregnancy (current smoker/ex‐smoker/never smoker) and child sex. Gestational age (length of gestation) was based on the interval between the last menstrual period and the date of delivery of the baby for most of the subjects. When the menstrual estimate of gestational age was inconsistent by ≥7 days with the ultrasound measurement taken in the first trimester of pregnancy, a quadratic regression formula describing the relationship between crown–rump length and gestational age was used instead.

**Generation and pre-processing methods of DNA methylation data**

Cord blood DNA was extracted (QIAamp 96 DNA Blood Kit, Qiagen 51161), quantified (Quant-iT PicoGreen dsDNA Assay Kit, Molecular Probes P7589) and bisulfite converted (600 ng of DNA using EZ-96 DNA Methylation kit, Zymo Research D5004). DNA methylation was measured using Illumina Infinium HumanMethylation450 BeadChip (Illumina Inc., San Diego, USA). Each chip incorporated proportional amounts of samples representing the three different cohorts (RHEA, Environage and Piccolipiu). Raw intensity (.idat) files were handled in R using the minfi-package to calculate the methylation levels at each CpG as beta-values. Methylation features were filtered from cross-reactive probes and low-quality probes (probes having bead counts <3 in at least 5% of samples). Data quality was further assessed using box plots for the distribution of methylated and unmethylated signals. Sample outliers and potential gender mismatches, identified by multidimensional scaling plots and unsupervised clustering, were removed from the analysis. Also, samples having >1% of CpG sites with a detection p-value >0.05 were removed. The remaining dataset was normalized using the funnorm normalization of the minfi package.

**Funding**

The Rhea project was financially supported by European projects (EU FP6-003-Food-3-NewGeneris – Contract no. 16320, EU FP6 STREP Hiwate – Contract no. 36224, EU FP7 ENV.2007.1.2.2.2. – Project no. 211250 Escape, EU FP7-2008-ENV-1.2.1.4 Envirogenomarkers – Contract no. 226756, EU FP7-HEALTH-2009-single stage CHICOS – Contract no. 241604, EU FP7 ENV.2008.1.2.1.6. – Proposal no. 226285 ENRIECO, EU-FP7 – Proposal no. 264357 MeDALL, EU-FP7-HEALTH-2012 – Proposal no. 308333 HELIX) and the Greek Ministry of Health (Program of Prevention of obesity and neurodevelopmental disorders in preschool children, in Heraklion district, Crete, Greece: 2011–2014; ‘Rhea Plus’: Prevention Program of Environmental Risk Factors for Reproductive Health, and Child Health: 2012–2015).

**Acknowledgements**

The authors would like to thank all study participants and the doctors, nurses, midwives, and laboratory technicians who assisted with its conduct, for their generous collaboration.

***ENVIRONAGE***

**Description of cohort**

ENVIRONAGE (ENVIRonmental influence ON early AGEing) is an ongoing study that was initiated in 2010 to investigate human aging and its interaction with the environment [43]. ENVIRONAGE recruited mother-new-born pairs at delivery in East-Limburg hospital in Genk (Belgium). The Ethical Committee of Hasselt University and the East-Limburg Hospital approved the study. Inclusion criteria were delivery without planned caesarean section and ability to fill out a Dutch language questionnaire.

**Definition of covariates**

At delivery the following information on parents and children were collected using questionnaires completed by the mothers: maternal age (years); maternal smoking status during pregnancy (any smoking in pregnancy/no smoking in pregnancy) and maternal education (mothers were categorised into three groups based on their educational levels: Low: no education, Middle: high school diploma, and college, High: university degree or higher). Sex of the child and gestational age (based on ultrasound) were derived from obstetric data. Cell type composition was estimated using filtered and combined reference dataset available via Bioconductor as “FlowSorted.CordBloodCombined.450 k” [44].

**Generation and pre-processing methods of DNA methylation data**

Cord blood DNA was extracted (QIAamp 96 DNA Blood Kit, Qiagen 51161), quantified (Quant-iT PicoGreen dsDNA Assay Kit, Molecular Probes P7589) and bisulfite converted (600 ng of DNA using EZ-96 DNA Methylation kit, Zymo Research D5004). DNA methylation was measured using Illumina Infinium HumanMethylation450 BeadChip (Illumina Inc., San Diego, USA). Each chip incorporated proportional amounts of samples representing the three different cohorts (RHEA, Environage and Piccolipiu). Raw intensity (.idat) files were handled in R using the minfi-package to calculate the methylation levels at each CpG as beta-values. Methylation features were filtered from cross-reactive probes and low-quality probes (probes having bead counts <3 in at least 5% of samples). Data quality was further assessed using box plots for the distribution of methylated and unmethylated signals. Sample outliers and potential gender mismatches, identified by multidimensional scaling plots and unsupervised clustering, were removed from the analysis. Also, samples having >1% of CpG sites with a detection p-value >0.05 were removed. The remaining dataset was normalized using the funnorm normalization of the minfi package.

**Funding**

The ENVIRonAGE birth cohort is funded by the European Research Counsil (ERC-2012-StG.310898) and by funds of the Flemisch Scientific Research Council (FWO, N1516112 / G.0.873.11N.10 / G059219). The methylation assays were funded by the European Community's Seventh Framework Programme FP7/2007-2013 project EXPOsOMICS (grant no. 308610). .

**Disclaimer**

Where authors are identified as personnel of the International Agency for Research on Cancer/ World Health Organization, the authors alone are responsible for the views expressed in this article and they do not necessarily represent the decisions, policies, or views of the International Agency for Research on Cancer/ World Health Organization.

**Acknowledgement**

The authors acknowledge the participating mothers and neonates, as well as the staff of the maternity ward, midwives, and the staff of the clinical laboratory of East-Limburg Hospital in Genk. The authors acknowledge Dr Paolo Vineis for his input and the coordination of EXPOsOMICS

1. **FLEHS1**

**Description of cohort**

The Flemish Environment and Health Study 1 (FLEHS1; 2002–2006, <http://www.milieu-en-gezondheid.be/en/home>) was established for human biomonitoring in different geographically representative samples of the Flemish population in Belgium [45]. The study recruited mother-newborn pairs to monitor early-life exposures, adolescents of 14–15 years, and adults (50-65 years) to monitor exposures that are more specific for these periods in life [46]. Data were collected on chemical exposures and their early biological effects, data on lifestyle, health, and environment of the participants. Cord blood samples were collected at delivery and stored in a biobank for future investigations. Blood and saliva samples were collected from a subset of the child participants at age 10 years and questionnaire data at the age of 11 years. Informed consent was provided by all participating mothers and the campaign was approved by the ethical committee of the University of Antwerp. During follow-up of the cohort at 10 years of age (n=595) data on growth, diet, physical activity, medical conditions, and lifestyle were gathered.

**Definition of covariates**

Data on maternal age at delivery, new-born gender and gestational age, maternal education and smoking during pregnancy were collected via self-reported questionnaires or medical records of mothers/children. Maternal smoking during pregnancy was categorised as never smoked or smoked part or throughout pregnancy. Mothers were categorised into two groups based on their educational levels: a maximum of secondary education or higher non-university/university education. Cell type proportions of blood samples from cord and children (CD4+ T-lymphocytes, CD8+ T-lymphocytes, NK (natural killer) cells, B-lymphocytes, monocytes and granulocytes) were estimated by applying the Houseman method [4] using Bakulski’s [5] and Reinius’ [6] reference panels for cord and child blood samples respectively. Batch numbers were included as covariates in the models when necessary to adjust for batch effects.

**Generation and pre-processing methods of DNA methylation data**

Whole genomic DNA was extracted from peripheral blood mononuclear cells (PBMC) isolated from cord blood and blood collected at age 11 years using Lymphoprep™ (Axis-Shield, Oslo, Norway). The DNA samples were subjected to bisulfite conversion using the EZ DNA methylation kit (Zymo Research, Cambridge Bioscience, Cambridge, UK) according to manufacturer’s instructions. Genome-wide DNA methylation profiles were generated with Infinium HumanMethylation450 BeadChip Array (Illumina, San Diego, CA, USA) according to the standard Infinium HD Assay Methylation Protocol Guide (Part #15019519, Illumina). The raw methylation intensities were extracted as methylation β-values (ranging from 0, unmethylated, to 1, fully methylated) using GenomeStudio Methylation Module software without background correction and normalization. Processing of raw data, quality control and normalisation were performed using “minfi” R-package. Briefly, the raw Red/Green channel data from the 450K-llumina methylation array were read by the ‘read.450k.exp’ function, converted to methylation values by ‘preprocessRaw’ and subsequently normalized using ‘preprocessSWAN’, an implementation of the Subset-quantile Within Array Normalization (SWAN) normalization procedure [47]. Sample outliers and gender mismatches identified in Principal Component analysis and unsupervised clustering as well as samples having >75% of CpG sites with detection p-value> 1X10^-5^ were removed. Probes with a detection p-value greater than 0.01 in all samples or on the sex chromosomes were also removed. 470,562 sites were retained from the original 485,512 sites.

**Funding**

We thank FLEHS Supervisory Board for the provision of data. The FLEHS studies were commissioned, financed, and steered by the Flemish Government (Department of Economy, Science and Innovations, Agency for Care and Health and Department of Environment). The methylation work was funded by the CEFIC LRI award 2013 that was given to Dr Sabine Langie. SL is also a recipient of a post-doctoral fellowship [12L5216N; http://www.fwo.be/] provided by The Research Foundation-Flanders (FWO) and the Flemish Institute for Technological Research (VITO).

**Acknowledgements**

The authors thank all the children and their parents for their cooperation, and all the field workers and laboratory personnel involved for their efforts. The authors also thank the groups of Guy Van Camp and Wim Vanden Berghe, University of Antwerp, for their support in the methylation analysis, and the group of Diether Lambrechts at the VIB-KU Leuven Center for Cancer Biology for the bioinformatics analysis.

1. **Gen3G**

**Description of cohort**

Genetics of Glucose Regulation in Gestation and Growth (Gen3G) is a prospective observational cohort study initiated in January 2010 in Canada with aim of investigating genetic determinants of glucose regulation during pregnancy and their impact on fetal development [48]. The study recruited a total of 1024 pregnant women. Written informed consent was obtained from the participants before enrolment in the study in accordance with the Declaration of Helsinki. Centre Hospitalier Universitaire de Sherbrooke (CHUS) ethic committee board approved the study protocol.

**Definition of covariates**

Data on maternal smoking and maternal age at birth were derived from self-reported questionnaires during first trimester of pregnancy. Maternal smoking was categorised as ‘never smoked’, ‘prior smoking, but stopped before 1^st^ trimester’, and ‘smoking’ (as self-reported at 1^st^ trimester visit). Child sex and gestational age at delivery were abstracted from electronic medical records. Maternal social class was not available for Gen3G. The six cell type proportions of cord blood (CD4+ T-lymphocytes, CD8+ T-lymphocytes, NK (natural killer) cells, B-lymphocytes, monocytes and granulocytes) were estimated from the methylation data using the Reinius-based Houseman method [4,6] with the *estimateCellCounts* function in the Minfi package [29] in R [37].

**Generation and pre-processing methods of DNA methylation data**

DNA samples were isolated from cord blood using the Gentra Puregene Blood Kit (Qiagen, Mississauga, ON, Canada). DNA was quantified on a Beckman Coulter DTX 880 spectrophotometer using the Quant-iT™ PicoGreen® dsDNA assay kit (Life Technologies (Invitrogen), Burlington, ON, Canada) following the manufacturer’s protocol. DNA samples were bisulfite-converted, and genome-wide DNA methylation levels assayed using HumanMethylation450 BeadChips (Illumina, Inc., San Diego, CA, USA). Outliers (based on multidimensional scaling plot), sex mismatch and samples with more than 5% of missing values across the epigenome (detection p-value >0.01) were removed. Probes with a detection p-value of <0.01 in ≥80% of the samples were also excluded from subsequent analyses. After quality control, methylation data were available for 176 cord blood samples. Beta values were normalised using DASEN normalization from the wateRmelon R package [14].

**Funding**

This work was supported by a Fonds de recherche du Québec - Santé (FRQS) operating grant (to M-FH, grant #20697); a Canadian Institute of Health Research (CIHR) operating grant (to M-FH grant #MOP 115071 and to LB #PJT-152989); a Diabète Québec grant (to PP).

**Acknowledgements**

We are thankful to all Gen3G mothers and children for their participation, time, and commitment.

1. **The Generation R**

**Description of cohort**

The Generation R Study (Gen R) is a population-based prospective cohort study from the Netherlands and is designed to identify early environmental and genetic causes of growth, development and health during foetal life, childhood and adulthood [49]. The study recruited pregnant women residing in Rotterdam with a delivery date between April 2002 and January 2006 as well as their new-born children. These children are to be followed up until young adulthood. Data on children were collected through home visits, questionnaires and routine visits to the child health centres. Written informed consent was obtained from the parents or legal representatives of the participating children. The Medical Ethical Committee of Erasmus MC, University Medical Centre, Rotterdam approved the study. Gen R enrolled 9778 mothers.

**Definition of covariates**

Data on maternal educational level was obtained from questionnaires in early pregnancy and was classified into two groups – low/middle (low: no or only primary school education; middle: high school or community college) and high (higher education or university level). Maternal smoking during pregnancy was assessed by questionnaires in each trimester of pregnancy and was categorised into three groups – never smoked, stopped during pregnancy, smoked throughout pregnancy. Data on mother’s age was collected through questionnaires. Data on gestational age at delivery and sex of the new-borns were collected through midwife or hospital records. Current age of the children included in this analysis were recorded/measured at the study research centre. The relative proportions of six white blood cell types (CD4+ T-lymphocytes, CD8+ T-lymphocytes, NK (natural killer) cells, B-lymphocytes, monocytes and granulocytes) from cord and 5-year olds were estimated by applying the reference-based Houseman method [4] in the minfi package in R with appropriate reference panels (Bakulski’s [5] and adult Reinius [6] reference panels for cord and 5-year olds’ respectively).

**Generation and pre-processing methods of DNA methylation data**

DNA extracted (using the salting-out method) from blood samples taken at birth (cord blood) or at the 5-year follow up was used in this analysis. 500 ng DNA per sample was subjected to bisulfite conversion using the EZ-96 DNA Methylation kit (Shallow) (Zymo Research Corporation, Irvine, USA). Samples were plated onto 96-well plates in no specific order. Samples were processed with the Illumina Infinium HumanMethylation450 BeadChip (Illumina Inc., San Diego, USA) which analyses methylation at 485,577 CpG sites. Preparation and normalization of the HumanMethylation450 BeadChip array data was performed according to the CPACOR workflow1 using the software package R [50]. In detail, the idat files were read using the minfi package. Probes that had a detection p-value above background (based on sum of methylated and unmethylated intensity values) ≥1E-16 were set to missing per array. Next, the intensity values were stratified by autosomal and non-autosomal probes and quantile normalized for each of the six probe type categories separately: type II red/green, type I methylated red/green and type I unmethylated red/green. Beta values were calculated as proportion of methylated intensity value on the sum of methylated+unmethylated+100 intensities. Arrays with observed technical problems such as failed bisulfite conversion, hybridization, or extension, as well as arrays with a mismatch between sex of the proband and sex determined by the chr X and Y probe intensities were removed from subsequent analyses. Additionally, only arrays with a call rate >95% per sample were processed further. Probes on the X and Y chromosomes were excluded from the dataset. The final dataset contained information on 458,563 CpGs for 1396 samples at birth and 493 samples at age 5. Adjustment for batch effect was done by including plate number as a covariate in the model.

**Funding**

The general design of the Generation R Study is made possible by financial support from the Erasmus MC, Erasmus University Rotterdam, the Netherlands Organization for Health Research and Development and the Ministry of Health, Welfare and Sport. The EWAS data were funded by a grant to prof. dr. V.W.V. Jaddoe from the Netherlands Genomics Initiative (NGI)/Netherlands Organisation for Scientific Research (NWO) Netherlands Consortium for Healthy Aging (NCHA; project nr. 050-060-810), by funds from the Genetic Laboratory of the Department of Internal Medicine, Erasmus MC, and by a grant from the National Institute of Child and Human Development (R01HD068437). This project received funding from the European Union’s Horizon 2020 research and innovation programme (733206, LIFECYCLE; 874739, LongITools; 824989, EUCAN-Connect) and from the European Joint Programming Initiative “A Healthy Diet for a Healthy Life” (JPI HDHL, NutriPROGRAM project, ZonMw the Netherlands no.529051022 and PREcisE project ZonMw the Netherlands no.529051023).

**Acknowledgements**

We gratefully acknowledge the contribution of children and parents, general practitioners, hospitals, midwives, and pharmacies in Rotterdam. We thank Mr. Michael Verbiest, Ms. Mila Jhamai, Ms. Sarah Higgins, Mr. Marijn Verkerk and Dr. Lisette Stolk for their help in creating the EWAS database. We thank Dr. A.Teumer for his work on the quality control and normalization scripts. The Generation R Study is conducted by Erasmus MC, University Medical Center Rotterdam in close collaboration with the School of Law and Faculty of Social Sciences of the Erasmus University Rotterdam, the Municipal Health Service Rotterdam area, Rotterdam, the Rotterdam Homecare Foundation, Rotterdam and the Stichting Trombosedienst & Artsenlaboratorium Rijnmond (STAR-MDC), Rotterdam. The generation and management of the Illumina 450K methylation array data (EWAS data) for the Generation R Study was executed by the Human Genotyping Facility of the Genetic Laboratory of the Department of Internal Medicine, Erasmus MC, the Netherlands.

1. **GOYA**

**Description of cohort**

The Genetics of Overweight Young Adults (GOYA) study has been described previously [51]. Briefly, it includes a case-cohort sampled subset of 91,387 pregnant women recruited into the Danish National Birth Cohort (DNBC; <https://www.dnbc.dk> ) between 1996 and 2002. GOYA cases were selected as the 3.6% of DNBC mothers with the largest residuals from the regression of BMI on age and parity from the total of 67,853 eligible women who had given birth to a live infant, provided a blood sample during pregnancy, and had BMI information available. BMI for these 2,451 women “cases” ranged from 32.6 to 64.4. A similar number of women (n=2,450) were sampled from the remaining cohort as controls. DNA methylation data were generated for the offspring of 1000 case and control mothers in the GOYA study. Current analysis among participants with DNA methylation available was restricted to a randomly selected sub-group with a normal BMI distribution to avoid confounding by substructure. Analysis was further restricted to new-borns with all relevant covariates available.

**Definition of covariates**

Data on maternal covariates were collected via a telephone interview at around 16 weeks of gestation. Maternal smoking during pregnancy was defined as any or no smoking, in pregnancy. Maternal age at delivery was derived from self-reported date of birth. Socioeconomic status was defined using maternal education or occupation as follows: i) manager or medium to long education, ii) work requiring a short training period, or skilled manual labour, iii) unskilled. Data on gestational age at delivery and the new-born’s gender were obtained from birth record register information. Proportion of seven different cord white blood cell types (CD8+ T and CD4+ T lymphocytes, CD56+ natural killer cells, CD19+ B cells, CD14+ monocytes, granulocytes, nucleated red blood cells) were computed by the Houseman method [4] using the cord blood reference dataset [5] and the default implementation of the estimateCellCounts function in the *minfi* package [11]. Ten surrogate variables were generated and were included in models to adjust for technical batch effects.

**Generation and pre-processing methods of DNA methylation data**

Cord blood was collected according to standard procedures, spun and frozen at -80˚C. DNA methylation analysis and data pre-processing for cord blood samples were performed at the University of Bristol. Following extraction, DNA was bisulfite converted using the Zymo EZ DNA MethylationTM kit (Zymo, Irvine, CA). Methylation status was then measured using the Illumina Infinium® HumanMethylation450k BeadChip assay according to standard protocol. The arrays were scanned using an Illumina iScan and initial quality review was assessed using GenomeStudio (version 2011.1).

All DNA methylation results were cleaned and normalized in R (version 3.3.0) using the *meffil* package [8]. Samples were excluded from downstream analysis if >10% of probes had a p-value ≥ 0.01. CpGs were excluded if their probe detection p-values were ≥0.01 across >10% of samples. Sample sex was assessed by comparing HumanMethylation450k genotype probes to previous SNP-chip data and comparing median chromosome Y and chromosome X probe intensities. Samples were excluded if they failed these checks. Data were normalized using the functional normalization approach in the *minfi* R package [11]. Probes with values > 3*interquartile range were also excluded from analyses. Level of methylation measurements were expressed as β-values ranging from 0 (no cytosine methylation) to 1 (complete cytosine methylation).

**Funding**

The Danish National Birth Cohort was established with a significant grant from the Danish National Research Foundation. Additional support was obtained from the Danish Regional Committees, the Pharmacy Foundation, the Egmont Foundation, the March of Dimes Birth Defects Foundation, the Health Foundation, and other minor grants. The DNBC Biobank has been supported by the Novo Nordisk Foundation and the Lundbeck Foundation. Generation of DNA methylation data was funded by the Medical Research Council Integrative Epidemiology Unit at the University of Bristol (MC_UU_00011/5) and the University of Bristol.

**Acknowledgements**

The authors are grateful to all GOYA (Genomics of Obesity in Young Adults) participants and collaborative team members that have contributed to the study.

1. **HAVEN**

**Description of cohort**

The HAVEN study, a Dutch acronym for the study of heart anomalies and the role of genetic and nutritional factors, is a case–control study designed to investigate determinants in the pathogenesis and prevention of CHD and described in detail before [52]. In short, participants were children born in the western part of The Netherlands between January 2002 and June 2007. During a standardized hospital visit around 17 months of age, venous blood samples were drawn from the children and the questionnaires filled out by the mother at home were checked for completeness and consistency. All parents gave written informed consent on behalf of their participating child. The study was approved by the Central Committee for Human Research in The Hague, The Netherlands, and by the Institutional Review Boards (Medical Ethics Committees) of the Erasmus MC University Medical Center in Rotterdam, Leiden University Medical Center in Leiden, VU University Medical Center and Academic Medical Center in Amsterdam, The Netherlands. DNA methylation was measured for children born with a congenital heart defect (N=84) and children born without congenital heart defects (N=196). This study made use of the methylation data from the control children of Dutch descent with complete data for the covariates required for this meta-analysis.

**Definition of covariates**

Season of birth was defined following the analysis plan. Data on mother’s age, maternal smoking during pregnancy and socio-economic status were obtained via questionnaires and coded as instructed. Gestational age at delivery and sex of the new-borns were collected from medical records of delivery and the child’s age was calculated from the date of birth in the medical record. Maternal smoking during pregnancy was defined as no reported smoking, stopped smoking during pregnancy or continued smoking during pregnancy. Proportion of six different blood cell types (CD8+ T and CD4+ T lymphocytes, CD56+ natural killer cells, CD19+ B cells, CD14+ monocytes, granulocytes) were computed by the Houseman method [4] using IDOL. These were included as covariates in the EWAS models. In addition, several technical covariates, namely the unique factorial combinations of bisulfite plate and scan batch, a variable denoting the height of the array on a glass slide (continuous variable from 1-6) and a variable denoting the two different dye lot numbers, were also used to adjust the EWAS models.

**Generation and pre-processing methods of DNA methylation data**

DNA was extracted from whole blood samples according to standard protocols and was subjected to bisulfite conversion using the Zymo EZ DNA MethylationTM kit (Zymo, Irvine, CA). Briefly, samples were randomly distributed per 96-well plate and 450K array. Methylation status was measured using the Illumina Infinium® HumanMethylation450k BeadChip assay according to manufacturer’s instructions at the Erasmus University Medical Center array facility. Quality control and normalisation of beta values were carried out using a publicly available pipeline, DNAmArray (Tobi et al., IJE 2015; https://github.com/molepi/DNAmArray). Sample dependent and sample independent quality metrics were assessed using the R package MethylAid [53] and bisulfite conversion efficiency was assessed using dedicated probes on the array. Sample swaps were excluded by examining the genotypes measured on the 450K array via the R package omicsPrint [54] and gender assessment using X-chromosomal CpG dinucleotides. Individual measurements with a detection P-value >0.01 or zero intensity value in the used colour-channel were set as missing. A-specific/polymorphic, non-autosomal and <95% success rate probes were removed. The measurement success rate per sample was >99%. Methylation data was normalised using noob and Functional Normalization (5 principal components) from the minfi package. Batch effects were corrected using ComBat [35].

**Funding**

This work was supported by the Netherlands Heart Foundation (2002B027 and 2006B083), The Corporate Development International (2005), The Netherlands Organization for Scientific Research NWO (911-03-016) and the EU funded Network of Excellence LifeSpan (FP6 036894). The funders had no role in study design, data collection and analysis, decision to publish, or preparation of the manuscript.

**Acknowledgements**

We are grateful to the women who made this work possible. Furthermore, we thank Mr. B.D. van Zelst and Mr. P.H. Griffioen for laboratory assistance, and the project team of the HAVEN Study, Dr A. Verkleij-Hagoort, Mrs L. van Driel, Mrs H. Smedts and Dr M. Wildhagen, for data management and data collection.

1. **Healthy start**

**Description of cohort**

The Healthy Start study is a prospective cohort which recruited ethnically diverse pregnant women from prenatal obstetrics clinics at the University of Colorado Hospital in 2010–2014 to study early life risk factors for diabetes and obesity [55,56]. Data on demographic characteristics, personal and family medical histories and behaviours during pregnancy were collected through questionnaires during pre- and post-natal research visits. Mothers were followed up through delivery and offspring are currently being followed through ages 8-11 years. Participants gave written informed consent, and the Colorado Multiple Institutional Review Board approved the study protocols.

**Definition of covariates**

Analyses were run separately for participants who self-described as non-Hispanic White and for those who self-described as Hispanic ethnicity. Maternal age at delivery, education and smoking during pregnancy were obtained from questionnaires administered at enrolment or during pregnancy. Maternal smoking during pregnancy was defined as no smoking or any smoking during pregnancy. Maternal education was categorised into i) graduate degree (Masters, PhD), ii) 4 years of college, iii) Some college or associate degree, iv) High school degree or GED and v) Less than 12^th^ grade. New-born’s sex was extracted from the medical records at delivery.  Gestational age at birth was based on an estimated conception date created by averaging ≤4 recorded gestational-age estimates during pregnancy. These estimates were either reported by the participant at research visits or recorded by the provider on the prenatal medical record. Proportions of six cord white blood cell types (CD4+ T-lymphocytes, CD8+ T-lymphocytes, NK (natural killer) cells, B-lymphocytes, monocytes and granulocytes) were computed using the estimateCellCounts function in the *minfi* package [11].

**Generation and pre-processing methods of DNA methylation data**

Cord blood was collected at the time of delivery. Methylation analysis of cord blood samples was conducted using the Illumina Infinium HumanMethylation450 BeadChip and processed in the University of Colorado Genomics Core lab. Samples with a mismatch between predicted and reported child sex were excluded, so were probes with high detection p-value (>0.05) or low bead count (<3 in at least 5% of the samples). The preprocessQuantile function in the R package Minfi was used for normalization [11]. Cleaned and normalised data were corrected for batch effects using ComBat [35].

**Funding**

The Healthy Start study was supported by the following grants from the National Institutes of Health: R01DK076648 (PI: Dabelea), R01ES022934 (MPI: Hamman, Adgate, Dabelea), UH3OD023248 (PI: Dabelea).

1. **HELIX**

**Description of cohort**

The Human Early Life Exposome (HELIX; <https://www.projecthelix.eu> ) study is a collaborative project across six established and ongoing longitudinal population-based cohort studies in Europe: the Born in Bradford (BiB) study in the UK, the Étude des Déterminants pré et postnatals du développement et de la santé de l’Enfant (EDEN) study in France, the INfancia y Medio Ambiente (INMA, Sabadell subcohort) project in Spain, the Kaunus cohort (KANC) in Lithuania, the Norwegian Mother, Father and Child Cohort Study (MoBa), and the Rhea Mother Child Cohort study in Crete, Greece [57]. The aim of HELIX is to investigate the effect of the exposome on health and the underlying molecular mechanisms. A subset of the HELIX consisting of BiB, EDEN and KANC (n=401) contributed data to the current study. Non-European ancestry children were excluded from the current analyses.

**Definition of covariates**

Maternal age at delivery, socio-economic status (SES) and smoking during pregnancy were obtained from maternal reported questionnaires assessed during pregnancy or after delivery. Maternal smoking during pregnancy was defined as no smoking, stopped smoking early in pregnancy or smoked throughout pregnancy. Parental education level was categorized as low (primary school), middle (secondary school) and high (university degree or higher). New-born’s gender and gestational age at delivery were extracted from medical records. Proportion of six cord white blood cell types (CD4+ T-lymphocytes, CD8+ T-lymphocytes, NK (natural killer) cells, B-lymphocytes, monocytes and granulocytes) were computed the Houseman method [4] using the Reinius reference panel [6].

**Generation of methylation data and pre-processing methods**

DNA, collected from buffy coat in EDTA tubes at age 7-9 years, was extracted using the Chemagen kit (Perkin Elmer) in batches of 12 samples by the individual cohorts. DNA concentration was determined in a NanoDrop 1000 UV-Vis Spectrophotometer (ThermoScientific) and with Quant-iT™ PicoGreen® dsDNA Assay Kit (Life Technologies). DNA methylation was assessed with the Infinium HumanMethylatio450 beadchip (Illumina, USA) following manufacturer’s protocol at the University of Santiago de Compostela – Spanish National Genotyping Center (CeGen-USC, Spain). Bisulfite conversion of DNA was carried out using the EZ 96-DNA kit (Zymo Research, USA) following the manufacturer’s standard protocol. All samples in the study were randomized considering sex, cohort, and panel (samples from the same child collected at different time points). In addition, each plate contained a HapMap control sample. A total of 24 HELIX inter-plate duplicates were included.

After an initial inspection of the quality of the methylation data with the MethylAid package [53], probes with a call rate <95% based on a detection p-value of 1E-16 [50]. Samples were removed i) if they were with a call rate <98%, ii) if their predicted sex was discordant to the reported sex and iii) if they were duplicates or related samples as verified by genotypic data from GWAS wherever available. Methylation data was normalized using the functional normalization method with prior background correction with Noob [16].

**Funding**

The research leading to these results has received funding from the European Community’s Seventh Framework Programme (FP7/2007-206) under grant agreement no 308333—the HELIX project. We acknowledge support from the Spanish Ministry of Science, Innovation and Universities through the “Centro de Excelencia Severo Ochoa 2019-2023” Program (CEX2018-000806-S), and support from the Generalitat de Catalunya through the CERCA Program.

**Born in Bradford**: supported by the National Institute for Health Research Applied Research Collaboration for Yorkshire and Humber (NIHR200166) and the Wellcome Trust (WT101597MA).

**The Norwegian Mother and Child Cohort Study (MoBa):** supported by the Norwegian Ministry of Health and the Ministry of Education and Research, NIH/NIEHS (contract no. N01-ES-75558), and NIH/NINDS (grant no. 1 UO1 NS 047537-01 and grant no. 2 UO1 NS 047537-06A1).

**The Rhea project:** financially supported by European projects, and the Greek Ministry of Health (Program of Prevention of Obesity and Neurodevelopmental Disorders in Preschool Children, in Heraklion district, Crete, Greece: 2011–2014; 'Rhea Plus': Primary Prevention Program of Environmental Risk Factors for Reproductive Health, and Child Health: 2012–2015). The work was also supported by MICINN (MTM2015-68140-R) and Centro Nacional de Genotipado-CEGEN-PRB2-ISCIII.

**The KANC work***:* supported in part by the European Commission grant number FP6-036224 and the grant of the Lithuanian Agency for Science Innovation and Technology (MITA), number 2012-07-20 no. 31V-125.

**Acknowledgements**

The authors would like to thank

- all the participating children, parents, practitioners, and researchers in the six countries who took part in this study.
- Sonia Brishoual, Angelique Serre and Michele Grosdenier (Poitiers Biobank, CRB BB-0033-00068, Poitiers, France) for biological sample management and Professor Frederic Millot (Principal Investigator)
- Elodie Migault, Manuela Boue and Sandy Bertin (Clinical Investigation Center, Inserm CIC1402, CHU de Poitiers, Poitiers, France) for planning and investigational actions.
- Veronique Ferrand-Rigalleau, Céline Leger and Noella Gorry (CHU de Poitiers, Poitiers, France) for administrative assistance (EDEN).
- Silvia Fochs, Nuria Pey, Cecilia Persavente and Susana Gros for field work, sample management and overall management in INMA.
- Georgia Chalkiadaki and Danai Feida for biological sample management, to Eirini Michalaki, Mariza Kampouri, Anny Kyriklaki and Minas Iakovidis for field study performance and to Maria Fasoulaki for administrative assistance (Rhea).
- Ingvild Essen for thorough field work, Heidi Marie Nordheim for biological sample management and the MoBa administrative unit (MoBa).
- all the Kaunas (KANC) cohort children and their parents for their cooperation, and all the field workers and laboratory personnel involved for their efforts.

**18. INMA**

**Description of cohort**

INfancia y Medio Ambiente (Environment and Childhood) study (INMA, <http://www.proyectoinma.org/>) is a collection of birth cohorts in Spain whose aim is to study the effect of pre- and postnatal environmental exposures (air pollutants, water and diet during pregnancy) on growth, health, and development starting from early foetal life to adolescence [58]. The study enrolled pregnant women (12 weeks of pregnancy) at public primary health care centres or public hospitals between 1997 and 2008. Data on mothers and their children were collected through questionnaires, face-to-face interviews, clinical data, physical examination, and ultrasound measurements. Biological samples were also collected. All participants gave Informed consent. The Hospital Ethics Committees in each participating region reviewed and approved the study. The current study included methylation data assessed at birth and at age 4 years from one of the INMA sub-cohorts: INMA-Sabadell.

**Definition of covariates**

Maternal age at delivery and socio-economic status (SES) were obtained from questionnaires. Maternal SES level was categorized into three groups based on maternal occupation: low (semi-skilled/unskilled occupations), medium (skilled manual/non-manual) or high (managers/technicians). Pregnant women were asked whether they were current smokers at week 32 of their pregnancy. If women had reported stopping smoking due to pregnancy, then they were asked if they stopped smoking before pregnancy or at what month of pregnancy. Smoking was defined as i) no smoking in pregnancy, ii) stopped in early pregnancy or iii) smoked throughout pregnancy. New-born’s gender was extracted from medical records. Gestational age at birth was estimated from the self-reported date of the last menstrual period confirmed by the first ultrasound examination (about 12th week of gestation). When the difference between the LMP reported at recruitment and estimated from the ultrasound was ≥7 days, we estimated LMP using the crown-rump length [59]. Age when blood samples were collected, and season of collection were also included as covariates in the age 4 analyses. Proportions of six cord white blood cell types (CD4+ T-lymphocytes, CD8+ T-lymphocytes, NK (natural killer) cells, B-lymphocytes, monocytes and granulocytes) were computed by applying the Houseman method [4] using Bakulski’s [5] and Reinius’ [6] reference panels for cord and child blood samples respectively. In addition, the nucleated erythrocyte fraction (nRBC) was also estimated for cord blood samples. The cell type proportions were included as covariates in the EWAS models. Covariate data were available for 342 new-borns and 200 children aged 4 years for the current analyses.

**Generation of methylation data and pre-processing methods**

DNA from cord (at birth) and whole blood (age 4) samples was extracted using the Chemagen kit (Perkin Elmer). DNA concentration was determined by a NanoDrop spectrophotometer (Thermo Scientific) and with the Quant-iT PicoGreen dsDNA Assay Kit (Life Technologies). Blood methylation data were produced in two laboratories: the Genome Analysis Facility of the University Medical Center Groningen (UMCG) in Holland as part of the MeDALL project (cord and 4-year blood samples), and the Bellvitge Biomedical Research Institute (IDIBELL) in Barcelona as part of the BREATHE project (cord blood). Both laboratories randomized samples in batches before plating onto 96-well plates. Samples were assayed for methylation levels using Infinium HumanMethylation450 BeadChip following the Illumina protocol. Briefly, DNA was subjected to bisulfite conversion using the EZ 96-DNA methylation kit, and DNA methylation was measured through hybridization on the BeadChips. BeadChips were scanned with an Illumina iScan, and image data were uploaded into the Methylation Module of Illumina’s analysis software GenomeStudio to convert them into β-values.

Samples with overall low quality as identified by MethylAid package [53] (n=2) and samples discordant for sex (shinyMethyl package) [16] (n=3) were removed. In addition, samples with a call rate <98% that did not pass a stringent detection p-value of 1.10E-16 [50] (n=18) were also excluded. Furthermore, 7,136 probes with a call rate <95%, control probes and SNP probes designed to detect genetic polymorphisms were excluded from further analyses. Data were normalized with the functional normalization method with prior background correction with Noob implemented in the minfi package [11]. Data were corrected for batch effects using ComBat [35].

**Funding**

The main funding of the epigenetic studies in INMA was through grants from the Instituto de Salud Carlos III (Red INMA G03/176, CB06/02/0041), the Spanish Ministry of Health (FIS-PI04/1436, FIS-PI08/1151 including FEDER funds, FIS-PI11/00610, FIS-FEDER-PI06/0867, FIS-FEDER-PI03-1615) the Generalitat de Catalunya-CIRIT 1999SGR 00241, the Fundació La marató de TV3 (090430), the EU Commission (261357-MeDALL: Mechanisms of the Development of ALLergy), and the European Research Council (268479-BREATHE: BRain dEvelopment and Air polluTion ultrafine particles in scHool childrEn). Lucas.A.Salas. is supported by CDMRP/Department of Defense (W81XWH-20-1-0778) and NIGMS (P20GM104416-09/8299).

**Acknowledgements**

INMA researchers would like to thank all the participants for their generous collaboration. A full roster of the INMA Project Investigators can be found at http://www.proyectoinma.org/presentacioninma/listado-investigadores/en_listado-investigadores.html.

1. **IoW (F1)**

**Description of cohort**

The Isle of Wight Birth Cohort (IOW F1) is a population birth cohort established on the Isle of Wight, UK, in 1989 to prospectively study the natural history of allergic diseases of newborns up to adulthood [60,61]. Pregnant mothers (n=1536) whose expected date of delivery was between 1 January 1989 and 28 February 1990 were enrolled into the study. Written informed consent was obtained from the parents to enroll 1456 newborns. Children have been followed up at the ages of 1, 2, 4, 10, 18 years and 26 years. Epigenome-wide DNA methylation has been measured in blood derived DNA of participants at age 10, 18 and 26 years and in perinatal blood DNA from Guthrie cards. Ethics approvals were obtained from the Isle of Wight Local Research Ethics Committee (now named the National Research Ethics Service, NRES Committee South Central – Southampton B) at recruitment and for the 1, 2, 4, 10, 18 and 26-year follow-ups.

**Definition of covariates**

Maternal age at delivery was derived from the mother’s date of birth. Maternal socioeconomic status (SES) and smoking during pregnancy was derived from questionnaires administered before and during pregnancy. Maternal smoking status during pregnancy was categorized into two groups: never smoked or smoked throughout pregnancy. Maternal SES was defined using maternal socioeconomic (income-education) cluster information [high-medium, medium-(low to medium), (low to medium)-high, low-(low to medium) and low-low]. Gestational age and the newborn’s gender were collected from stored clinical information. Proportion of six white blood cell types from whole blood samples (CD4+ T-lymphocytes, CD8+ T-lymphocytes, NK (natural killer) cells, B-lymphocytes, monocytes and granulocytes) were computed by applying the reference-based Houseman method [4] using the adult reference panel developed by Reinius *et al* [6] using minfi [11]. The study population was >99% Caucasian.

**Generation and pre-processing methods of DNA methylation data**

Genomic DNA, extracted from blood samples of children aged 10 years, were subjected to bisulfite conversion using the EZ-96 DNA Methylation kit (Shallow) (Zymo Research Corporation, Irvine, USA). Samples were plated onto 96-well plates in a random order and processed with the Illumina Infinium HumanMethylation450 BeadChip (Illumina Inc., San Diego, USA) or Illumina Infinium HumanMethylation EPIC Beadchip.

Methylation data were quality controlled and normalised using the CPACOR pipeline [50]. Methylation markers on 65 single nucleotide polymorphism (SNP) and sex chromosomes were removed. Samples with call rate <98% were excluded from the analysis and probes having detection p-values ≥ 10^-16^ were set as missing data. A quantile normalisation was applied using limma on intensity values of the remaining probes separately based on six different probe-type categories (Type-I M red, Type-I U red, Type-I M green, Type-I U green, Type-II red, and Type-II green). Beta values were then calculated from the normalised intensity values. The R package ComBat [35] built upon an empirical Bayes framework was used to remove batch and platform effects with ‘batch’ included as a covariate in the model. Probes common between 450k and EPIC beadchips were only used in the meta-analysis. The final dataset, therefore, contained methylation data on 418,857 probes for 124 samples (collected at age 10) with complete covariate information.

**Funding**

This work has been supported by National Institute of Health R01 AI091905 and R01HL132321, R01 AI121226, R01 HL082925; National Asthma Campaign, UK (Grant No 364). The work of Hongmei Zhang and John Holloway is also supported by the fund from NIAID/NIH (R01AI121226, MPI: Hongmei Zhang and John Holloway). IoW was also supported by the Medical Research Council UK (MR/S025340/1). Md Zahangir Alam was a Commonwealth Scholar, funded by the UK government.

**Disclaimer**

The content is solely the responsibility of the authors and does not necessarily represent the official views of the National Institutes of Health, USA.

**Acknowledgements**

We greatly appreciate the participating families in the IOW F1 cohort study. IOW Researchers are grateful to Stephen Potter for data processing and Nikki Graham for technical support and other members of the IOW research group for valuable discussion. We thank the High-Throughput Genomics Group at the Wellcome Trust Centre for Human Genetics for the generation of the methylation data.

1. **IOW F2**

**Description of cohort**

The Isle of Wight 3^rd^ Generation Cohort (IoW F2) is an ongoing study which recruited 600 newborns of IOW F1 parents from 2010 onwards. Epigenome-wide DNA methylation was measured for 193 children using DNA extracted from cord blood. The study population was >99% Caucasian. For permission was obtained from the National Research Ethics Service Committee South Central - Hampshire B (09/H0504/129) approved the recruitment of the third generation (F2) during pregnancy. The committee also gave permission to obtain informed consent of mothers and fathers for their assessment and follow-up of their offspring at 3, 6 and 12 months. Approval was granted for further assessments of F2-children at 2 years (REC no.14/SC/0133), 3 years (REC no. 14/SC/1191) and 6–7 years (REC no. 17/EM/0083.)

**Definition of covariates**

Maternal age at delivery was derived from the mother’s date of birth. Maternal socioeconomic status (SES) and smoking during pregnancy was derived from questionnaires administered before and during pregnancy. Maternal smoking status during pregnancy (Yes/No) was defined as any smoking during pregnancy or never smoked. Maternal SES was grouped into four groups using cluster analysis using maternal education: 1) left before general certificate of secondary education, 2) completed education at 16 years, 3) completed education at 18 years, and 4) Other, e.g., vocational training. Gestational age and the newborn’s sex were collected from stored clinical information. Proportion of seven cord white blood cell types (CD4+ T-lymphocytes, CD8+ T-lymphocytes, NK (natural killer) cells, B-lymphocytes, monocytes, granulocytes nucleated red blood cells- nRBC) were computed by applying the reference-based Houseman method [4] using the cord blood reference panel developed by Bakulski *et al* [5] in minfi [11].

**Generation and pre-processing methods of DNA methylation data**

Genomic DNA extracted from cord blood samples was subjected to bisulfite conversion using the EZ-96 DNA Methylation kit (Shallow) (Zymo Research Corporation, Irvine, USA). Samples were plated onto 96-well plates in a random order and processed with the Illumina Infinium HumanMethylation450 BeadChip (Illumina Inc., San Diego, USA) or Illumina Infinium HumanMethylation EPIC. Of the 193 cord blood samples thus assess, 130 were processed using 450k and 63 by EPIC. One of the twins, selected at random, was excluded from the analysis, resulting in data from 192 samples for further pre-processing.

Methylation data were pre-processed using the Bioconductor IMA package (Illumina methylation analyzer). CPACOR [50] pipeline was used for QC and normalisation of the data. Methylation markers on 65 single nucleotide polymorphism (SNP) and sex chromosomes were removed. Samples with call rate< 98% were excluded from further analyses. Probes having detection p-values ≥10^-16^ were set as missing. A quantile normalisation was applied using limma on intensity values of the remaining probes separately based on six different probe-type categories (Type-I M red, Type-I U red, Type-I M green, Type-I U green, Type-II red, and Type-II green). Beta values were then calculated from the normalised intensity values. The R package ComBat [35], built upon an empirical Bayes framework, was used to remove batch and platform effects with ‘batch’ included as a covariate in the model. Probes common between 450k and EPIC beadchips were only used in the meta-analysis. The final dataset, therefore, contained methylation data on 399,383 probes for 185 samples with complete covariate information.

**Funding**

The third-generation study was funded by the National Institute of Allergy and Infectious Diseases (NIAID) at the National Institute of Health, R01 AI091905 (PI: Wilfried Karmaus). The work of Hongmei Zhang and John Holloway is also supported by funds from NIAID/NIH (R01AI121226, MPI: Hongmei Zhang and John Holloway).

**Disclaimer**

The content is solely the responsibility of the authors and does not necessarily represent the official views of the National Institutes of Health, USA.

**Acknowledgements**

We would like to thank all the participants of the Isle of Wight 3rd Generation birth cohort and their parents (IOW F1), the research team at David Hide Asthma & Allergy Research Centre (Isle of Wight) for collecting the data, Stephen Potter for data management, Nikki Graham for technical support and other members of the IOW research group for valuable discussion. We thank the High-Throughput Genomics Group at the Welcome Trust Centre for Human Genetics for the generation of the methylation data.

1. **LiNA**

**Design and study population**

LiNA (Lifestyle and environmental factors and their Influence on New-borns Allergy risk) is a birth cohort study which recruited 629 mother–child-pairs between May 2006 and December 2008 in Leipzig, Germany, to investigate how environmental factors in the pre- and postnatal period influence immune system development and resulting disease risks [62,63]. Briefly, Blood samples were obtained during pregnancy (mother, 34th week of gestation), at birth (venous umbilical cord blood) and every year thereafter during annual follow ups. Data on confounding variables, prenatal exposure, lifestyle factors and children's disease outcomes were obtained from questionnaires filled in by the parents four weeks before birth and on the subsequent annual follow ups. Informed consent was obtained from the parents. The study was approved by the Ethics Committees of the University of Leipzig and the Saxonian Board of Physicians (file reference 046‐2006, 160-2008, 160b/2008, 144-10-31052010, 113-11-18042011, 206-12-02072012, 169/13-ff, 150/14-ff, EK-allg-28/14-1).

**Definition of covariates**

Maternal age at delivery, gestational age and child sex were collected from maternity log (official booklet kept by the mothers) and inserted into a structured questionnaire by a physician shortly after birth. Maternal smoking during pregnancy was based on self-administered questionnaires during pregnancy (at 36 weeks). Smoking was categorised into three groups: never smoked during pregnancy, stopped during early pregnancy, and smoked throughout pregnancy. Maternal socioeconomic status was defined by maternal education according to ISCED 1997. Proportions of seven cord white blood cell types (CD4+ T-lymphocytes, CD8+ T-lymphocytes, NK (natural killer) cells, B-lymphocytes, monocytes, granulocytes and nucleated red blood cells (nRBC)) were computed by applying the Houseman method [4] using Bakulski’s reference panels for cord blood samples [5] and were included as covariates in EWAS analyses. In addition, six principal components of the normalised data were included in the regression models to correct for batch effects.

**Generation of methylation data and pre-processing methods**

Details of the DNA methylation measurements and quality control for the LiNA participants were previously described [64]. Briefly, genomic DNA was extracted from cord blood samples using the QIAmp DNA Blood Mini Kit (Qiagen, Hilden, Germany). Extracted DNA was subjected to bisulfite conversion using the EZ-96 DNA Methylation kit (Zymo Research Corporation, Orange, USA) according to manufacturer recommendations. DNA methylation was measured using Illumina’s Infinium HumanMethylation450 BeadChip (Illumina, Sandego, USA). All samples subsequently subjected to DNA methylation analyses (n = 472) passed initial quality control checks. DNA methylation values, described as beta (β) values, were recorded for each locus in each sample. The β values represent the ratio of methylated signal relative to the sum of methylated and unmethylated signal measured per CpG. Data were normalized using the SWAN (subset-quantile within array normalization) method of the minfi R package. Probes on X and Y chromosomes as well as control probes were excluded. Normalized data were corrected for batch effects by including six principal components as covariates in the EWAS analyses.

**Funding**

The core funding for the LINA study is provided by the Helmholtz Center for Environmental Research –UFZ / Department of Environmental Immunology. The methylation analysis component of the LINA study was supported by the German Cancer Research Centre – DKFZ.

**Acknowledgements**

We thank Melanie Bänsch, Anne Hain, Beate Fink, and Michaela Loschinski for their excellent technical assistance and field work. Furthermore, we cordially thank the LiNA children and their families for their ongoing participation, and our clinical cooperation partners.

**22, 23. MoBA1 and MoBA2**

**Design and study population**

The Norwegian Mother, Father and Child Cohort Study (MoBa) is a prospective population-based pregnancy cohort study conducted by the Norwegian Institute of Public Health (<https://www.fhi.no/en/studies/moba/>). MoBa aimed to study association between specific exposures and health outcomes, specifically amongst child participant but also amongst their parents. All child participants of MoBa study were born between 1999-2009 [65]. Participants represent two subsets of mother-offspring pairs of the MoBa study referred to as MoBa1 and MoBa2. MoBa1 and MoBa2 participated in the current study by providing DNA methylation data of cord blood samples from the new-borns. Written informed consent was obtained from all participating mothers of MoBa. The establishment and data collection in MoBa has obtained a license from the Norwegian Data Inspectorate and approval from The Regional Committee for Medical Research Ethics. All studies were approved by the Regional Committee for Ethics in Medical Research, Norway. In addition, they were also approved by the Institutional Review Board of the National Institute of Environmental Health Sciences, USA.

The consent given by the participants does not allow for storage of data on an individual level in repositories or journals. Researchers who want access to data sets for replication should apply to [datatilgang@fhi.no](mailto:datatilgang@fhi.no). Access to data sets requires approval from The Regional Committee for Medical Research Ethics in Norway and a formal contract with MoBa.

**Definition of covariates**

Maternal age at delivery, child sex and gestational age were recorded from the Norwegian Medical Birth Registry. Maternal smoking during pregnancy and socioeconomic status (education) was based on maternal self-reported questionnaire data. Smoking was categorised into three groups: never smoked during pregnancy, stopped in pregnancy, and smoked heavily during pregnancy. Maternal education was categorised as less than high school, high school, some college education, and more than 4 years of college education. Proportion of six cord white blood cell types (CD4+ T-lymphocytes, CD8+ T-lymphocytes, NK (natural killer) cells, B-lymphocytes, monocytes and granulocytes) were computed by applying the Houseman method [4] using Bakulski’s reference panels for cord blood samples [5].

**Generation of methylation data and pre-processing methods**

Details of the DNA methylation measurements and quality control for the MoBa1 participants were previously described and the same protocol was implemented for the MoBa2 participants [66]. Briefly, umbilical cord blood samples were collected and frozen at birth at -80°C. DNA extracted from cord blood was subjected to bisulfite conversion using the EZ-96 DNA Methylation kit (Zymo Research Corporation, Irvine, CA) and DNA methylation was measured at 485,577 CpGs in cord blood using Illumina’s Infinium HumanMethylation450 BeadChip. Raw intensity (.idat) files were handled in R using the minfi package to calculate the methylation level at each CpG as the beta-values. MoBa1 and MoBa2 datasets were analysed separately. Similar protocols were applied to MoBa1 and Moba2 for probe and sample-specific quality control. Briefly, control probes and probes on X and Y chromosomes were excluded in both datasets. Samples were removed if i) they failed as indicated by Illumina ii) they have an average detection p value across all probes ≥0.05, or iii) they were outliers identified in principal component analysis. Probes missing in >10% of the samples were also removed. Bias arising from the two different probe designs were accounted for by applying the intra-array normalization strategy Beta Mixture Quantile normalization (BMIQ) [17]. Normalized data were corrected for batch effects using ComBat [35]. Finally, samples with missing covariates were excluded from further analysis.

**Funding**

The Norwegian Mother, Father and Child Cohort Study is supported by the Norwegian Ministry of Health and Care Services and the Ministry of Education and Research, NIH/NIEHS (contract no N01-ES-75558), NIH/NINDS (grant no.1 UO1 NS 047537-01 and grant no.2 UO1 NS 047537-06A1). For this work, MoBa 1 and 2 were supported by the Intramural Research Program of the NIH, National Institute of Environmental Health Sciences (Z01-ES-49019) and the Norwegian Research Council/BIOBANK (grant no 221097). This work was partly supported by the Research Council of Norway through its Centres of Excellence funding scheme, project number 262700.

**Acknowledgements**

The Norwegian Mother, Father and Child Cohort Study is supported by the Norwegian Ministry of Health and Care Services and the Ministry of Education and Research. We are grateful to all the participating families in Norway who take part in this on-going cohort study.

**24. NEST**

**Design and study population**

NEST (New-born Epigenetics STudy) is a multi-ethnic prospective study of women and their children designed to identify the association between early exposures and changes in infantile epigenetic profile that may influence chronic disease susceptibility later in their lives [67]. Pregnant women who were 18 years or older, English speaking and who intended to use obstetrics facilities within the Duke Obstetrics or Durham Regional Hospitals as their prenatal clinics were recruited to the study between April 2005 to July 2009. Gestational age at enrolment ranged from 6 to 42 weeks (median 30 weeks). Women who were current smokers during their pregnancy were specially targeted. Of the 1101 women who met eligibility criteria, 895 (81%) were enrolled. Umbilical cord blood was collected from 741 infants born to the recruited mothers. Written informed consent was obtained from all participants. The Duke Institutional Review Board approved the study. The current analysis was limited to 413 infants with covariate and 450k methylation data.

**Definition of covariates**

Data on maternal age and gestational age at delivery and child sex were collected from the medical records at delivery. Information on maternal smoking during pregnancy and socioeconomic status (education) was based on self-reported questionnaire data. Smoking during pregnancy was categorised into three groups: never smoked during pregnancy, stopped in pregnancy, and smoked throughout pregnancy. Maternal education was defined as high school education or less, some college, and college degree or higher. Proportion of seven cord blood cell types (nRBC, CD8T, CD4T, NK (natural killer) cells, B cells, monocytes and granulocytes) were computed by applying the Houseman method [4] using Bakulski’s reference panels for cord blood samples [5]. These were included as covariates in the EWAS models.

**Generation of methylation data and pre-processing methods**

Genomic DNA from buffy coat specimens was extracted from umbilical cord blood using Puregene Reagents (Qiagen, Valencia, CA). Extracted DNA was subjected to bisulfite conversion using the EZ-96 DNA Methylation Kit (Zymo Research Corporation) and DNA methylation was measured at 485,577 CpGs using Illumina Infinium HumanMethylation450 BeadChip (Illumina Inc., San Diego, USA. Methylation levels at each CpG (beta values) were calculated using Illumina’s GenomeStudio Methylation module version 1.0 (Illumina Inc.). Probe and sample-specific quality control was performed in the NEST cohort using a similar approach to MoBa1 and MoBa2 cohorts as the data analysis was completed at the NIEHS. Specifically, control probes (n=65) and probes on X (n=11 230) and Y (n=416) chromosomes were excluded as well as CpGs missing >10% of methylation data. Samples were removed if i) indicated to have failed by Illumina pre-processing, ii) the average detection p-value across all probes were <0.05 or iii) there was discordance between the reported sex and predicted sex. Bias arising from the two different probe designs were corrected by applying the intra-array normalization strategy Beta Mixture Quantile dilation (BMIQ) [17]. The Empirical Bayes method via *ComBat* was applied for batch correction using the *sva* package in *R* [35].

**Funding**

The NEST study was funded by NIEHS grants R21ES014947 and R01ES016772 and NIDDK grant R01DK085173. Cathrine Hoyo and Dereje D Jima have received funding from the National Institute of Environmental Health Science (P30 ES025128). Cathrine Hoyo, Susan Murphy, and Rachel Maguire have received funding from the National Institute of Environmental Health Science (R24ES028531).

**Acknowledgements**

We thank the parents and other caregivers of the Newborn Epigenetics STudy. We also thank the field and laboratory staff for their effort.

**25. PREDO**

**Design and study population**

PREDO (Prediction and Prevention of Preeclampsia and Intrauterine Growth Restriction) is a longitudinal, multicentre, mother-children study of Finnish women and their singleton children born alive between 2006-2010. Details of the study design and inclusion/exclusion criteria are described elsewhere [68]. The PREDO study was set up to identify novel risk factors and biomarkers in pregnant women associated with the development of preeclampsia and intrauterine growth restriction. The study recruited 1079 pregnant women, of whom 969 had one or more and 110 had none of the known risk factors for preeclampsia and intrauterine growth restriction. All participants provided written informed consent. Consent of participating children were provided by parent(s)/guardian(s). The Ethics Committees of Obstetrics and Gynaecology, and Women, Children and Psychiatry of the Helsinki and Uusimaa Hospital District and of the participating hospitals approved the study protocol.

**Definition of covariates**

Data on mother’s age at childbirth, smoking during pregnancy, new-born sex and gestational age at birth were extracted from the Finnish Medical Birth Register and/or hospital records. Smoking during pregnancy was categorised into three groups: never smoked during pregnancy, stopped in pregnancy, and smoked throughout pregnancy. Self-reported information (reported during early pregnancy) on maternal education was used to categorise the maternal social status into four groups - primary education, secondary education, lower tertiary education, and upper tertiary education. Proportions of the six cord white blood cell types (CD4+ T-lymphocytes, CD8+ T-lymphocytes, NK (natural killer) cells, B-lymphocytes, monocytes and granulocytes) were computed by applying the Houseman method [4] and the Bakulski reference panel [5] using the estimateCellCounts function of the Minfi package [11] in R. These were included as covariates in the EWAS models.

**Generation of methylation data and pre-processing methods**

DNA from cord blood samples was extracted according to standard procedure. To limit batch effects, samples were randomized based on gender and maternal risk factors for pre-eclampsia before plating onto the 96-well plates. Bisulfite conversion was performed using the EZ-96 DNA methylation kit (Zymo research Corporation, Irvine, USA). Methylation levels at 485,000 CpG sites were measured as beta values (0 = no methylation and 1 = complete methylation) using the Infinium HumanMethylation450 BeadChip (Illumina Inc., San Diego, USA). Pre-processing and quality control of the raw data was carried out using the R-package *minfi [*11]. Samples were excluded if i) they were duplicated, ii) they were outliers in the median intensities, iii) there were mismatches in the reported and predicted sex or iv) contaminated with maternal DNA. Probes on the sex chromosomes, cross-hybridizing probes (as described in [13,69]), probes containing SNPs, and probes with a detection *P*-value >0.01 in at least 25% of the samples were excluded. Methylation beta-values were normalized using the funnorm function of *minfi* [11]. Principal component analyses carried out on the normalized beta values identified two batch-related factors, slide and well, that contributed to most of the variance in the methylation levels. These batch effects were removed iteratively using the *ComBat* software [35].

**Funding**

The PREDO study received funding from the Academy of Finland, EraNet, EVO (a special state subsidy for health science research), University of Helsinki Research Funds, the Signe and Ane Gyllenberg foundation, the Emil Aaltonen Foundation, the Finnish Medical Foundation, the Jane and Aatos Erkko Foundation, the Novo Nordisk Foundation, the Juho Vainio foundation, the Yrjö Jahnsson foundation, the Jalmari and Rauha Ahokas foundation, the Paivikki and Sakari Sohlberg Foundation, the Sigrid Juselius Foundation, and the Sir Jules Thorn Charitable Trust**.**

**Acknowledgements**

The PREDO study would not have been possible without the dedicated contribution of the PREDO study group members: E Hamäläinen, E Kajantie, H Laivuori, PM Villa, A-K Pesonen, A Aitokallio-Tallberg, A-M Henry, VK Hiilesmaa, T Karipohja, R Meri, S Sainio, T Saisto, S Suomalainen-Konig, V-M Ulander, T Vaitilo (Department of Obstetrics and Gynaecology, University of Helsinki and Helsinki University Central Hospital, Helsinki, Finland), L Keski-Nisula, Maija-Riitta Orden (Kuopio University Hospital, Kuopio Finland), E Koistinen, T Walle, R Solja (Northern Karelia Central Hospital, Joensuu, Finland), M Kurkinen (Päijät-Häme Central Hospital, Lahti, Finland), P.Taipale. P Staven (Iisalmi Hospital, Iisalmi, Finland), J Uotila (Tampere University Hospital, Tampere, Finland). We thank all the PREDO children and their parents for their enthusiastic participation. We also thank all the research nurses, research assistants, and laboratory personnel involved in the PREDO study.

**26. STOPPA**

**Description of cohort**

STOPPA (The Swedish Twin study On Prediction and Prevention of Asthma) is a twin cohort study of 752 individuals [70]. Study participants were selected from an ongoing data collection within the Child and Adolescent Twin study in Sweden (CATSS) based on the pair’s asthma status [71]. Approximately one third each of asthma concordant (ACC), asthma discordant (ADC) and healthy concordant (HCC) twin pairs took part in clinical examinations including questionnaires, lung function testing (spirometry with reversibility test and fractional exhaled nitric oxide, FeNO) and collection of bio-samples. The participants and their parents gave written informed consent. The regional ethical review board in Stockholm, Sweden approved the study. The twins were 9-14 years old at the time of invitation to the study. The current study utilised data from a subset of 9 to 11-year olds.

**Definition of covariates**

Data on gestational age, child’s sex, maternal age at first antenatal care and maternal smoking during pregnancy (any/never) were available from maternal antenatal and birth records. Child’s age was collected at the clinical examination. Maternal social class was assessed based on maternal education the data for which were collected through questionnaires. Maternal education was defined by the highest self-reported education level achieved by the mother at the time of clinical examination and was categorized as “Less than high school level”, “High school or equivalent degree”, “Some college or university studies” or “3+ years of college/university or a university degree.” Recruitment to STOPPA was based on asthma case/control status within twin pairs. The case/control variable for asthma status of the individuals was, therefore, included as a covariate in the regression models (cases: 141; controls: 95). Six (CD4+ T-lymphocytes, CD8+ T-lymphocytes, NK (natural killer) cells, B-lymphocytes, monocytes, and granulocytes) cell type proportions were included as covariates in the regression models. Batch effects were corrected by including the plate ID as a covariate in the models.

**Generation and pre-processing methods of DNA methylation data**

DNA was extracted from whole blood using the Chemagic Star 400 kit (PerkinElmer chemagen, Baesweiler, Aachen, Germany) according to a standardized protocol [70]. Samples other than those from twin pairs were plated randomly to minimise batch effects. Twin-pair samples were kept within the same chip to allow for within-pair comparisons free of batch effects. DNA methylation assays were carried out at the Mutation Analysis Facility (MAF), Karolinska Institutet, Stockholm, Sweden, using the Infinium HumanMethylation450 Beadchip Kit (Illumina, Inc., San Diego, California, USA). Quality control, sample and probe filtering were performed using *RnBeads* [72]. Predicted gender and phenotype-based sex were compared and matched for all samples. Probes were filtered out if they i) overlapped with single nucleotide polymorphisms or specific nucleotide contexts, ii) with unreliable measurements (defined as detection *P*-values > 5X10^-8^), iii) were located on sex chromosomes. Methylation at each CpG site was expressed as beta values. Beta values were normalized using the dasen method, which includes background adjustment and separate between-array normalization of Type I and Type II probes [14]. In order to obtain robust standard errors and to correct for within-cluster (i.e. within-pair) correlations, EWAS analyses of STOPPA data were carried out using a GEE (Generalized Estimating Equation) model, specifying twin pair numbers as the clustering variable, using the drgee R package [73]. Briefly, GEE produce robust standard errors corrected for the within-pair correlations. This method tends to result in slightly larger SEs compared to standard linear regression, but on the other hand avoids violating the assumptions of independence between observations. The beta values are generally unaffected.

**Funding**

Financial support was provided by the Swedish Research Council (grant no 2018-02640) and through the Swedish Initiative for research on Microdata in the Social And Medical Sciences (SIMSAM) framework grant number 340-2013-5867, grants provided by the Stockholm County Council (ALF projects), the Swedish Heart Lung Foundation, the Swedish Asthma and Allergy Association's Research Foundation and Stiftelsen Frimurare Barnhuset Stockholm.

**Acknowledgements**

First, we express our greatest appreciation to the twins and parents of the STOPPA cohort, without whose participation this study could not have been performed. We are also indebted to the STOPPA research nurses and database managers for their excellent data collection and data managing. We would also like to extend our thanks to the eight paediatric allergy clinics around Sweden for their great collaboration during our visits for clinical examinations. We acknowledge the Swedish Twin Registry for access to data. The Swedish Twin Registry is managed by Karolinska Institutet and receives funding through the Swedish Research Council under the grant no 2017-00641. We also acknowledge the Biobank at Karolinska Institutet for professional biobank service.

**27. Project Viva**

**Description of cohort**

Project Viva ((<https://www.hms.harvard.edu/viva/index.html>) is a prospective pre-birth cohort of mother-offspring pairs established to examine the effect of prenatal diet and other factors on the heath of the mother-offspring pairs. Pregnant women were recruited to the study between 1999 and 2002 from Atrius Harvard Vanguard Medical Associates, a multispecialty group practice in eastern Massachusetts. Details on recruitment and data collection have been published elsewhere [74]. All mothers in the study signed informed consents. The institutional review board of Harvard Pilgrim Health Care approved the study protocol. A total of 485 children with complete data on cord blood DNA methylation, outcomes and covariates were included in the current study.

**Definition of covariates**

Information on maternal age, pregnancy smoking status, social class, and child’s sex were collected via Project Viva questionnaires and interviews. Maternal smoking during pregnancy was categorised into two groups: smoked during pregnancy or never smoked/former smoker. Maternal social class was divided into two groups based on the mother’s education: graduated college or did not graduate college. Infant’s birth date was obtained from the hospital medical record. Gestational age at birth was calculated using the date of the last menstrual period. However, if the early second-trimester ultrasound assessment differed from the calculated gestational age by more than 10 days, the ultrasound dating was used for the calculation of gestational age. Cell types proportions in cord blood were estimated by applying the Houseman projection method [4] using a reference panel of nucleated cells isolated from cord blood (leukocytes and nucleated red blood cells) [5].

**Generation and pre-processing methods of DNA methylation data**

Trained medical personnel obtained umbilical cord blood samples immediately upon delivery, stored them in a dedicated refrigerator at 4°C and transported to a central location within 24 hours of sample collection. DNA was extracted by trained laboratory staff using Qiagen Puregene Kit (Valencia, CA) on the same day of arrival. Aliquots were then stored at −80 °C until analysis. DNA samples were plated following a stratified randomization procedure to ensure balance of cohort characteristics across sample plates/batches. Samples were subjected to bisulfite conversion using the EZ-96 DNA Methylation kit (Zymo Research Corporation, Irvine, USA). DNA methylation assays were carried out at the Illumina FastTrack Microarray Services (San Diego, CA) using the Illumina Infinium HumanMethylation450 BeadChip (Illumina Inc., San Diego, USA) [38]. Samples were removed if i) technical replicates, ii) of low quality, iii) mismatched genotypes or iv) mismatches between reported sex and predicted sex. Probes were excluded if they i) had non-significant detection p-values (>0.05) for more than 5% of the samples, ii) were non-CpG probes (i.e. rs and ch), iii) were probes on the sex chromosomes or iv) SNP-associated probes at either the single base extension or within the target region when a minor-allele frequency of the SNP was >5. Raw methylation values underwent background correction and dye-bias equalization via the normal-exponential out-of-band (noob) correction method. The resulting data were subjected to a β-mixture quantile intra sample normalization procedure (BMIQ) to minimise any bias that can arise from type2 probes. ComBat (R package) [35] was used to adjust for technical variabilities across sample plates while protecting ‘season of birth’ by making use of ComBat’s model statement.

**Funding**

Project Viva was supported by grants from the US National Institutes of Health (R01 HD034568, UH3 OD023286, R01 HL111108, R01 NR013945).

**Acknowledgements**

We thank the participants and staff of Project Viva.

**References**

1. Felix JF, Joubert BR, Baccarelli AA, *et al.* Cohort Profile: Pregnancy And Childhood Epigenetics (PACE) Consortium. *Int J Epidemiol*. 47(1), 22–23u (2018).

2. Boyd A, Golding J, Macleod J, *et al.* Cohort Profile: the ’children of the 90s’--the index offspring of the Avon Longitudinal Study of Parents and Children. *Int J Epidemiol*. 42(1), 111–127 (2013).

3. Fraser A, Macdonald-Wallis C, Tilling K, *et al.* Cohort Profile: the Avon Longitudinal Study of Parents and Children: ALSPAC mothers cohort. *Int J Epidemiol*. 42(1), 97–110 (2013).

4. Houseman EA, Accomando WP, Koestler DC, *et al.* DNA methylation arrays as surrogate measures of cell mixture distribution. *BMC Bioinformatics*. 13, 86 (2012).

5. Bakulski KM, Feinberg JI, Andrews SV, *et al.* DNA methylation of cord blood cell types: Applications for mixed cell birth studies. *Epigenetics*. 11(5), 354–362 (2016).

6. Reinius LE, Acevedo N, Joerink M, *et al.* Differential DNA methylation in purified human blood cells: implications for cell lineage and studies on disease susceptibility. *PLoS One*. 7(7), e41361 (2012).

7. Relton CL, Gaunt T, McArdle W, *et al.* Data Resource Profile: Accessible Resource for Integrated Epigenomic Studies (ARIES). *Int J Epidemiol*. 44(4), 1181–1190 (2015).

8. Min JL, Hemani G, Davey Smith G, Relton C, Suderman M. Meffil: efficient normalization and analysis of very large DNA methylation datasets. *Bioinformatics*. 34(23), 3983–3989 (2018).

9. Wickman M, Kull I, Pershagen G, Nordvall SL. The BAMSE project: presentation of a prospective longitudinal birth cohort study. *Pediatr Allergy Immunol*. 13(s15), 11–13 (2002).

10. Thacher JD, Gruzieva O, Pershagen G, *et al.* Pre- and postnatal exposure to parental smoking and allergic disease through adolescence. *Pediatrics*. 134(3), 428–434 (2014).

11. Aryee MJ, Jaffe AE, Corrada-Bravo H, *et al.* Minfi: a flexible and comprehensive Bioconductor package for the analysis of Infinium DNA methylation microarrays. *Bioinformatics*. 30(10), 1363–1369 (2014).

12. Wang AL, Gruzieva O, Qiu W, *et al.* DNA methylation is associated with inhaled corticosteroid response in persistent childhood asthmatics. *Clin Exp Allergy*. 49(9), 1225–1234 (2019).

13. Chen Y, Lemire M, Choufani S, *et al.* Discovery of cross-reactive probes and polymorphic CpGs in the Illumina Infinium HumanMethylation450 microarray. *Epigenetics*. 8(2), 203–209 (2013).

14. Pidsley R, Y Wong CC, Volta M, Lunnon K, Mill J, Schalkwyk LC. A data-driven approach to preprocessing Illumina 450K methylation array data. *BMC Genomics*. 14, 293 (2013).

15. Ma X, Buffler PA, Wiemels JL, *et al.* Ethnic difference in daycare attendance, early infections, and risk of childhood acute lymphoblastic leukemia. *Cancer Epidemiol Biomarkers Prev*. 14(8), 1928–1934 (2005).

16. Fortin J-P, Labbe A, Lemire M, *et al.* Functional normalization of 450k methylation array data improves replication in large cancer studies. *Genome Biol*. 15(12), 503 (2014).

17. Teschendorff AE, Marabita F, Lechner M, *et al.* A beta-mixture quantile normalization method for correcting probe design bias in Illumina Infinium 450 k DNA methylation data. *Bioinformatics*. 29(2), 189–196 (2013).

18. Eskenazi B, Bradman A, Gladstone EA, Jaramillo S, Birch K, Holland N. CHAMACOS, A Longitudinal Birth Cohort Study: Lessons from the Fields. *Journal of Children’s Health*. 1(1), 3–27 (2003).

19. Eskenazi B, Harley K, Bradman A, *et al.* Association of in utero organophosphate pesticide exposure and fetal growth and length of gestation in an agricultural population. *Environ Health Perspect*. 112(10), 1116–1124 (2004).

20. Yousefi P, Huen K, Aguilar Schall R, *et al.* Considerations for normalization of DNA methylation data by Illumina 450K BeadChip assay in population studies. *Epigenetics*. 8(11), 1141–1152 (2013).

21. Kirchberg FF, Harder U, Weber M, *et al.* Dietary protein intake affects amino acid and acylcarnitine metabolism in infants aged 6 months. *J Clin Endocrinol Metab*. 100(1), 149–158 (2015).

22. Koletzko B, von Kries R, Closa R, *et al.* Lower protein in infant formula is associated with lower weight up to age 2 y: a randomized clinical trial. *Am J Clin Nutr*. 89(6), 1836–1845 (2009).

23. Rzehak P, Saffery R, Reischl E, *et al.* Maternal Smoking during Pregnancy and DNA-Methylation in Children at Age 5.5 Years: Epigenome-Wide-Analysis in the European Childhood Obesity Project (CHOP)-Study. *PLoS One*. 11(5), e0155554 (2016).

24. Weber M, Grote V, Closa-Monasterolo R, *et al.* Lower protein content in infant formula reduces BMI and obesity risk at school age: follow-up of a randomized trial. *Am J Clin Nutr*. 99(5), 1041–1051 (2014).

25. Touleimat N, Tost J. Complete pipeline for Infinium(®) Human Methylation 450K BeadChip data processing using subset quantile normalization for accurate DNA methylation estimation. *Epigenomics*. 4(3), 325–341 (2012).

26. McConnell R, Berhane K, Yao L, *et al.* Traffic, susceptibility, and childhood asthma. *Environ Health Perspect*. 114(5), 766–772 (2006).

27. Noushmehr H, Weisenberger DJ, Diefes K, *et al.* Identification of a CpG island methylator phenotype that defines a distinct subgroup of glioma. *Cancer Cell*. 17(5), 510–522 (2010).

28. Triche TJ, Weisenberger DJ, Van Den Berg D, Laird PW, Siegmund KD. Low-level processing of Illumina Infinium DNA Methylation BeadArrays. *Nucleic Acids Res*. 41(7), e90 (2013).

29. Jaffe AE, Irizarry RA. Accounting for cellular heterogeneity is critical in epigenome-wide association studies. *Genome Biol*. 15(2), R31 (2014).

30. Schisterman EF, Silver RM, Perkins NJ, *et al.* A randomised trial to evaluate the effects of low-dose aspirin in gestation and reproduction: design and baseline characteristics. *Paediatr Perinat Epidemiol*. 27(6), 598–609 (2013).

31. Yeung EH, Guan W, Zeng X, *et al.* Cord blood DNA methylation reflects cord blood C-reactive protein levels but not maternal levels: a longitudinal study and meta-analysis. *Clin Epigenetics*. 12(1), 60 (2020).

32. Yeung EH, Guan W, Mumford SL, *et al.* Measured maternal prepregnancy anthropometry and newborn DNA methylation. *Epigenomics*. 11(2), 187–198 (2019).

33. Newschaffer CJ, Croen LA, Fallin MD, *et al.* Infant siblings and the investigation of autism risk factors. *J Neurodev Disord*. 4(1), 7 (2012).

34. Leek JT, Storey JD. Capturing heterogeneity in gene expression studies by surrogate variable analysis. *PLoS Genet*. 3(9), 1724–1735 (2007).

35. Johnson WE, Li C, Rabinovic A. Adjusting batch effects in microarray expression data using empirical Bayes methods. *Biostatistics*. 8(1), 118–127 (2007).

36. Heude B, Forhan A, Slama R, *et al.* Cohort Profile: The EDEN mother-child cohort on the prenatal and early postnatal determinants of child health and development. *Int J Epidemiol*. 45(2), 353–363 (2016).

37. R Core Team. R: A language and environment for statistical computing. .

38. Bibikova M, Barnes B, Tsan C, *et al.* High density DNA methylation array with single CpG site resolution. *Genomics*. 98(4), 288–295 (2011).

39. Gagnon-Bartsch JA, Speed TP. Using control genes to correct for unwanted variation in microarray data. *Biostatistics*. 13(3), 539–552 (2012).

40. Xu C-J, Bonder MJ, Söderhäll C, *et al.* The emerging landscape of dynamic DNA methylation in early childhood. *BMC Genomics*. 18(1), 25 (2017).

41. Farchi S, Forastiere F, Vecchi Brumatti L, *et al.* Piccolipiù, a multicenter birth cohort in Italy: protocol of the study. *BMC Pediatr*. 14, 36 (2014).

42. Chatzi L, Leventakou V, Vafeiadi M, *et al.* Cohort Profile: The Mother-Child Cohort in Crete, Greece (Rhea Study). *Int J Epidemiol*. 46(5), 1392–1393k (2017).

43. Janssen BG, Madhloum N, Gyselaers W, *et al.* Cohort Profile: The ENVIRonmental influence ON early AGEing (ENVIRONAGE): a birth cohort study. *Int J Epidemiol*. 46(5), 1386–1387m (2017).

44. Gervin K, Salas LA, Bakulski KM, *et al.* Systematic evaluation and validation of reference and library selection methods for deconvolution of cord blood DNA methylation data. *Clin Epigenetics*. 11(1), 125 (2019).

45. Schoeters G, Den Hond E, Colles A, *et al.* Concept of the Flemish human biomonitoring programme. *Int J Hyg Environ Health*. 215(2), 102–108 (2012).

46. Koppen G, Den Hond E, Nelen V, *et al.* Organochlorine and heavy metals in newborns: results from the Flemish Environment and Health Survey (FLEHS 2002-2006). *Environ Int*. 35(7), 1015–1022 (2009).

47. Maksimovic J, Gordon L, Oshlack A. SWAN: Subset-quantile within array normalization for illumina infinium HumanMethylation450 BeadChips. *Genome Biol*. 13(6), R44 (2012).

48. Guillemette L, Allard C, Lacroix M, *et al.* Genetics of Glucose regulation in Gestation and Growth (Gen3G): a prospective prebirth cohort of mother-child pairs in Sherbrooke, Canada. *BMJ Open*. 6(2), e010031 (2016).

49. Kooijman MN, Kruithof CJ, van Duijn CM, *et al.* The Generation R Study: design and cohort update 2017. *Eur J Epidemiol*. 31(12), 1243–1264 (2016).

50. Lehne B, Drong AW, Loh M, *et al.* A coherent approach for analysis of the Illumina HumanMethylation450 BeadChip improves data quality and performance in epigenome-wide association studies. *Genome Biol*. 16, 37 (2015).

51. Paternoster L, Evans DM, Nohr EA, *et al.* Genome-wide population-based association study of extremely overweight young adults--the GOYA study. *PLoS One*. 6(9), e24303 (2011).

52. Verkleij-Hagoort AC, Verlinde M, Ursem NTC, *et al.* Maternal hyperhomocysteinaemia is a risk factor for congenital heart disease. *BJOG*. 113(12), 1412–1418 (2006).

53. van Iterson M, Tobi EW, Slieker RC, *et al.* MethylAid: visual and interactive quality control of large Illumina 450k datasets. *Bioinformatics*. 30(23), 3435–3437 (2014).

54. van Iterson M, van Zwet EW, BIOS Consortium, Heijmans BT. Controlling bias and inflation in epigenome- and transcriptome-wide association studies using the empirical null distribution. *Genome Biol*. 18(1), 19 (2017).

55. Shapiro ALB, Schmiege SJ, Brinton JT, *et al.* Testing the fuel-mediated hypothesis: maternal insulin resistance and glucose mediate the association between maternal and neonatal adiposity, the Healthy Start study. *Diabetologia*. 58(5), 937–941 (2015).

56. Starling AP, Brinton JT, Glueck DH, *et al.* Associations of maternal BMI and gestational weight gain with neonatal adiposity in the Healthy Start study. *Am J Clin Nutr*. 101(2), 302–309 (2015).

57. Maitre L, de Bont J, Casas M, *et al.* Human Early Life Exposome (HELIX) study: a European population-based exposome cohort. *BMJ Open*. 8(9), e021311 (2018).

58. Guxens M, Ballester F, Espada M, *et al.* Cohort Profile: the INMA--INfancia y Medio Ambiente--(Environment and Childhood) Project. *Int J Epidemiol*. 41(4), 930–940 (2012).

59. Westerway SC, Davison A, Cowell S. Ultrasonic fetal measurements: new Australian standards for the new millennium. *Aust N Z J Obstet Gynaecol*. 40(3), 297–302 (2000).

60. Arshad SH, Holloway JW, Karmaus W, *et al.* Cohort Profile: The Isle Of Wight Whole Population Birth Cohort (IOWBC). *Int J Epidemiol*. 47(4), 1043–1044i (2018).

61. Arshad SH, Patil V, Mitchell F, *et al.* Cohort Profile Update: The Isle of Wight Whole Population Birth Cohort (IOWBC). *Int J Epidemiol*. 49(4), 1083–1084 (2020).

62. Hinz D, Simon JC, Maier-Simon C, *et al.* Reduced maternal regulatory T cell numbers and increased T helper type 2 cytokine production are associated with elevated levels of immunoglobulin E in cord blood. *Clin Exp Allergy*. 40(3), 419–426 (2010).

63. Hinz D, Bauer M, Röder S, *et al.* Cord blood Tregs with stable FOXP3 expression are influenced by prenatal environment and associated with atopic dermatitis at the age of one year. *Allergy*. 67(3), 380–389 (2012).

64. Bauer T, Trump S, Ishaque N, *et al.* Environment-induced epigenetic reprogramming in genomic regulatory elements in smoking mothers and their children. *Mol Syst Biol*. 12(3), 861 (2016).

65. Magnus P, Birke C, Vejrup K, *et al.* Cohort Profile Update: The Norwegian Mother and Child Cohort Study (MoBa). *Int J Epidemiol*. 45(2), 382–388 (2016).

66. Joubert BR, Felix JF, Yousefi P, *et al.* DNA Methylation in Newborns and Maternal Smoking in Pregnancy: Genome-wide Consortium Meta-analysis. *Am J Hum Genet*. 98(4), 680–696 (2016).

67. Hoyo C, Murtha AP, Schildkraut JM, *et al.* Folic acid supplementation before and during pregnancy in the Newborn Epigenetics STudy (NEST). *BMC Public Health*. 11(1), 46 (2011).

68. Girchenko P, Lahti M, Tuovinen S, *et al.* Cohort Profile: Prediction and prevention of preeclampsia and intrauterine growth restriction (PREDO) study. *Int J Epidemiol*. 46(5), 1380–1381g (2017).

69. Price ME, Cotton AM, Lam LL, *et al.* Additional annotation enhances potential for biologically-relevant analysis of the Illumina Infinium HumanMethylation450 BeadChip array. *Epigenetics Chromatin*. 6(1), 4 (2013).

70. Almqvist C, Örtqvist AK, Ullemar V, Lundholm C, Lichtenstein P, Magnusson PKE. Cohort Profile: Swedish Twin Study on Prediction and Prevention of Asthma (STOPPA). *Twin Res Hum Genet*. 18(3), 273–280 (2015).

71. Anckarsäter H, Lundström S, Kollberg L, *et al.* The Child and Adolescent Twin Study in Sweden (CATSS). *Twin Res Hum Genet*. 14(6), 495–508 (2011).

72. Assenov Y, Müller F, Lutsik P, Walter J, Lengauer T, Bock C. Comprehensive analysis of DNA methylation data with RnBeads. *Nat Methods*. 11(11), 1138–1140 (2014).

73. Zetterqvist J, Vansteelandt S, Pawitan Y, Sjölander A. Doubly robust methods for handling confounding by cluster. *Biostatistics*. 17(2), 264–276 (2016).

74. Oken E, Baccarelli AA, Gold DR, *et al.* Cohort profile: project viva. *Int J Epidemiol*. 44(1), 37–48 (2015).
