# Supplementary material for: Analysis of DNA methylation at birth and in childhood reveals changes associated with season of birth and latitude
Source: Clin Epigenetics. 2023 Sep 11;15:148. doi: 10.1186/s13148-023-01542-5 (PMC10496224; doi:10.1186/s13148-023-01542-5)
Supplement: Supplementary file 3 — Additional file 3. “PACE analysis plan for Season of Birth and methylation profiles in children (6 June 2018)”. Analysis plan that was circulated amongst the participant cohorts for the Season of Birth study. [file 13148_2023_1542_MOESM3_ESM.docx]

**PACE analysis plan for Season of Birth and methylation profiles in children**

**(6 June 2018)**

*Lead: Faisal I. Rezwan, Latha Kadalayil, John W. Holloway (IoW)*

*Co-leads: Evelien van Meel, Janine Felix, Vincent Jaddoe, Stephanie London, Liesbeth Duijts (Generation R)*

**Background**

Season of birth has previously been associated with allergic disease risk and children born during autumn or winter are more likely to develop asthma [[1](#_ENREF_1)], rhinitis [[2](#_ENREF_2)], hayfever [[3](#_ENREF_3)], and eczema [[4](#_ENREF_4)]. However, the mechanism by which season of birth is related to disease remains unclear. Transcriptional changes exhibit seasonal periodicity. For example, risk biomarkers for cardiovascular, psychiatric, and autoimmune diseases are increased for those born during winter, along with the incidence of their corresponding disease [[5](#_ENREF_5)]. The epigenome is one method by which environmental factors may alter gene expression that persists over time, leading to an influence upon allergic disease outcome [[6-8](#_ENREF_6)].

A previous study from the Isle of Wight Birth Cohort (18 year old samples) linked birth-associated DNA methylation changes which persist in in early adulthood to season of birth, demonstrating a potential mechanism for a sustained effect upon allergic disease risk [[9](#_ENREF_9)]. Ninety-two CpG sites were associated with season of birth at age 18. These CpGs were then mapped to 79 genes, including the allergy related gene *ATPAF1* and pathway analysis identified three significantly enriched networks related to embryonic development, cell cycle, and cell death. Furthermore, eczema, rhinitis, atopy, and asthma at age 18 were also associated with methylation levels; while 2 CpGs were identified as casually linked to allergic disease.

Methylation levels are known to change over the lifetime of the individual in response to environmental stimuli, and it is entirely possible that season of birth associated CpGs arise after exposure and are not present from birth. While this previous work demonstrated a strong association between the season of birth and epigenetic changes, the cause and effect relationship is not well understood. Several CpGs were associated with the season of birth and allergic disease, but only a single CpGs (cg10063512, *ERLIN2*) was identified in newborns as hypermethylated in autumn-born subjects (*P* = 0.046), with the association persisting in 18 year old subjects (*P* = 1.93x10^-6^). This lack of overlap between methylation that is present from birth and that which develops later in life may indicate that seasons associated methylation changes can occur after development in response to such things as pollen exposure, sunlight levels, and/or viral infection, and an analysis of methylation at both time points is warranted.

**Analytical plan**

**Overall objective**

- To investigate the association of season of birth with methylation

**Specific objectives**

- To investigate DNA methylation profiles in newborn blood and blood collected in later childhood in relation to the season of birth.

**Exposure**

- Season of birth based upon date of birth in 4 categories.
- Northern Hemisphere: Winter (December-February), Spring (March-May), Summer (June-August), and Autumn (September-November).
- Southern Hemisphere: Winter (June-August), Spring (September-November), Summer (December-February), Autumn (March-May).

**Outcome 1: (Newborn DNA methylation)**

Illumina Infinium 450k BeadChip methylation (EWAS) data in **newborn blood** as available in your cohort. Studies with EPIC 850k chip data are also invited to participate. Untransformed beta values will be used as the outcome. Please normalize using your preferred normalization package and indicate which that was. Use preferred study QC settings for probe filtering. If in any doubt, please include rather than exclude probes at this stage. We are not excluding outlier values in the betas, but would like a log file to be generated and included in the results. (Pasted at the end of this document) and include a log file that contains the number of CpGs outside of the usual range.

- **Primary exposure variable: Season of birth as defined below.** Please treat this as a categorical variable with Autumn as the reference**.** For encoding purposes, you may choose to use season names (e.g. Autumn, Spring, etc.) or encode them as 1, 2, 3, and 4. If you choose the latter, please make sure they are set as factors with 1=Autumn, 2=Winter, 3=Spring, and 4=Summer
  - Northern Hemisphere: Winter (December-February), Spring (March-May), Summer (June-August), and Autumn (September-November).
  - Southern Hemisphere: Winter (June-August), Spring (September-November), Summer (December-February), Autumn (March-May).

**Covariates**

- **Gender:** Binary encoding
- **Maternal Age:** continuous
- **Gestational Age:** continuous (weeks)
- **Maternal smoking status:** Preferred categorization is: 1. No smoking in pregnancy, 2. Smoking, but stopped in early pregnancy, 3. Smoking throughout pregnancy. If you do not have the ability to create these three levels (or you get into small numbers) from your data please contact us to discuss – if you have small numbers of smokers preferred alternative would be smoked throughout pregnancy versus did not smoke throughout (reference would then include people who quit early on).
- **Maternal social class**: definition as in cohort
- **Batch** covariates: Adjustment for batch effects should be done by including the most important covariates (for example, plate) from each individual cohort. Alternatively a batch correction method such as ComBat is fine. Please indicate clearly in the readme file which covariates you included.
- **Estimated cell types:** Please include in relevant models (see below). These cell proportions should be included additively in the model (i.e. proportion of granulocytes + proportion of NK cells etc.). Cell proportions are estimated using estimateCellCounts () in the *minfi* R package. For newborn methylation, please use the Bakulski et al. cord blood reference panel and include the **7 cell types** created including the nucleated red blood cell output of estimateCellCounts (): nRBC,CD8T, CD4T, NK, Bcell, Mono, Gran.
- ***Ancestry***: optional covariate. Please analyze major ethnic groups separately (e.g. European, Latino, African, Asian ancestry). If necessary, a cohort may include PCs from a GWAS within a specific ancestry if available. Please define clearly the method utilized in each ethnicity.
- ***Selection factors***: optional covariate. Please include if relevant for your study, for example if your sample contains cases and controls of some condition, please include the case/control variable.
- Please do not include further covariates. If you feel strongly that you need to include additional covariates, please contact us.

**Outcome 2: (Childhood DNA methylation)**

Illumina Infinium 450k BeadChip methylation (EWAS) data in **Childhood blood**. Studies with EPIC 850k chip data are also invited to participate. Untransformed beta values will be used as the outcome. Please normalize using your preferred normalization package and indicate which that was. Use preferred study QC settings for probe filtering. If in any doubt, please include rather than exclude probes at this stage. We are not excluding outlier values in the betas, but would like a log file to be generated and included in the results. (Pasted at the end of this document) and include a log file that contains the number of CpGs outside of the usual range.

- **Primary exposure variable: Season of birth as defined below.** Please treat this as a categorical variable with Autumn as the reference**.** For encoding purposes, you may choose to use season names (e.g. Autumn, Spring, etc.) or encode them as 1, 2, 3, and 4. If you choose the latter, please make sure they are set as factors with 1=Autumn, 2=Winter, 3=Spring, and 4=Summer.
  - Northern Hemisphere: Winter (December-February), Spring (March-May), Summer (June-August), and Autumn (September-November).
  - Southern Hemisphere: Winter (June-August), Spring (September-November), Summer (December-February), Autumn (March-May).

**Covariates**

- **Gender:** Binary encoding
- **Age (at collection):** continuous
  - **Childhood samples are defined as samples taken between 1 and 10 years of age.**
  - **If you have data from the same patient measured at different time points within this age range, please use the youngest time point in this analysis.**
- **Maternal Age:** continuous
- **Gestational Age:** continuous (weeks).
- **Maternal smoking status:** Preferred categorization is: 1. No smoking in pregnancy, 2. Smoking, but stopped in early pregnancy, 3. Smoking throughout pregnancy. If you do not have the ability to create these three levels (or you get into small numbers) from your data please contact us to discuss – if you have small numbers of smokers preferred alternative would be smoked throughout pregnancy versus did not smoke throughout (reference would then include people who quit early on).
- **Season of sample collection (Please use the same encoding as for the exposure variable, i.e., categorical variable with Autumn set as the reference).**
  - Northern Hemisphere: Winter (December-February), Spring (March-May), Summer (June-August), and Autumn (September-November).
  - Southern Hemisphere: Winter (June-August), Spring (September-November), Summer (December-February), Autumn (March-May).
- **Maternal social class**: definition as in cohort
- **Batch** covariates: Adjustment for batch effects should be done by including the most important covariates (for example, plate) from each individual cohort. Alternatively a batch correction method such as ComBat is fine. Please indicate clearly in the readme file which covariates you included.
- **Estimated cell type:** Please include in relevant models (see below). These cell proportions should be included additively in the model (i.e. proportion of granulocytes + proportion of NK cells etc.). Cell proportions are estimated using estimateCellCounts () in the *minfi* R package. For childhood blood, please use the Houseman method (Reinius adult reference population) and include the **6 cell types** created in the default output of estimateCellCounts (): CD8T, CD4T, NK, Bcell, Mono, Gran for whole blood (Houseman method).
- ***Ancestry***: optional covariate. Please analyze major ethnic groups separately (e.g. European, Latino, African, Asian ancestry). If necessary, a cohort may include PCs from a GWAS within a specific ancestry if available. Please define clearly the method utilized in each ethnicity.

Please do not include further covariates. If you feel strongly that, you need to include additional covariates, please contact us. Should you have any questions regarding any of these covariates, please contact us for clarification.

Provide descriptive table of outcome variables and covariates; see Table 2:

- Frequencies for categorical variables.
- For continuous variables provide mean and SD.

While not included as a co-factor in the initial analysis, please also provide an indication of the geographical area from which subjects are recruited. Post hoc analysis may include test for effects of latitude on effect size.

**Models and analyses**

- Robust linear regression modelling (rlm() option in R) for each CpG site individually.

1. *Cord Blood Models*
   1. *Crude model for cord blood*
      1. Methylation = Season of birth + (Batch)
   2. *Covariate model for cord blood: Season of birth with covariates:*
      1. Methylation = Season of Birth + Gender + Maternal Age + Gestational Age + Maternal Smoking status + Maternal social class + Ancestry (if applicable) + (Batch)
   3. *Cell type model for cord blood: Season of birth with all covariates and cell type proportions:*
      1. Methylation = Season of Birth + Gender + Maternal Age + Gestational Age + Maternal Smoking status + Maternal social class + Ancestry (if applicable) + (Batch) + Cell type proportions
2. *Non-Cord Blood Models (differences highlighted in* ***Bold****)*
   1. *Crude model for non-cord blood*
      1. Methylation = Season of birth + (Batch)
   2. *Covariate model for non-cord blood: Season of birth with covariates:*
      1. Methylation = Season of Birth + Gender + Maternal Age + Gestational Age + Maternal Smoking status + Maternal social class + **Age + Season of sample collection** + Ancestry (if applicable) + (Batch)
   3. *Cell type model for non-cord blood: Season of birth with all covariates and cell type proportions:*
      1. Methylation = Season of Birth + Gender + Maternal Age + Gestational Age + Maternal Smoking status + Maternal social class + **Age** + **Season of sample collection** + Ancestry (if applicable) + (Batch) + Cell type proportions

- Please do not adjust your P-values for multiple testing.

**Exclusions**

- Exclude multiple births (i.e. singleton only analysis) and multiple siblings from the same family (include 1 child per family in case of multiple siblings from the same family).

###### **How to deal with missing variables**

- Exclude samples with missing observations for any of the covariates (be sure **not** to include them in the reference group).
- Alternative to deleting subjects with missing covariates is to use a published modern method for multiple imputation (such as MICE – multiple imputation with chained equations). If you used multiple imputation, please provide the method and the software package used to implement it. Do not use homegrown, unpublished, or outdated methods for dealing with missing values (such as modeling the missing as a category) – exclude missing instead. If you have questions please ask us before running models.

**Time plan**

- April 15, 2018 Project plan finalized
- September 1, 2018 Results uploaded
- January 1, 2019 Meta-analyses completed
- May 2019 Paper submission

**Data file format**

- Tab delimited file; one row per probe. First row is a header with labels as defined in the table below.
- For file naming, please use the following convention:
- SB_TYPE_SUBTYPE_ STUDY_DATE.txt.gz
- TYPE is “Cordblood” or “Non-cordblood”
  - If you have only a single non-cordblood age, please include that as Non-cordblood-Age##”. If you have multiple ages, just leave this as Non-cordblood and explain in the readme file.
- SUBTYPE is “Crude” (model a) or “Covariate” (model b) or “CellType” (model c)
- STUDY is a short cohort name (e.g. Isle of Wight is IOW)
- DATE is the date on which the file was prepared. Please use the following format “YYYYMMDD”.
- For example: SB_Cordblood_Crude_IOW_20161006.txt.gz
- The table containing probe/CpG summary statistics should include **for each CpG** the minimum, 10% quantile, 25% quantile, median, mean, sd, 75% quantile, 90% quantile, maximum, and number of missing samples (see Example Code for R code).
- Please use naming convention SB_Cohort_Descriptives.txt (e.g. SB_IOW_Descriptives.txt) when uploading.
- A table containing total sample size, calculated lambda for each model (see Example Code and Table 1). **Please** **provide lambda values and failed CpG counts for each model**.
  - Please use naming convention **SB_Cohort_TYPE_SUBTYPE_SampleSizeLambda.txt** (e.g. SB_IOW_Non-cordblood_Covariate_SampleSizeLambda.txt).
- Please include a table with the descriptive statistics for each exposure and covariate and the number at each level of the covariate (see Table 2 at the end of this document). Call this SB_**Cohort_covariates_table.doc** or **.xls** (e.g. SB_IOW_covariates_table.doc).
- Please include the log file of potential outliers and use the naming convention **SB_Cohort_OutlierLog.txt** (e.g. SB_IOW_OutlierLog.txt).
- Please put all .txt files into one folder, compress this folder, and use naming convention **SB_Cohort_results.tar.gz**.
  - Compress using **tar -zcvf SB_Cohort_results.tar.gz SB_Cohort_results/** in Unix (e.g. tar -zcvf SB_IOW_results.tar.gz SB_IOW_results/)
- Data file contents requirements:
- If you have missing data in your results file, please do not leave any cells blank. Missing data should be denoted by NA.
- No quotes should be used around any data cells or headers.
- Please provide all numeric data with at least 4 decimal places. P-values should be specified to at least 4 relevant digits.
- **README file**: With your data, please upload a readme file with a short description of:
- the normalization and QC steps taken in your study
- If relevant, information about the ages included
- Any additional information about the uploaded files

**Results format table**

| Column Header | Description | Format | Examples |
| --- | --- | --- | --- |
| probeID | Probename | String | cg23094576  ch.10.135292R |
| BETA | Effect size | Numeric | 0.2036 |
| SE | Standard error of the BETA | Numeric | 0.5611 |
| P_VAL | P value for the probe | Scientific E notation with at least 3 digits to the right of the decimal | 3.244E-10 |

**Data exchange**

When finished, contact us, and we will send a link for result upload.

**Primary contacts for questions about the analysis:**

Latha Kadalayil ([lpk1r12@soton.ac.uk](mailto:lpk1r12@soton.ac.uk))

Faisal I. Rezwan ([F.Rezwan@soton.ac.uk)](mailto:F.Rezwan@soton.ac.uk))

John W. Holloway ([J.W.Holloway@soton.ac.uk](mailto:J.W.Holloway@soton.ac.uk))

**References**

1. Knudsen, T.B., et al., *Season of birth and risk of atopic disease among children and adolescents.* J Asthma, 2007. **44**(4): p. 257-60.

2. Arshad, S.H., M. Stevens, and D.W. Hide, *The effect of genetic and environmental factors on the prevalence of allergic disorders at the age of two years.* Clin Exp Allergy, 1993. **23**(6): p. 504-11.

3. Pearson, D.J., D.L. Freed, and G. Taylor, *Respiratory allergy and month of birth.* Clin Allergy, 1977. **7**(1): p. 29-33.

4. Tariq, S.M., et al., *The prevalence of and risk factors for atopy in early childhood: a whole population birth cohort study.* J Allergy Clin Immunol, 1998. **101**(5): p. 587-93.

5. Dopico, X.C., et al., *Widespread seasonal gene expression reveals annual differences in human immunity and physiology.* Nat Commun, 2015. **6**: p. 7000.

6. Guthikonda, K., et al., *Oral contraceptives modify the effect of GATA3 polymorphisms on the risk of asthma at the age of 18 years via DNA methylation.* Clin Epigenetics, 2014. **6**(1): p. 17.

7. Soto-Ramirez, N., et al., *The interaction of genetic variants and DNA methylation of the interleukin-4 receptor gene increase the risk of asthma at age 18 years.* Clin Epigenetics, 2013. **5**(1): p. 1.

8. Ziyab, A.H., et al., *DNA methylation of the filaggrin gene adds to the risk of eczema associated with loss-of-function variants.* J Eur Acad Dermatol Venereol, 2013. **27**(3): p. e420-3.

9. Lockett, G.A., et al., *Association of season of birth with DNA methylation and allergic disease.* Allergy, 2016. **71**(9): p. 1314-24.

**Example R code: Please email Faisal Rezwan (**[**F.Rezwan@soton.ac.uk**](mailto:F.Rezwan@soton.ac.uk)**) for questions, clarifications, or R code.**

*# libraries*

library(data.table)# to process results

library(MASS) # rlm function for robust linear regression

library(sandwich) # Huberís estimation of the standard error

library(lmtest) # to use coeftest

library(parallel) # to use multicore approach - part of base R

*# Set working and phenotype directory and store it*

workdir<-paste("myworkdir")

phenodir<-paste("myphenodir")

setwd(workdir)

*# set names for later use.*

*# Future code, particularly writing tables for output, assumes you have set these.*

NAME<-"SB"

TYPE<-"Non-cordblood-Age10YR"

SUBTYPE<-"Covariate"

STUDY<-"IOW"

*# Adjust if your starting date format is different from yyyy-mm-dd*

DATE<-gsub("-","",Sys.Date())

phenofile<-"SB_pheno2.csv"

*# Set number of cores. If running on Windows, can only be set to 1.*

cores=8

*# load methylation data as beta values, probe annotation data, phenotype data, batch data.*

*# Code below assumes beta values start out with rows as CpG sites and columns as samples. If it is different, transpose it before applying code.*

*# make sure that phenotype data is being treated in the correct way by R (e.g. factors for categorical data, with the first level of the factor being the reference or baseline category)*

*# See examples below.*

*# Read pheno file, subset if needed, and order*

phe<-read.csv(paste(phenodir,"/",phenofile,sep=""),na.strings=c("","NA"))

*# Remove samples with missing data.*

phe<-na.omit(phe)

*# Subset phe by beta matrix columns. Assumes sample ID column is named "Sample_ID", change accordingly.*

phe<-phe[phe$Sample_ID%in%colnames(beta_matrix),]

*# Order beta matrix according to pheno file*

beta_matrix<-beta_matrix[,match(phe$Sample_ID,colnames(beta_matrix))]

*# Check ordering. If FALSE, samples are not matched properly.*

all(colnames(beta_matrix)==phe$Sample_ID)

*# transpose betas so that rows are samples and columns are probes*

beta_matrix<-t(beta_matrix)

*# set variables for design formula*

*# NOTE: season names for birth and collection must be named differently or you will get an error in the model.*

*# If you include additional covariates, add them below and in the design object further below.*

Season_Birth<-factor(phe$Season_Birth,

levels=c("Autumn","Winter","Spring","Summer"))

*# Change as needed, but keep levels for season the same.*

Sex<-factor(phe$SEX_03)

Mat_age<-phe$Mat_age

Gest_age<-phe$GESTAGE

Msmk<-factor(phe$MSMK_03)

SocioEc<-phe$SOCIOEC

Age_collect<-phe$Age_collect

Season_collect<-factor(phe$Season_collect,

levels=c("Autumn_collect","Winter_collect","Spring_collect","Summer_collect"))

*# create design for use in rlm function*

*# adjust model for additional covariates. Season_Birth must be the first variable.*

design<-as.formula("beta_matrix[,methcol] ~ Season_Birth + Sex + Mat_age +

Gest_age + Msmk + SocioEc + Age_collect + Season_collect")

*# Add function for running the model. This function uses the object “design”. If you change the model, adjust that object above and make sure the additional covariates exist in your workspace. This function will take the first 3 covariates which should be birth during winter, spring, and summer with autumn as the reference. Occasionally, you may run into an error with the coeftest function. This function is designed to identify this error and produce NAs as output when this occurs instead of halting the function.*

RLMtest=function(meth_matrix,methcol,design) {

methcol<<-methcol

mod = try(rlm(design,maxit=200))

if(class(mod) == "try-error"){

print(paste("error thrown by column", methcol))

invisible(rep(NA, 3))

}else cf = try(coeftest(mod, vcov=vcovHC(mod, type="HC0")))

if(class(cf)=="try-error"){

print(paste("error in coeftest by column", methcol,"setting values to NA"))

output<-rep(NA,9)

}else{keep<-c("Estimate","Std. Error","Pr(>|z|)")

*# Pull out winter, spring, and summer.*

output<-c(cf[2,keep],cf[3,keep],cf[4,keep])

names1<-rownames(cf)[2:4]

names2<-c("BETA","SE","P_VAL")

names_full=matrix(NA,nrow=length(names2),ncol=length(names1))

for (i in 1:length(names1)){

for (j in 1:length(names2)){

names_full[j,i]<-paste(names1[i],names2[j],sep="_")

}

}

names_full<-as.vector(names_full)

names(output)<-names_full

output

}

}

*# Run adjusted EWAS*

system.time(ind.res <- mclapply(setNames(seq_len(ncol(beta_matrix)),

dimnames(beta_matrix)[[2]]), RLMtest, mc.cores=cores,meth_matrix=beta_matrix,

design=design))

*# Process results*

Names<-names(ind.res[[1]])

setattr(ind.res, 'class', 'data.frame')

setattr(ind.res, "row.names", c(NA_integer_,9))

setattr(ind.res, "names", make.names(names(ind.res), unique=TRUE))

probelistnames <- names(ind.res)

all.results <- t(data.table(ind.res))

all.results<-data.table(all.results)

all.results[, Probe_ID := probelistnames]

setnames(all.results, c(Names,"Probe_ID")) # rename columns

setcolorder(all.results, c("Probe_ID",Names))

rm(probelistnames, ind.res)

*# export table of results*

(paste(paste(NAME,TYPE,SUBTYPE,STUDY,DATE,sep="_"),".txt",sep=""))

write.table(all.results, paste(paste(NAME,TYPE,SUBTYPE,STUDY,DATE,sep="_"),".txt",sep="")

,na="NA",row.names=FALSE,sep="\t",quote=FALSE)

gzip(paste(paste(NAME,TYPE,SUBTYPE,STUDY,DATE,sep="_"),".txt",sep=""))

##### Calculate lambdas

lambda_fun<-function(x){

qchisq(median(x,na.rm=T), df =1, lower.tail=F)/qchisq(0.5,1)

}

*# Extract P_VAL columns*

P_VALS<-all.results[,grepl("_P_VAL",colnames(all.results)),with=FALSE]

lambdas<-apply(P_VALS,2,lambda_fun)

N<-dim(beta_matrix)[1]

CpGFails<-sum(is.na(all.results[,2]))

Tablestats<-t(c(N,CpGFails,lambdas))

colnames(Tablestats)[c(1,2)]<-c("Sample_Count","CpGFails")

colnames(Tablestats)<-gsub("_P_VAL","_Lambda",colnames(Tablestats))

*# Write file*

write.table(Tablestats,file=paste(NAME,TYPE,SUBTYPE,STUDY,"SampleSizeLambda.txt",

sep="_"),sep="\t",row.names=FALSE,quote=FALSE)

# Code created by PACE collaboratory.

########################## SUMMARIZE PROBES ##########################

descriptives<-function(x){

tmp<-c(min(x,na.rm=T),quantile(x,probs=c(.1,.25,.5),na.rm=T),mean(x,na.rm=T), median(x,na.rm=T),sd(x,na.rm=T),quantile(x,probs=c(.75,.90),na.rm=T),max(x,na.rm=T),sum(is.na(x)))

names(tmp)[c(1,5:7,10:11)]<-c("Min.","Mean","Median","SD","Max.","NA")

return(tmp)

}

desc<-t(apply(beta_matrix,2,descriptives))

desc<-cbind(rownames(desc),desc)

colnames(desc)[1]<-"Probe_ID"

write.table(desc, file = paste(workdir,"/",NAME,"_",STUDY,"_Descriptives.txt",

sep = ""), sep = "\t", col.names = T, row.names = F, append = F, quote=FALSE)

#################### Code for trimming ###############################

Code for trimming methylation beta values to remove potential outliers

Date created: June 12, 2015

Trimming scheme is as follows – trim values beyond the lower and upper outer fences. These are defined by:

Values < 25^th^ percentile minus 3*IQR AND Values > 75^th^ percentile plus 3*IQR

(IQR = interquartile range)

The trimming should be done on the file that you use for PACE analyses. For most cohorts this is normalized beta values.

The following code was provided by Gemma Sharp on June 12, 2105 – modification (to run faster) of code provided earlier by Janine Felix. Input also from Allan Just.

#Function

removeOutliers<-function(probes){

require(matrixStats)

if(nrow(probes) < ncol(probes)) warning("expecting probes are rows (long dataset)")

rowIQR <- rowIQRs(probes, na.rm = T)

row2575 <- rowQuantiles(probes, probs = c(0.25, 0.75), na.rm = T)

maskL <- probes < row2575[,1] - 3 * rowIQR

maskU <- probes > row2575[,2] + 3 * rowIQR

initial_NAs<-rowSums([is.na](http://is.na/)(probes))

probes[maskL] <- NA

removed_lower <- rowSums([is.na](http://is.na/)(probes))-initial_NAs

probes[maskU] <- NA

removed_upper <- rowSums([is.na](http://is.na/)(probes))-removed_lower-initial_NAs

N_for_probe<-rowSums(![is.na](http://is.na/)(probes))

Log<-data.frame(initial_NAs,removed_lower,removed_upper,N_for_probe)

return(list(probes, Log))

}

#Remove outliers from METH (methylation data where probes are rows and samples are columns)

system.time(OutlierResults<-removeOutliers(beta_matrix))  #user  system elapsed 375.885   9.879  386.837 on 485577 probes, 914 samples

beta_matrix.2<-OutlierResults[[1]]

Log<-OutlierResults[[2]]

Log<-cbind(rownames(Log),Log)

colnames(Log)[1]<-"CpGs"

write.table(Log,file= paste(workdir,"/",NAME,"_",STUDY,"_Outlier_Log.txt",

sep = ""), sep = "\t", col.names = T, row.names = F, append = F, quote=FALSE)

**Cord blood samples:**

NAME OF STUDY:_________________________________________________________________

If you used any categorizations other than those listed, please put an X in the relevant box(es) and add your own categorizations to the bottom of the table.

Table 1

| Model | N | Lambda (Winter) | Lambda (Spring) | Lambda (Summer) | CpG Fails |
| --- | --- | --- | --- | --- | --- |
| Model 1a |  |  |  |  |  |
| Model 1b |  |  |  |  |  |
| Model 1c |  |  |  |  |  |

**Table 2**

|  |  |
| --- | --- |
| N in analysis (complete cases after adjustment for covariates) |  |
| Season of Birth  N summer |  |
| N autumn |  |
| N winter |  |
| N spring |  |
| Gender  N male |  |
| N female |  |
| Maternal Age (Mean +/- SD) |  |
| Gestational Age (Mean +/- SD) |  |
| N Maternal Smoking Status (Cohort definition) |  |
| Maternal social class  N Your measure of social class (please give details) group 1 |  |
| N Your measure of social class (please give details) group 2 |  |
| N Your measure of social class (please give details) group 3 |  |
| N Your measure of social class (please give details) group 4, etc. |  |
| Latitude of Cohort |  |

**Non-cord blood samples:**

NAME OF STUDY:________________________________________________________________

If you used any categorizations other than those listed, please put an X in the relevant box(es) and add your own categorizations to the bottom of the table.

Table 2

| Model | N | Lambda (Winter) | Lambda (Spring) | Lambda (Summer) | CpG Fails |
| --- | --- | --- | --- | --- | --- |
| Model 2a |  |  |  |  |  |
| Model 2b |  |  |  |  |  |
| Model 2c |  |  |  |  |  |

**Table 2**

|  |  |
| --- | --- |
| N in analysis (complete cases after adjustment for covariates) |  |
| Mean age +/- SD |  |
| Gestational Age +/- SD |  |
| Maternal Age +/- SD |  |
| Season of Birth  N summer |  |
| N autumn |  |
| N winter |  |
| N spring |  |
| Season of sample collection  N summer |  |
| N autumn |  |
| N winter |  |
| N spring |  |
| Gender  N male |  |
| N female |  |
| N Maternal Smoking Status (Cohort definition) |  |
| Maternal social class  N Your measure of social class (please give details) group 1 |  |
| N Your measure of social class (please give details) group 2 |  |
| N Your measure of social class (please give details) group 3 |  |
| N Your measure of social class (please give details) group 4, etc. |  |
| Latitude of Cohort |  |
