# Supplementary material for: Analysis of DNA methylation at birth and in childhood reveals changes associated with season of birth and latitude
Source: Clin Epigenetics. 2023 Sep 11;15:148. doi: 10.1186/s13148-023-01542-5 (PMC10496224; doi:10.1186/s13148-023-01542-5)
Supplement: Supplementary file 4 — Additional file 4. Table S2: “Models used in this meta-analysis study”. Inflation (lambda) and bias information of the models. Table S3: “Comparison of magnitude and direction of the FDR-significant DNA methylation signals identified in the at-birth and childhood meta-analyses”. [file 13148_2023_1542_MOESM4_ESM.docx]

**Table S2**: Models used in this meta-analysis study

|  | **At-birth** | | |  | **Childhood** | | |
| --- | --- | --- | --- | --- | --- | --- | --- |
| **Season of birth** | **Probes (N)** | **Lambda*** | **Bias*** |  | **Probes (N)** | **Lambda** | **Bias** |
| ***Main models*** |  |  |  |  |  |  |  |
| Winter | 470870 | 1.02 | 0.135 |  | 470857 | 1.03 | 0.077 |
| Spring | 470870 | 1.05 | 0.085 |  | 470850 | 1.04 | 0.019 |
| Summer | 470870 | 1.04 | 0.253 |  | 470861 | 1.16 | 0.028 |
| ***Latitude models*** |  |  |  |  |  |  |  |
| *Higher latitude (≥50°N)* |  |  |  |  |  |  |  |
| Winter | 470870 | 1.13 | 0.137 |  | 470852 | 1.12 | -0.058 |
| Spring | 470870 | 1.01 | 0.040 |  | 470843 | 1.07 | -0.157 |
| Summer | 470870 | 1.07 | 0.433 |  | 470855 | 1.11 | -0.011 |
| *Lower latitude (<50°N)* |  |  |  |  |  |  |  |
| Winter | 470869 | 1.02 | -0.140 |  | X | X | X |
| Spring | 470869 | 1.06 | 0.037 |  | X | X | X |
| Summer | 470869 | 0.99 | -0.065 |  | X | X | X |

Robust linear regression was used to meta-analyse the covariate- and cell type proportion-adjusted cohort-specific (epigenome-wide association study) EWAS summary results. The outcome was DNA methylation (beta values) in cord or heel prick blood (at-birth) and whole blood (childhood, 1-11 years) and the exposure of interest was season of birth. Children born in autumn were used as the reference group in all cohort specific EWAS analyses. All EWAS models were adjusted for maternal smoking status during pregnancy, socio-economic status, gestational age and maternal age at delivery, new-born’s sex, and estimated cell type proportions. The childhood models were also adjusted for the child’s age and season of sample collection if data were available. Childhood analysis for the lower latitude subgroup (X) was not done due to a smaller sample size (n = 936).

*Estimated using BACON method of van Iterson et al, *Genome Biol*. 18(1), 19 (2017).

**Table S3:** Comparison of magnitude and direction of the FDR-significant DNA methylation signals identified in the at-birth and childhood meta-analyses

| **Season of birth^a^** | **CpG** | **Coeff** | **SE** | **p-value** | **FDR**  **p-value** | **Direction of change in DNA methylation^b^** | | | **I^2^** |
| --- | --- | --- | --- | --- | --- | --- | --- | --- | --- |
|  |  |  |  |  |  |  | **Cohorts with Hyper-methylation (n, %)** | **Cohorts with Hypo-methylation (n, %)** |  |
| ***Significant CpGs at birth*** | | | | | | | | | |
| ***At-birth*** |  |  |  |  |  |  |  |  |  |
| Winter | cg26416241 | 0.0034 | 0.0006 | **6.02 X 10^-8^** | 0.014 | +++-++++-+-++++-++-++++- | 18 (75%) | 6 (25%) | 42.5 |
| Spring | cg18978324 | 0.0006 | 0.0001 | **2.61 X 10^-8^** | 0.012 | -++++++?+-++++++?+-+-++- | 17 (77% | 5 (23%) | 27 |
| ***Childhood*** |  |  |  |  |  |  |  |  |  |
| Winter | cg26416241 | 0.0001 | 0.0012 | 0.9454 | 0.997 | +-++-+++--+- | 7 (58%) | 5 (42%) | 35.5 |
| Spring | cg18978324 | 0 | 0.0002 | 0.7952 | 0.988 | ++++-+-+--+- | 7 (58%) | 5 (42%) | 0 |
| ***Significant CpGs in childhood*** | | | | | | | | | |
| ***Childhood*** |  |  |  |  |  |  |  |  |  |
| Summer | cg19416462 | -0.0037 | 0.0007 | **1.99 X 10^-8^** | 0.009 | - - - - - - - + - - - - | 1 (8%) | 11 (92%) | 45.9 |
|  | cg01656588 | -0.007 | 0.0013 | 1.47 X 10^-7^ | 0.026 | - - - - - - - - - - - - | 0 | 12 (100%) | 0 |
|  | cg03263237^c^ | -0.0157 | 0.003 | 1.67 X 10^-7^ | 0.026 | - -?- - -?- -?+? | 1 (11%) | 7 (78%) | 24.6 |
|  | cg15437053 | 0.0037 | 0.0007 | 2.69 X 10^-7^ | 0.032 | +?++++++++++ | 10 (43%) | 13 (57%) | 32.9 |
| ***At-birth*** |  |  |  |  |  |  |  |  |  |
| Summer | cg19416462 | 0.0002 | 0.0004 | 0.674 | 0.958 | ++?-+-+-----+--+-+-+--++ | 10 (43%) | 13 (57%) | 13.4 |
|  | cg01656588 | 0.001 | 0.001 | 0.307 | 0.866 | -+?-+-+----++-++---+--++ | 10 (43%) | 13 (57%) | 0 |
|  | cg03263237^c^ | -0.0006 | 0.0009 | 0.4723 | 0.4723 | +---++--???++--+-++--?++ | 10 (50%) | 10 (50%) | 0.231 |
|  | cg15437053 | -0.0003 | -0.0003 | 0.3086 | 0.866 | ++?-+-+----+---+---+-++- | 9 (39%) | 14 (61%) | 0 |

Meta-analyses of EWAS summary results carried out with data from 21 and 12 cohorts for at-birth and childhood blood samples respectively. CpGs shown in the second column are CpGs that passed the FDR (False discovery rate) p-value threshold of 5% in the meta-analysis of at-birth or childhood meta-analysis. The DNA methylation change in the FDR-significant CpGs from the at-birth analyses in winter and spring (against autumn) were compared to the DNA methylation changes of the same CpGs in the respective birth seasons of the childhood. This was repeated for the FDR-significant CpGs of the childhood analyses as well. We specifically looked for the statistical evidence and direction of methylation changes in the contributing cohorts. The p-values for the CpG sites that also passed the Bonferroni-corrected significance threshold of 1.06 X 10^-7^ are shown in bold. All cohort specific EWAS analyses were adjusted for sex of the child, gestational age at delivery, maternal age at delivery, maternal smoking during pregnancy, maternal socio-economic status, batch, child’s age at the time of sample collection (in the childhood analyses) and estimated cell proportions.

Coeff: regression coefficient (change in mean methylation compared to autumn reference); SE: standard error of the coefficient; Lambda: genomic inflation factor; CHR: chromosome; I^2^: a measure of heterogeneity between studies

**^a^**Reference season for the EWAS analyses of individual cohorts: autumn

**^b^**Hyper-methylation is indicated by “+” and hypo-methylation by “-“ (minus sign)

^c^ Cross-reactive probe (Chen *et al*, Ref: [46] in the main text)
