# Supplementary material for: Analysis of DNA methylation at birth and in childhood reveals changes associated with season of birth and latitude
Source: Clin Epigenetics. 2023 Sep 11;15:148. doi: 10.1186/s13148-023-01542-5 (PMC10496224; doi:10.1186/s13148-023-01542-5)
Supplement: Supplementary file 7 — Additional file 7. Table S6: “Season of birth-associated differentially methylated regions (DMRs) at birth in babies born in latitudes < 50°N”. Differentially methylated regions and mapped genes identified in the at-birth samples of babies born in latitudes < 50°N (lower latitude subgroup analysis). Table S7: “Season of birth associated with differentially methylated regions (DMRs) in children born in latitudes ≥ 50°N”. [file 13148_2023_1542_MOESM7_ESM.docx]

**Table S6**: Season of birth-associated differentially methylated regions (DMRs) at birth in babies born in latitudes <50°N

| **Season of birth^a^** | **CHR** | **Region** | **CpGs in**  **the region** | **Šidák**  **p-value^b^** | **Minimum p-value^c^** | **Gene (hg19)** | **Gene group** |
| --- | --- | --- | --- | --- | --- | --- | --- |
| Winter |  |  |  |  |  |  |  |
|  | 1 | 230468439 - 230468798 | 5 | 1.5 x 10^-5^ | 3.0 x 10^-5^ | *PGBD5* | Intron, cds |
|  | 10 | 53459077 - 53459783 | 13 | 2.4 x 10^-3^ | 7.2 x 10^-4^ | *PRKG1;CSTF2T* | intron; TSS, cds |
|  | 4 | 206087 - 206587 | 8 | 3.5 x 10^-3^ | 2.5 x 10^-3^ | *ZNF876P* | nc_intron, nc_exon |
|  | 6 | 31275526 - 31275906 | 11 | 7.7 x 10^-3^ | 2.8 x 10^-3^ | *LINC02571* | intergenic |
|  | 12 | 75784830 - 75785122 | 6 | 7.9 x 10^-3^ | 2.5 x 10^-3^ | *GLIPR1L2* | nc_exon; TSS, 5’UTR, cds |
| Spring |  |  |  |  |  |  |  |
|  | 4 | 186732812 - 186733085 | 7 | 2.6 x 10^-12^ | 3.1 x 10^-12^ | *SORBS2* | Intron; exon, 5’UTR; intron |
|  | 17 | 47091314 - 47092297 | 7 | 1.2 x 10^-6^ | 1.7 x 10^-3^ | *IGF2BP1* | intron |
|  | 6 | 106035465 - 106035553 | 2 | 4.5 x 10^-6^ | 9.9 x 10^-7^ | *PREP* | intergenic |
|  | 19 | 11784489 - 11785150 | 10 | 2.6 x 10^-5^ | 1.0 x 10^-5^ | *ZNF833P* | nc_intron, nc_exon |
|  | 8 | 143859644 - 143860015 | 7 | 2.9 x 10^-5^ | 1.7 x 10^-5^ | *LYNX1-SLURP2, LYNX1* | intergenic; intergenic |
|  | 11 | 1319623 - 1319738 | 3 | 9.7 x 10^-4^ | 8.7 x 10^-5^ | *TOLLIP* | intron; intron’ 5’UTR |
|  | 17 | 17603506 - 17603862 | 4 | 9.9 x 10^-4^ | 2.7 x 10^-5^ | *RAI1* | Intron, 5’UTR |
|  | 6 | 41068528 - 41068777 | 7 | 2.3 x 10^-3^ | 3.9 x 10^-4^ | *NFYA, ADCY10P1* | Exon, 3’UTR; nc_exon |
|  | 17 | 27045018 - 27045327 | 6 | 2.4 x 10^-3^ | 4.3 x 10^-4^ | *RAB34* | TSS, intron,5’UTR,cds; exon |
|  | 1 | 27961309 - 27961893 | 6 | 3.8 x 10^-3^ | 9.7 x 10^-4^ | *FGR* | TSS, intron, exon, 5’UTR |
|  | 11 | 60534886 - 60535091 | 4 | 5.8 x 10^-3^ | 6.1 x 10^-4^ | *MS4A15* | nc_intron, nc_exon; intron; cds; 5’UTR, cds |
| Summer |  |  |  |  |  |  |  |
|  | 7 | 150019930 - 150020426 | 10 | 1.2 x 10^-4^ | 1.1 x 10^-4^ | *LRRC61, ACTR3C* | Intron, exon, 5’UTR; nc_intron |
|  | 1 | 35586333 - 35586674 | 5 | 2.2 x 10^-4^ | 1.1 x 10^-4^ | *ZMYM1* | intergenic |
|  | 6 | 31690879 - 31691564 | 18 | 1.8 x 10^-3^ | 2.5 x 10^-4^ | *MPIG6B* | TSS, intron, 5’UTR, cds |
|  | 3 | 145879405 - 145879735 | 6 | 3.6 x 10^-3^ | 1.1 x 10^-3^ | *PLOD2* | intergenic |

Differentially methylated regions were identified using comb-p from at-birth samples of babies born in the lower latitude (<50˚N). The inputs for comb-p were the annotated outputs from meta-analysis of summary results of EWAS of individual cohorts. CHR: chromosomes

^a^Reference season in EWAS of each cohort: autumn

**^b^**Regions with FDR-corrected Šidák p-value <0.05

**^c^**p-value for the most significant CpG in a DMR identified by comb-p

**Table S7**: Season of birth associated with differentially methylated regions (DMRs) in early childhood born in latitudes ≥50˚N

| **Season of birth^a^** | **CHR** | **Region** | **CpGs in**  **the region** | **Šidák**  **p-value^b^** | **Minimum p-value^c^** | **Gene** | **Gene group** |
| --- | --- | --- | --- | --- | --- | --- | --- |
| Winter |  |  |  |  |  |  |  |
|  | 1 | 153599454 - 153600181 | 8 | 1.6 x 10^-11^ | 2.8 x 10^-11^ | S100A13 | TSS, exon, 5’UTR; intron; TSS |
|  | 19 | 55476640 - 55477835 | 7 | 8.8 x 10^-11^ | 1.1 x 10^-6^ | NLRP2 | TSS, intron, exon, 5’UTR; nc_intron, nc_exon |
|  | 6 | 33871882 - 33873757 | 11 | 4.9 x 10^-8^ | 0.01185 | MIR7159 | intergenic |
|  | 6 | 31650710 - 31651387 | 21 | 1.0 x 10^-7^ | 1.3 x 10^-7^ | LY6G5C | intergenic |
|  | 20 | 62572850 - 62574298 | 7 | 1.1 x 10^-6^ | 8.8 x 10^-4^ | UCKL1, MIR1914, MIR647 | intron; nc_exon; cds; nc_gene; nc_intron |
|  | 13 | 47472025 - 47472454 | 12 | 2.0 x 10^-6^ | 8.9 x 10^-7^ | HTR2A | TSS, intron, exon, 5’UTR |
|  | 1 | 59042720 - 59043395 | 10 | 2.4 x 10^-5^ | 1.9 x 10^-6^ | TACSTD2 | TSS, cds |
|  | 17 | 73512294 - 73512880 | 8 | 3.9 x 10^-5^ | 2.4 x 10^-6^ | TSEN54 | TSS, intron, 5’UTR, cds |
|  | 19 | 51165379 - 51165870 | 5 | 6.0 x 10^-5^ | 1.1 x 10^-5^ | SHANK1 | cds |
|  | 6 | 28226860 - 28227417 | 10 | 2.8 x 10^-4^ | 4.6 x 10^-5^ | NKAPL, ZKSCAN4 | TSS, cds; intron, exon, 5’UTR |
|  | 11 | 19736887 - 19737376 | 4 | 4.7 x 10^-4^ | 6.6 x 10^-5^ | NAV2 | intron |
|  | 10 | 30722633 - 30723917 | 9 | 7.7 x 10^-4^ | 6.2 x 10^-3^ | MAP3K8 | TSS, intron, exon, 5’UTR |
|  | 4 | 5021059 - 5021353 | 8 | 9.3 x 10^-4^ | 7.6 x 10^-5^ | CYTL1 | TSS, 5’UTR, cds |
|  | 19 | 4539918 - 4540358 | 4 | 1.4 x 10^-3^ | 1.6 x 10^-4^ | LRG1 | TSS+intron+5’UTR+cds |
|  | 2 | 106361625 - 106362050 | 4 | 1.6 x 10^-3^ | 1.6 x 10^-4^ | NCK2 | Intron, 5’UTR |
|  | 11 | 34460082 - 34460582 | 10 | 2.2 x 10^-3^ | 1.2 x 10^-4^ | CAT | TSS, 5’UTR, cds |
|  | 22 | 43505867 - 43506263 | 6 | 4.8 x 10^-3^ | 4.3 x 10^-4^ | BIK | intergenic |
|  | 6 | 31543144 - 31543711 | 12 | 5.5 x 10^-3^ | 7.5 x 10^-4^ | TNF | TSS, intron, 5’UTR, cds |
|  | 10 | 43572040 - 43572492 | 7 | 5.8 x 10^-3^ | 5.0 x 10^-4^ | RET | TSS, exon, 5’UTR |
|  | 11 | 67383352 - 67384065 | 8 | 8.4 x 10^-3^ | 8.7 x 10^-4^ | DOC2GP | intergenic |
|  | 17 | 19627987 - 19628446 | 4 | 9.0 x 10^-3^ | 7.1 x 10^-4^ | SLC47A2 | intergenic |
| Spring |  |  |  |  |  |  |  |
|  | 17 | 33759459 - 33760318 | 11 | 2.6 x 10^-9^ | 9.1 x 10^-7^ | SLFN12 | TSS, exon, 5’UTR; intron, 5’UTR |
|  | 1 | 153599454 - 153600997 | 9 | 9.4 x 10^-8^ | 6.1 x 10^-4^ | S100A1, S100A13 | TSS, intron, exon, 5’UTR |
|  | 1 | 43919837 - 43920289 | 9 | 1.5 x 10^-6^ | 1.1 x 10^-6^ | SZT2 | Exon, 3’UTR |
|  | 7 | 94284233 - 94284775 | 19 | 2.2 x 10^-5^ | 9.5 x 10^-6^ | SGCE | Intron, 5’UTR |
|  | 2 | 177014601 - 177015150 | 11 | 3.6 x 10^-5^ | 6.0 x 10^-6^ | MIR10B | nc_gene |
|  | 7 | 130131705 - 130132290 | 16 | 1.3 x 10^-4^ | 2.8 x 10^-4^ | MEST | TSS, intron, 5’UTR, cds |
|  | 11 | 2322475 – 2323108 | 16 | 1.8 x 10^-4^ | 2.9 x 10^-5^ | C11orf21 | nc_intron; intron, 5’UTR, cds |
|  | 6 | 163149142 - 163149478 | 4 | 1.2 x 10^-3^ | 1.8 x 10^-4^ | PACRG | Intron, 5’UTR, cds |
|  | 13 | 51417661 - 51418246 | 10 | 1.3 x 10^-3^ | 2.5 x 10^-4^ | DLEU7-AS1, DLEU7 | nc_intron; TSS, 5’UTR, cds |
|  | 10 | 133946649 - 133946906 | 3 | 4.8 x 10^-3^ | 4.2 x 10^-4^ | JAKMIP3 | Intron, cds |
|  | 6 | 33280027 - 33280596 | 16 | 5.6 x 10^-3^ | 9.5 x 10^-6^ | TAPBP | intron |
|  | 6 | 28129071 - 28129641 | 10 | 6.0 x 10^-3^ | 5.0 x 10^-4^ | ZNF192P1 | nc_intron, nc_exon |
|  | 3 | 193922012 - 193922400 | 4 | 6.6 x 10^-3^ | 6.5 x 10^-4^ | LINC02036 | nc_intron |
|  | 11 | 10715150 - 10715246 | 5 | 6.8 x 10^-3^ | 4.5 x 10^-3^ | IRAG1 | Exon, 5’UTR |
|  | 8 | 145501792 - 145501887 | 2 | 7.5 x 10^-3^ | 0.01111 | BOP1 | intron |
|  | 2 | 947142 – 947659 | 4 | 9.0 x 10^-3^ | 2.9 x 10^-3^ | SNTG2 | intron |
| Summer |  |  |  |  |  |  |  |
|  | 10 | 123355243 -123356361 | 9 | 1.8 x 10^-7^ | 1.4 x 10^-3^ | FGFR2 | nc_intron; TSS, intron, exon, 5’UTR |
|  | 6 | 33872812 -33873757 | 5 | 3.5 x 10^-6^ | 1.4 x 10^-3^ | MIR7159 | intergenic |
|  | 6 | 5851619 -5852019 | 5 | 8.9 x 10^-5^ | 2.7 x 10^-4^ | FARS2 | intergenic |
|  | 7 | 30635687 -30635914 | 4 | 6.1 x 10^-4^ | 6.2 x 10^-4^ | GARS1 | intron |
|  | 20 | 62693632 -62694030 | 7 | 1.9 x 10^-3^ | 1.4 x 10^-3^ | TCEA2 | Intron, exon, 5’UTR |
|  | 10 | 42862851 -42863533 | 6 | 2.5 x 10^-3^ | 6.2 x 10^-3^ | LOC441666 | nc_intron, nc_exon |
|  | 2 | 21266702 -21267237 | 9 | 2.6 x 10^-3^ | 1.4 x 10^-3^ | APOB | TSS, intron, 5’UTR, cds |
|  | 22 | 51016874 -51017457 | 9 | 2.7 x 10^-3^ | 1.4 x 10^-3^ | CHKB-CPT1B, CPT1B, CHKB | nc_intron; TSS, intron, exon, 5’UTR; 3’UTR |
|  | 17 | 154385 -154696 | 4 | 4.7 x 10^-3^ | 1.4 x 10^-3^ | RPH3AL | intron |
|  | 6 | 41394131 -41394894 | 4 | 7.6 x 10^-3^ | 2.0 x 10^-3^ | LINC01276 | intergenic |

Whole blood samples from children (age: 1-11 years). Differentially methylated regions were identified using comb-p. The inputs for comb-p were the annotated meta-analysed outputs from whole blood EWASs.

CHR: chromosomes; TSS: translation start site; UTR: untranslated region; cds: coding sequence

^a^Reference season in EWAS of each cohort: autumn

**^b^**Regions with **Šidák** p-value <0.05

**^c^**P-value for the most significant CpG in a DMR identified by comb-p
