# Supplementary material for: Analysis of DNA methylation at birth and in childhood reveals changes associated with season of birth and latitude
Source: Clin Epigenetics. 2023 Sep 11;15:148. doi: 10.1186/s13148-023-01542-5 (PMC10496224; doi:10.1186/s13148-023-01542-5)
Supplement: Supplementary file 9 — Additional file 9. “Trait Enrichment Analysis (EWAS Atlas)”. Trait names and the odds ratios for their association with CpG sites for all the models meta-analysed in this study. [file 13148_2023_1542_MOESM9_ESM.docx]

**Trait Enrichment Analysis (EWAS Atlas)**

Abbreviations: DMC: Number of probes that are differentially methylated in a trait; Background: Number of probes in the trait in EWAS Atlas. Traits were considered significant if the p-values for their odds ratios were less than 0.05.

**Season of Birth analysis (primary)**

*At-birth (spring-born)*


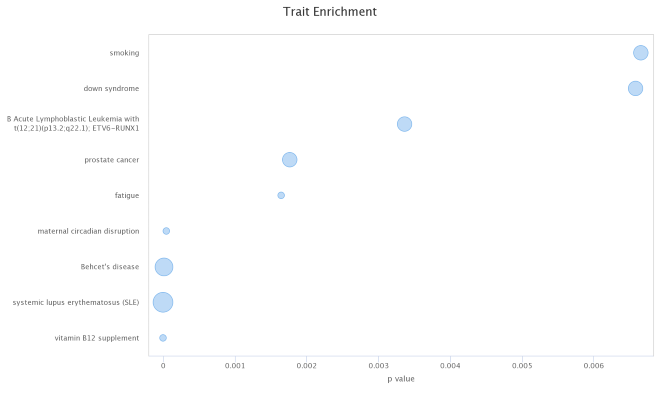


| **Trait** | **Odds Ratio** | **p Value** | **DMC** | **Background** |
| --- | --- | --- | --- | --- |
| vitamin B12 supplement | 74.248 | 2.82E-11 | 1 | 589 |
| systemic lupus erythematosus (SLE) | 16.551 | 4.34E-11 | 4 | 7848 |
| Behcet's disease | 13.734 | 2.54E-06 | 3 | 4364 |
| maternal circadian disruption | 49.2 | 4.36E-05 | 1 | 298 |
| fatigue | 35.941 | 1.64E-03 | 1 | 251 |
| prostate cancer | 6.457 | 1.77E-03 | 2 | 7047 |
| B Acute Lymphoblastic Leukemia with t(12;21)(p13.2;q22.1); ETV6-RUNX1 | 7.165 | 3.36E-03 | 2 | 4768 |
| down syndrome | 4.017 | 6.58E-03 | 2 | 14649 |
| smoking | 3.574 | 6.66E-03 | 2 | 20798 |

*At-birth (summer-born)*


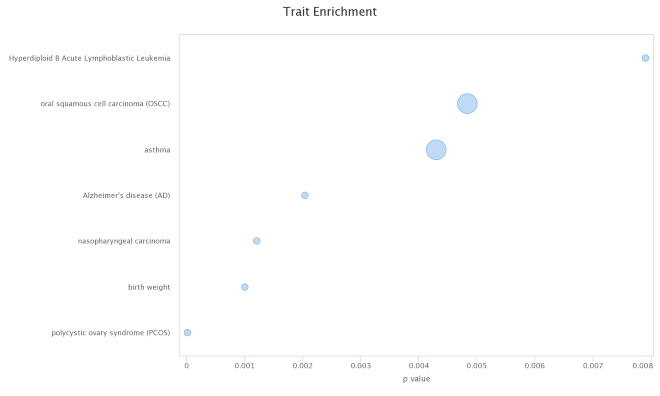


| **Trait** | **Odds Ratio** | **p Value** | **DMC** | **Background** |
| --- | --- | --- | --- | --- |
| polycystic ovary syndrome (PCOS) | 337.756 | 2.05E-05 | 1 | 137 |
| birth weight | 46.992 | 1.01E-03 | 1 | 973 |
| nasopharyngeal carcinoma | 42.867 | 1.21E-03 | 1 | 1068 |
| Alzheimer's disease (AD) | 32.836 | 2.04E-03 | 1 | 1392 |
| asthma | 6.88 | 4.30E-03 | 2 | 13639 |
| oral squamous cell carcinoma (OSCC) | 6.652 | 4.83E-03 | 2 | 14095 |
| Hyperdiploid B Acute Lymphoblastic Leukemia | 16.278 | 7.90E-03 | 1 | 2798 |

*At-birth (winter-born)*


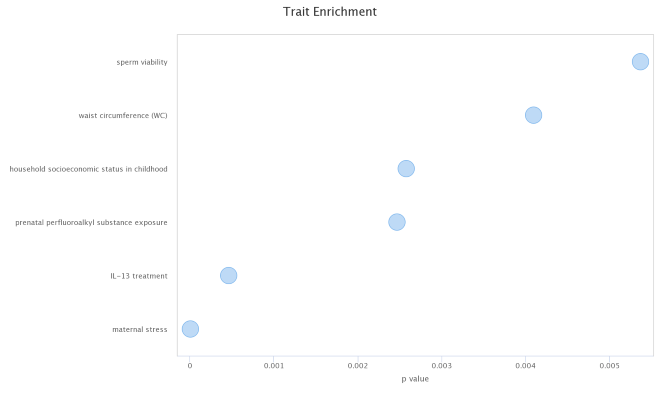


| **Trait** | **Odds Ratio** | **p Value** | **DMC** | **Background** |
| --- | --- | --- | --- | --- |
| maternal stress | 458.269 | 1.06E-18 | 1 | 88 |
| IL-13 treatment | 22.473 | 4.62E-04 | 1 | 1203 |
| prenatal perfluoroalkyl substance exposure | 29.777 | 2.46E-03 | 1 | 600 |
| household socioeconomic status in childhood | 29.139 | 2.57E-03 | 1 | 620 |
| waist circumference (WC) | 22.935 | 4.09E-03 | 1 | 766 |
| sperm viability | 19.925 | 5.37E-03 | 1 | 898 |

*Childhood (summer-born)*


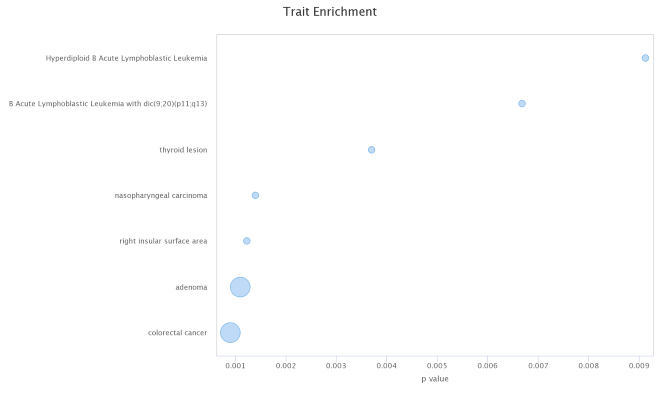


| **Trait** | **Odds Ratio** | **p Value** | **DMC** | **Background** |
| --- | --- | --- | --- | --- |
| colorectal cancer | 10.578 | 9.07E-04 | 2 | 8207 |
| adenoma | 10.026 | 1.10E-03 | 2 | 8647 |
| right insular surface area | 42.161 | 1.23E-03 | 1 | 1000 |
| nasopharyngeal carcinoma | 39.488 | 1.40E-03 | 1 | 1068 |
| thyroid lesion | 24.017 | 3.70E-03 | 1 | 1753 |
| B Acute Lymphoblastic Leukemia with dic(9;20)(p11;q13) | 17.659 | 6.69E-03 | 1 | 2379 |
| Hyperdiploid B Acute Lymphoblastic Leukemia | 15.008 | 9.13E-03 | 1 | 2798 |

**Latitude association (secondary analysis)**

**(Higher latitude, ≥50°N)**

*At-birth (summer-born)*


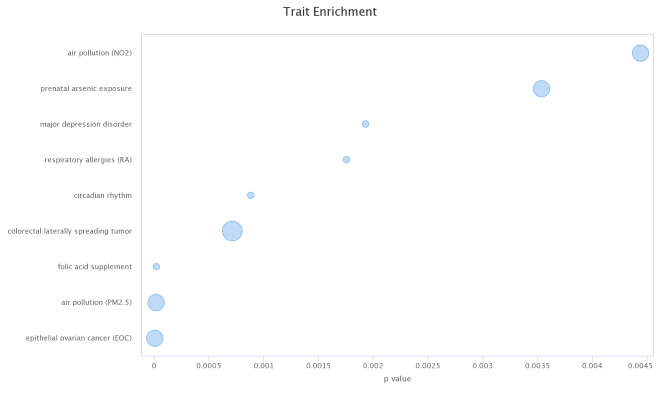


| **Trait** | **Odds Ratio** | **p Value** | **DMC** | **Background** |
| --- | --- | --- | --- | --- |
| epithelial ovarian cancer (EOC) | 41.252 | 4.05E-06 | 2 | 812 |
| air pollution (PM2.5) | 28.478 | 1.71E-05 | 2 | 1175 |
| folic acid supplement | 330.308 | 2.05E-05 | 1 | 51 |
| colorectal laterally spreading tumor | 6.095 | 7.12E-04 | 3 | 8245 |
| circadian rhythm | 48.428 | 8.81E-04 | 1 | 341 |
| respiratory allergies (RA) | 33.939 | 1.76E-03 | 1 | 485 |
| major depression disorder | 32.345 | 1.93E-03 | 1 | 509 |
| prenatal arsenic exposure | 6.822 | 3.53E-03 | 2 | 4860 |
| air pollution (NO2) | 6.39 | 4.44E-03 | 2 | 5185 |

**(Higher latitude, ≥50°N)**

*At-birth (winter-born)*


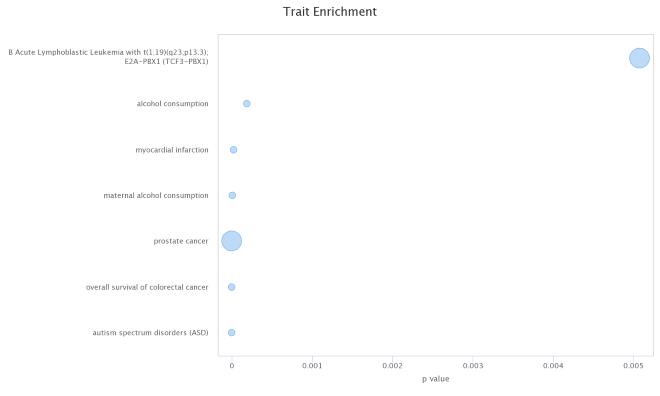


| **Trait** | **Odds Ratio** | **p Value** | **DMC** | **Background** |
| --- | --- | --- | --- | --- |
| autism spectrum disorders (ASD) | 116.002 | 3.14E-23 | 1 | 768 |
| overall survival of colorectal cancer | 911.31 | 7.13E-14 | 1 | 12 |
| prostate cancer | 13.875 | 2.59E-08 | 3 | 7047 |
| maternal alcohol consumption | 16.767 | 3.94E-06 | 1 | 2640 |
| myocardial infarction | 63.14 | 2.11E-05 | 1 | 214 |
| alcohol consumption | 15.919 | 1.83E-04 | 1 | 1816 |
| B Acute Lymphoblastic Leukemia with t(1;19)(q23;p13.3); E2A-PBX1 (TCF3-PBX1) | 3.796 | 5.07E-03 | 3 | 21099 |

**(Higher latitude, ≥50°N)**

*Childhood (spring-born)*


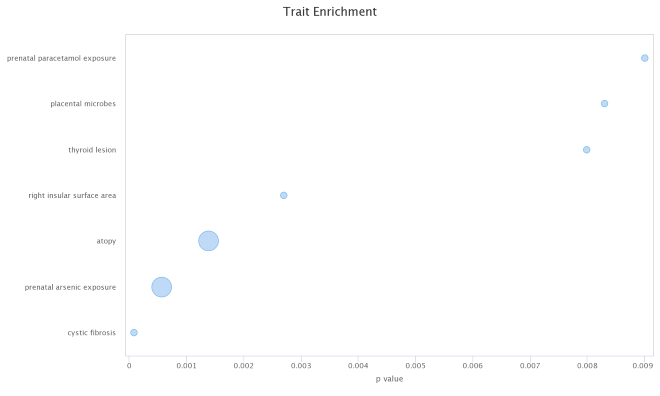


| **Trait** | **Odds Ratio** | **p Value** | **DMC** | **Background** |
| --- | --- | --- | --- | --- |
| cystic fibrosis | 160.474 | 8.53E-05 | 1 | 174 |
| prenatal arsenic exposure | 11.639 | 5.65E-04 | 2 | 4860 |
| atopy | 9.1 | 1.39E-03 | 2 | 6198 |
| right insular surface area | 27.719 | 2.70E-03 | 1 | 1000 |
| thyroid lesion | 15.777 | 7.99E-03 | 1 | 1753 |
| placental microbes | 15.458 | 8.31E-03 | 1 | 1789 |
| prenatal paracetamol exposure | 14.819 | 9.00E-03 | 1 | 1866 |

**(Higher latitude, ≥50°N)**

*Childhood (summer-born)*


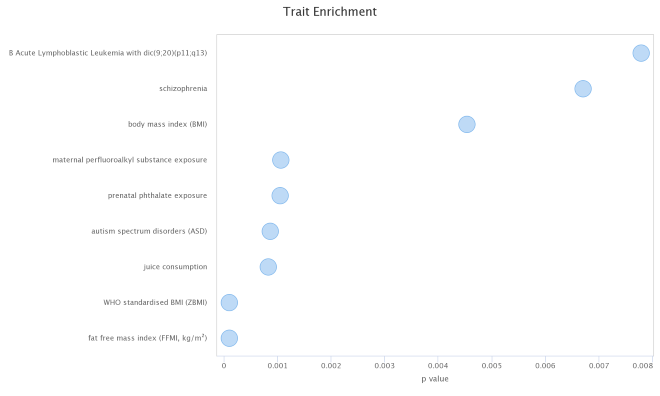


| **Trait** | **Odds Ratio** | **p Value** | **DMC** | **Background** |
| --- | --- | --- | --- | --- |
| fat free mass index (FFMI, kg/m²) | 155.891 | 9.26E-05 | 1 | 250 |
| WHO standardised BMI (ZBMI) | 155.891 | 9.26E-05 | 1 | 250 |
| juice consumption | 51.872 | 8.15E-04 | 1 | 749 |
| autism spectrum disorders (ASD) | 50.487 | 8.57E-04 | 1 | 768 |
| prenatal phthalate exposure | 45.622 | 1.05E-03 | 1 | 850 |
| maternal perfluoroalkyl substance exposure | 45.453 | 1.06E-03 | 1 | 854 |
| body mass index (BMI) | 21.525 | 4.53E-03 | 1 | 1797 |
| schizophrenia | 17.551 | 6.71E-03 | 1 | 2202 |
| B Acute Lymphoblastic Leukemia with dic(9;20)(p11;q13) | 16.239 | 7.78E-03 | 1 | 2379 |

**(Higher latitude, ≥50°N)**

*Childhood (winter-born)*


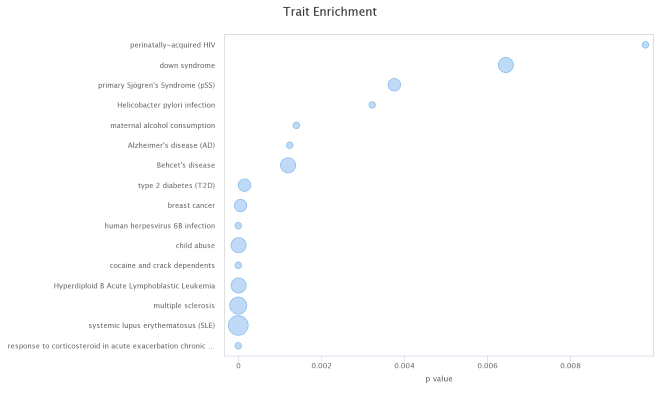


| **Trait** | **Odds Ratio** | **p Value** | **DMC** | **Background** |
| --- | --- | --- | --- | --- |
| response to corticosteroid in acute exacerbation chronic obstructive pulmonary disease patients | 794.857 | 3.16E-35 | 1 | 23 |
| systemic lupus erythematosus (SLE) | 11.46 | 3.06E-13 | 6 | 7848 |
| multiple sclerosis | 9.664 | 5.32E-11 | 4 | 8093 |
| Hyperdiploid B Acute Lymphoblastic Leukemia | 15.143 | 1.82E-10 | 3 | 2798 |
| cocaine and crack dependents | 54.447 | 6.47E-08 | 1 | 185 |
| child abuse | 10.849 | 4.44E-07 | 3 | 2924 |
| human herpesvirus 6B infection | 33.026 | 7.41E-07 | 1 | 406 |
| breast cancer | 8.404 | 3.81E-05 | 2 | 2888 |
| type 2 diabetes (T2D) | 5.876 | 1.32E-04 | 2 | 5633 |
| Behcet's disease | 5.491 | 1.19E-03 | 3 | 4364 |

**(Lower latitude, <50°N)**

*At-birth (spring-born)*


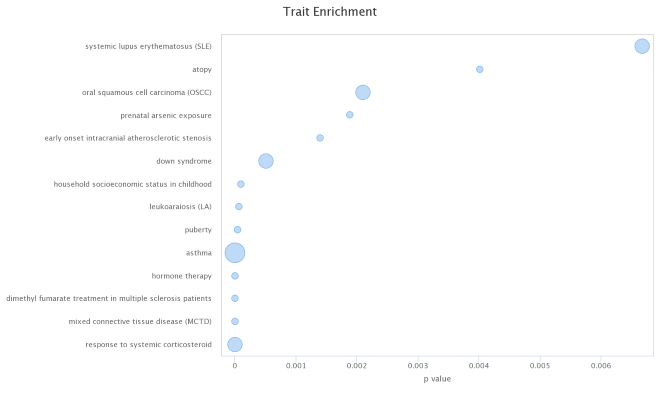


| **Trait** | **Odds Ratio** | **p Value** | **DMC** | **Background** |
| --- | --- | --- | --- | --- |
| response to systemic corticosteroid | 185.089 | 6.79E-14 | 2 | 219 |
| mixed connective tissue disease (MCTD) | 113.149 | 9.45E-08 | 1 | 182 |
| dimethyl fumarate treatment in multiple sclerosis patients | 112.834 | 9.56E-08 | 1 | 183 |
| hormone therapy | 58.079 | 1.31E-06 | 1 | 526 |
| asthma | 8.869 | 5.56E-06 | 4 | 13639 |
| puberty | 48.461 | 4.77E-05 | 1 | 445 |
| leukoaraiosis (LA) | 42.995 | 6.76E-05 | 1 | 531 |
| household socioeconomic status in childhood | 38.253 | 9.56E-05 | 1 | 620 |
| down syndrome | 5.909 | 5.07E-04 | 2 | 14649 |
| early onset intracranial atherosclerotic stenosis | 39.495 | 1.40E-03 | 1 | 318 |
